# Supplementary material for: Healthcare resource utilisation pattern and costs associated with herpes simplex virus diagnosis and management: a systematic review
Source: BMJ Open. 2022 Jan 4;12(1):e049618. doi: 10.1136/bmjopen-2021-049618 (PMC8728455; doi:10.1136/bmjopen-2021-049618)

APPENDIX

TABLE OF CONTENTS

Search methodology 2

Text 1: Keyword terms used in the search........ 3

Text 2: Keywords used in focused search using exploding terms........ 4

Table 1: Summary of included studies reporting healthcare costs and/or resource utilization related to HSV infection 5

Table 2: Detailed description of studies reporting cost (unit cost) 13

Table 3: Detailed description of studies reporting resource utilization 19

Table 4: Detailed cost incurred in genito-ulcer diseases due to HSV 22

Table 5: Detailed cost associated with genitoulcer disease prevention in people living with HIV 25

Table 6: Detailed cost associated with neonatal herpes prevention/management........ 26

Figure 1: Methodological quality of included economic studies using CHEC Checklist........ 30

Figure 2: Methodological quality of included costing studies using Larg and Moss Checklist ... 31

Search methodology

1. **Search strategy**

- The current search strategy was developed based upon keywords which have been used in previous existing HSV reviews commissioned by WHO. All search keywords used were subsequently cross-checked with the following articles to ensure comprehensiveness
    - Looker, 2017. Effect of HSV-2 infection on subsequent HIV acquisition: an updated systematic review and meta-analysis
    - Khard, 2019. The Epidemiology of Herpes Simplex Virus Type 1 in Asia: Systematic Review, Meta-analyses, and Meta-regressions
    - Looker,2012. Global estimates of prevalent and incident herpes simplex virus type 2 infections in 2012. PLoS One 2015;10(1) : e114989-e89. Doi: 10.1371/journal.pone. 0114989
- The following databases were identified for the search including: PubMed, PsychINFO, EMBASE, Centre for Review and Dissemination, EconLit, CEA registry and WHO Library Database (WHOLIS)

2. **Keywords search** was revised to compare between a) search including exploding terms and b) search including title and abstract. A total of 10,113 articles was found for search when terms were exploded versus 5,966 when these terms were not exploded. As such, the methods will only use search including exploding terms to minimize the risk of missing relevant study despite its low specificity. The initial search was performed in April 2019, with an updated search in October 2019.

3. **Neonate search**

- We also conducted search over again using all relevant HSV terms with neonate as keyword. All articles identified in the search overlapped with existing broader search, thereby there is no need to add neonate as key words

Text 1: Keyword terms used in the search

No.	Keyword
#1	Genital ulcer disease.mp.
#2	Herpes labialis.mp.
#3	Herpes genitalis.mp.
#4	Genital herpes.mp.
#5	Herpesvirus.mp.
#6	Herpes virus.mp.
#7	HSV.mp.
#8	Herpes simplex.mp.
#9	1 or 2 or 3 or 4 or 5 or 6 or 7 or 8
#10	Healthcare util*ation.mp.
#11	Util*ation.mp.
#12	Physician visit.mp.
#13	General practitioner visit.mp.
#14	Hospital visit.mp.
#15	Clinic visit.mp.
#16	Hospital stay.mp.
#17	Hospitali*ation.mp.
#18	Hospital readmission.mp.
#19	Cost.mp.
#20	Cost-effectiveness.mp.
#21	Cost-utility.mp.
#22	Cost-benefit.mp.
#23	Cost-minimi*ation.mp.
#24	Counselling.mp.
#25	Seek care.mp.
#26	Behavio*r.mp.
#27	10 or 11 or 12 or 13 or 14 or 15 or 16 or 17 or 18 or 19 or 20 or 21 or 22 or 23 or 24 or 25 or 26
#28	9 and 27

Text 2: Keywords used in focused search using exploding terms.

No.	Keyword
#1	Genital ulcer disease.mp.
#2	Herpes labialis.mp.
#3	Herpes genitalis.mp.
#4	Genital herpes.mp.
#5	Herpesvirus.mp.
#6	Herpes virus.mp.
#7	HSV.mp.
#8	Herpes simplex.mp.
#9	1 or 2 or 3 or 4 or 5 or 6 or 7 or 8
#10	pregnancy.mp.
#11	pregnant.mp.
#12	c*esarean.mp.
#13	delivery.mp.
#14	10 or 11
#15	12 or 13
#16	9 AND 14 AND 15

Table 1: Summary of included studies reporting healthcare costs and/or resource utilization related to HSV infection

**Author, year Country**	**Population and setting**	**Study design**	**Study objective**	**Sample size**	**HSV-subtype**		**Cost data**		**Healthcare resource utilization**	
					**1**	**2**	**Healthcare delivery process**	**Treatment**	**Healthcare delivery process**	**Treatment**
**HSV genital ulcer disease among adults/adolescents**										
Almonte-Vega, 2020 USA[39]	General population aged 15-49 years old	Cost-analysis	To study the dynamics of HSV–2 transmission, control and impact of treatment policies	-		x		x		
Aslam, 2012 Canada[18]	Records of individuals in the Canadian Disease and Therapeutic Index (CDTI)	Retrospective study	To investigate the rates of diagnosed cases of GH in Canada from 2002 to 2007	652					x	
Desharnais, 1996 USA[44]	Adults with herpes diagnosis from the HCIA Clinical Pathways Data Base	Retrospective study	To describe patterns of antiviral drug use for patients hospitalized with chickenpox, herpes simplex, and herpes zoster infections, and also for a subgroup of herpes patients with severe infections (systemic infections, eye infections, encephalitis, hemorrhagic pneumonitis, and other severe conditions)	3011	x	x		x		x
Fisman, 2002 USA[38]	Individuals aged 15 to 39 years	Cost-effectiveness	To project the future burden of HSV-2 infection in the United States, using a	-		x	x	x		

			mathematical model that incorporated epidemiologic trends documented between 1976 and 1994							
Fisman, 2003 USA[52]	Heterosexual couples	Modelling study	To evaluate the projected cost effectiveness of strategies to prevent HSV-2 transmission in couples with no history of HSV-2 infection	-		x		x		
Fisman, 2005 Canada[19]	Individuals with recurrent genital ulcer	Prospective study	To estimate the impact on health-related quality of life associated with both symptomatic and asymptomatic GH	39	x	x				x
Gilbert, 2010 USA[20]	Young adults	Retrospective study	To investigate characteristics associated with GH screening and diagnosis in sexually active young adults aged 18 to 24	Add Health Data: 11,570 NCHA: 222,740	x	x			x	
Korenromp, 2017[37]	People 15-49 year old living with HSV-2	Modelling study	To estimate the costs of reaching the 2020 STI strategy milestones for the period 2016–2021, to support policy, planning, implementation, and future cost-benefit evaluation of the global STI strategy 2016–2021.	-		x	x	x		
Owusu-Edusei, 2013a USA[34]	People aged 15-25 years	Retrospective study	To examine the utilization and cost of the diagnostic methods used for STI screening among	-		x	x			

			privately insured adolescent and young adult population							
Owusu-Edusei, 2013b USA[30]	-	Cost of illness analysis	To update the estimates of lifetime direct medical cost for 8 major STI	-				x		
Patrick, 2004 Worldwide survey from 78 countries[21]	Subjects with genital herpes	Survey	To describe patient experiences and views regarding genital herpes management	2075	x	x			x	x
Szucs, 2001 USA[31]	General population	Economic analysis	To estimate the economic burden of GH in the USA, using two different costing approaches	465,075			x	x		
Tao, 2000 USA[22]	General population	Cost-of-illness analysis	To assess the US direct medical expenditures for genital herpes and its complications to assist policy makers in allocating limited STD resources efficiently	-		x			x	
Vickerman, 2008 UK[33]	-	Cost-effectiveness	To compare the cost per ulcer treated of using the 1994 and 2003 algorithms amongst individuals presenting with GUD	-		x		x		
Vickerman, 2011 South Africa[32]	HIV+ women	Cost-effectiveness	To estimate the cost-effectiveness of daily acyclovir for delaying HIV-1 disease progression in women not eligible for antiretroviral therapy (ART)	300		x		x		

Xia, 2018 United States[40]	General population	Retrospective study	To determine the utilization and cost burden associated with HSV infection visits to U.S. EDs in recent years from 2006-2013	704,728			x		x	
**Neonatal herpes prevention among pregnant women**										
Baker, 2004 USA[42]	-	Cost-effectiveness	To determine whether serologic testing for herpes simplex virus type 2 (HSV-2) in pregnant women and their partners is cost-effective	100,000		x		x	x	x
Barnabas, 2002[29] USA	-	Cost-effectiveness	To assess the potential effectiveness, cost effectiveness, and benefit of suppressive therapy among herpes simplex virus serodiscordant sex partners during pregnancy		x	x	x	x		
Binkin, 1989 USA[43]	Pregnant women with HSV	Cost-effectiveness	To present a reanalysis of the cost effectiveness of maternal herpes screening and a review of the changes that have occurred in the screening recommendations since 1980	3,600,000	x	x	x	x	x	
Brocklehurst, 1995 UK[23]	All members and Fellows of the Royal College of Obstetricians and Gynaecologist resident	Survey	To determine the clinical practice among obstetricians in the antepartum and intrapartum management of women with recurrent genital herpes infection	2252	x	x			x	x
Brown, 2003 USA[27]	Pregnant women from university,	Cohort study	To determine the effects of viral shedding, maternal HSV	58362	x	x				x

	army and community hospitals		serological status and delivery route on risk of transmission of HSV from mother to infant							
Heggarty, 2020 France[28]	Healthcare providers for pregnant women	Survey	To evaluate health care provider knowledge, and collect information on management of genital herpes during pregnancy and infants born to mothers with herpes	354	x	x			x	x
Kenny, 2013 Canada[26]	Obstetrician, gynaecologist and family physicians offering maternity care practicing in Alberta	Survey	To identify the practice patterns of physicians providing prenatal care in Alberta with respect to prevention of neonatal HSV infection, including their prescribing of antiviral therapy to pregnant women in the third trimester.	183	x	x			x	x
Little, 2005 USA[46]	Women with a history of diagnosed genital HSV	Cost-effectiveness	To determine the clinical benefits and cost-effectiveness of prophylactic acyclovir in women with a history of HSV but no recurrence during pregnancy	-	x	x		x		x
Lynn, 2017 Ireland[24]	Pregnant women with genital HSV from a university hospital	Antenatal chart review	To describe the HSV management in pregnancy at a joint antenatal genital maternity hospital	107	x	x			x	x
Randolph, 1996 USA[47]	Antenatal women with recurrent genital HSV	Cost-effectiveness	To compare the cost-effectiveness of oral acyclovir prophylaxis in late pregnancy compared to caesarean delivery for genital herpes	10,000			x	x		

			lesions in the prevention of neonatal herpes transmission from mothers with recurrent genital infections							
Rouse, 2000 USA[48]	Antenatal women	Cost-effectiveness	To evaluate the potential cost effectiveness of herpes simplex virus antibody screening	8,538	x	x	x	x	x	
Scott, 1998 USA[35]	-	Cost-effectiveness	To determine whether acyclovir suppression provides a greater cost savings over no medical therapy in the management of recurrent genital herpes (HSV) in pregnancy	-	x	x	x	x		
Stankiewicz Karita, 2017 USA[25]	Pregnant women from a hospital	Retrospective study	To investigate the frequency of invasive obstetric procedures and caesarean deliveries for women with known HSV infection	449		x			x	
Thung, 2005 USA[49]	Married women	Cost-effectiveness	To determine the cost-effectiveness of routine antenatal screening for HSV-1 and HSV-2 in women without a known history of genital herpes.	100,000	x	x		x		
Tuite, 2010 Canada[36]	Pregnant women	Cost-effectiveness	To assess the effectiveness and cost effectiveness of identifying pregnant women at risk of de novo HSV acquisition to prevent vertical HSV transmission	100,000	x	x	x	x		

**Neonatal herpes management**										
Ahmad, 2015 USA[15]	Neonates who sought care in emergency department	Retrospective study	To evaluate whether guideline implementation affected the ED's decision to test for HSV, ED use of HSV polymerase chain reaction (PCR) and acyclovir	308	x	x				x
Ambroggio, 2009 USA[41]	Neonates with HSV and received intravenous acyclovir and discharge from Paediatric Health Information System	Retrospective study	To quantify the economic burden of neonatal HSV during initial hospitalization while focusing on factors, such as congenital anomalies and HSV-associated complications, which increase hospital charges and length of hospital stay among neonates with HSV	406	x	x		x		x
Bernard, 2013 France[16]	Patients aged 28 days and above from the French national hospital discharge database	Prospective study	To compare the data from the French national hospital discharge database (Programme de Me´ dicalisation des Syste` mes d'Information; PMSI) and from the prospective study conducted in 2007 and evaluate the reliability of PMSI as a tool to assess the trends of encephalitis in France	1,947	x	x				x
Donda, 2019 USA[45]	Neonates with ICD-9 codes for neonatal HSV in the National	Retrospective study	To examine the temporal trends in the incidence and outcomes of neonatal HSV in the United States	42,726,336				x		x

	Inpatient Sample from 2003-2014									
Flagg, 2011 USA[51]	Inpatient records of infants aged 60 days or younger from the Healthcare Cost and Utilization Project Kids' Inpatient Database	Retrospective study	To estimate the incidence of HSV infections for the United States during 2006, as well as demographic-specific rates, by using nationally and regionally weighted estimates from a population-based sample of inpatient data	4,106,488	x	x		x		x
Mahant, 2019 USA[50]	Records of neonates from the Medicaid claims database from 2009 - 2015	Retrospective study	To examine the incidence, mortality, and health care use related to neonatal herpes HSV infection.	2,107,124				x		x
Owusu-Edusei, 2015 USA[17]	Insurance claim data on inpatient admission from the Truven Health Analytics MarketScan Commercial Claims and Encounters Database	Cost-of-illness analysis	To estimate the average excess inpatient cost of neonatal herpes simplex virus (NHSV) infection from 2005 to 2009 insurance claims data	474,743	x	x				x

Table 2: Detailed description of studies reporting cost (unit cost)

Author, year Country	Population and setting	Diagnostic costs (range)	Treatment costs* in original year of value (range)	Hospitalisation costs (range)	Other healthcare delivery costs (range)	Lifetime management cost (range)
**Genital ulcer disease among adults/adolescents**						
Almonte-Vega, 2020 USA[39]	General population aged 15-49 years old	Microbiological lab test (unspecified): $80.17	Acyclovir treatment (duration not specified): $86.33	NR	Consultation, clinical examination and diagnostic: $161.85	NR
Desharnais, 1996[44]	Adults with herpes diagnosis identified from the HCIA database	NR	Total drug charges: $1941<br>Antiviral drug charges (not specified): $1070	Hospital charges: $5637	NR	NR
Fisman, 2002[38]	Individuals aged 15 to 39 years	NR	Cost of treatment for primary syndrome<br>Male: $470 ($370-5$60)<br>Female: $830 ($670-$1000)<br><br>Antiviral therapy<br>Relapse: $17 ($9-$36)<br>Monthly suppressive therapy: $40 ($20-$220)	NR	Clinic visit: $120 ($90-$150)<br>Obstetrical care: $310 ($130-$800)	Initial cost of caring for neonates with HSV: $42,600<br>Lifetime medical and long-term care cost for infants with moderate neurological sequalae: $97,000<br>Lifetime medical and long-term care cost for infants with severe neurological sequalae: $291,000

Fisman, 2003[52]	Heterosexual couples	Western blot: $60 ($45-$90)<br>ELISA: $5 ($3-$35)	Cost of treatment for primary syndrome<br>Male: $450 ($360-5$40)<br>Female: $800 ($640-$960)<br><br>Acyclovir (per episode): $16 ($9-$35)<br>Acyclovir (monthly suppressive): $40 ($20-$215)	NR	Clinic visit: $120 ($90-$145)<br>Labour: $120 ($90-$145)	Lifetime cost of care of neonatal HSV-2: $110,000 ($85,000-$860,000)
Korenromp, 2017[37]	People 15-49 year old living with HSV-2	NR	Acyclovir 400mg per tab: $0.04	NR	Treatment service delivery (not specified): $10<br>Procurement cost: $0.21	NR
Owusu-Edusei, 2013a[34]	People aged 15-25 years	Laboratory test (unspecified): $24.30-27.05	NR	NR	NR	NR
Owusu-Edusei, 2013b[30]	-	NR	NR	NR	NR	Lifetime medical cost per case, median(range):<br>Men: $761 (381-1,142)<br>Women: $621(311 - 932)<br>Lifetime cost of new infections acquired in 2008: $435.9 million

Szucs, 2001[31]	General population	Laboratory test: $1.5-76.50	Drug: $64-131	Hospitalisation: $669	Labour: $39.8 -62.6<br>Clinic visit: $36.20-73<br>Day off work: $144	NR
Vickerman, 2008[33]	-	NR	Acyclovir 200mg tds for 5 days: $0.53- 5.24	NR	Counselling cost: $0.28	NR
Vickerman, 2011[32]	HIV+ women	NR	Acyclovir 400mg: $0.07<br>Yearly ART cost: $1700 (1359-2000)	NR	Staff costs/women 3m treatment cycle: $15.60	NR
Xia, 2018[40]	General population	NR	NR	ED: $1,069		
**Neonatal herpes prevention among pregnant mothers**						
Baker, 2004[42]	-	Laboratory test with labor cost for HSV-2: 15.58 – 60.00	Average antiviral daily cost (assuming 50% on generic acyclovir 400mg tds and 50% on valacylovir qd): $1.70-7.90<br>Acyclovir 400mg: $0.366- 1.955<br>Valacyclovir 500mg/tab: $3.95<br>Valacyclovir 1g/tab: $6.49	Delivery: $4,779-22,838	Labour cost: $15.58 – $60<br>Counselling cost: $5.98-$6.67	Lifetime cost of care of neonatal HSV: $54,516- $129,576

Barnabas, 2002[29]	-	Diagnostic cost: $16-$100	Drug cost per couple per pregnancy: $37<br>Acute neonatal herpes treatment $1,500-50,000	C/S cost (personnel, supplies, surgery and ward care): $11,084	Labour cost: $200-1628<br>Counselling cost: $12-$19	Neonatal care after C/S: $884<br>Long term care for neonatal herpes: $140,766 - $273,712
Binkin, 1989[43]	Pregnant women with HSV	Viral culture: $30	NR	Hospitalisation for complication: $300-698<br>Hospital care associated with neonatal herpes: $25,000<br>Delivery: $2,300-3,600	NR	Long term care for neonatal herpes: $125,000-$250,000
Little, 2005[46]	Women with a history of diagnosed genital HSV	NR	Acyclovir (prophylaxis) from 36 weeks of gestation: $46	Delivery: $4,939-9,490<br>Hospitalisation: $32,483	NR	Lifetime cost of care of neonatal HSV: $349,7533-$1,049,260
Randolph, 1996[47]	Antenatal women with recurrent genital HSV	Laboratory: $35	Acyclovir 400mg (200caps): $228	Delivery: $3,500	Labour: $74	Lifetime cost of care of neonatal HSV: $85,000- 255,000
Rouse, 2000[48]	Antenatal women	Laboratory: $4 – 13	NR	Hospitalisation for neonatal care: $11,126	Labour: $3.50-10.50	Lifetime cost of care of neonatal HSV: $48,519- 163,879
Scott, 1998[35]	-	HSV culture: $80	Acyclovir 400mg tds for 4 weeks: $180	Hospitalisation for neonatal care: $480-1470<br>Delivery: $5,321 – 9,039	NR	NR

Thung, 2005[49]	Married women	HSV screening: $37.5-$75	Acyclovir 400mg tds for 4 weeks: $71	Delivery: $4,281 - 9,283	Counselling cost: $13	Lifetime cost of care of neonatal HSV: $13,202 – 325,602
Tuite, 2010[36]	Pregnant women	ELISA test: $7-$14	NR	Delivery: $5680- 8780	NR	Lifetime cost and consequence of neonatal HSV: $164,870
**Neonatal herpes management**						
Ambroggio, 2009[41]	Neonates with HSV and received intravenous acyclovir and discharge from Paediatric Health Information System	NR	Median pharmaceutical (not specified): $4,231<br>Median Imaging: $2,010	Median hospital charge: $37,431	NR	NR
Donda, 2019[45]	Patients aged 28 days and above from the French national hospital discharge database	NR	NR	Hospitalisation: $27,843	NR	NR
Flagg, 2011[51]	Neonates with ICD-9 codes for neonatal HSV	NR	NR	Hospitalisation: $92,664	NR	NR

	in the National Inpatient Sample from 2003-2014					
Mahant, 2019[50]	Records of neonates from the Medicaid claims database from 2009 - 2015	NR	NR	Hospitalisation: $32,683<br>Hospital readmission: $31,531<br>ED visit: $527	NR	NR

*All costs are mean costs except where explicitly labelled as median costs.

C/S – Caesarean section; ED - Emergency department; NR – Not reported

Table 3: Detailed description of studies reporting resource utilization

Author, year	Healthcare seeking and diagnosis	Treatment phase
**Genital ulcer disease among adults/adolescents**		
Aslam, 2012[18]	• 74.1-93.2% sought care once within 12 months<br>• 6.8-25.9% sought care twice to 8x a year	
Desharnais, 1996[44]		• Oral treatment only: 16.1%<br>• IV treatment: 16.2%<br>• Hospital stay: 5.4 days
Fisman, 2005[19]		• 33.3% used antiviral drugs for HSV<br>• 15.8% had pregnancy complicated by HSV
Gilbert, 2010[20]	• 1.32% of young adults ever tested for genital herpes	
Patrick, 2004[21]	• 49% had viral culture performed<br>• 9% had antibody test<br>• 34% had physical examination	• 65% received oral antiviral therapy<br>• 18% received topical antiviral therapy<br>• 17% obtained alternative therapy
Tao, 2000[22]	• Estimated annual genital herpes visit 499,655 yearly<br>• 2% were inpatient visit<br>• 9% outpatient & ED visit<br>• 20% public STD clinic<br>• 69% private office based visit	
Xia, 2018[40]	From 2006-2013<br>• 245,484 ED visits with primary diagnosis of genital herpes or 37.3% of total ED visits for HSV<br>• Total charges: $278,335,295<br><br>ED visits trend from 2006 – 2013<br>• 24,747 (33.8%); 26,440 (34.1%); 27,484 (36.1%), 28,440 (36.5%); 33,258 (37.8%); 33,095 (38.3%); 35,501 (40.0%); 36,518 (40.3%)	
**Neonatal herpes prevention among pregnant mothers**		
Baker, 2004[42]	Estimates used in model<br>• 75% of partners will be willing to undergo HSV screening	Estimates used in model<br>• 1.32% women HSV-2 negative acquiring HSV during last 8 weeks of pregnancy<br>• 57% women or partner offered and accept antiviral therapy with testing

		• 82% women taking antivirals from week 36 compliant
Binkin, 1989[43]	Estimates used in model<br>• Average number of cultures per patient: 8	
Brocklehurst, 1995[23]	• 60% of obstetricians advocated some form of antenatal screening<br>Among those performing screening<br>• 64% perform regular viral cultures<br>• 54% recommend screening <u><</u>34 weeks of gestation	• 92% of providers: visible active lesions at labor are cause for caesarean delivery
Brown, 2003[27]		• All women with HSV genital lesions noted at delivery had caesarean delivery (n=60) unless lesions not noted until too late to proceed with caesarean or lesions noted after delivery (n=14)
Heggarty, 2020[28]	For suspected primary genital HSV:<br>▪ 43.3% would conduct PCR of lesions plus HSV serology<br>▪ 39.9% would conduct PCR of lesions alone<br>▪ 0.4% would conduct HSV serology only	• If primary HSV GUD during pregnancy, 68.4% "always" and 11.6% "often" prescribe antiviral prophylaxis in 3rd trimester<br>• If recurrent HSV GUD during pregnancy, 55.1% "always" and 12.9% "often" prescribe antiviral prophylaxis in 3rd trimester<br>• 83% recommend caesarean delivery if genital HSV lesions suspected during labour
Kenny, 2013[26]	• 30% physicians will perform type-specific serology "most of the time" for patients with no history of herpes but partner with known HSV	• Antiviral suppressive therapy prescribed in third trimester by 90% of doctors (97% of obstetricians and 84% family physicians)<br>▪ 62% prescribed for any past history of GUD including pre-pregnancy<br>▪ 28% only after outbreak during pregnancy<br>▪ More commonly prescribed acyclovir (63%) than valacyclovir (38%)<br>• 65% offer elective caesarean if primary HSV in third trimester<br>• 95% of obstetricians and 84% of family physicians recommend caesarean delivery if HSV lesions during labour
Little, 2005[46]		Estimates used in model<br>• 24% of women will undergo caesarean delivery if no lesion was present

Lynn, 2017[24]	• 89% of patients had type-specific serology sent	• 63% received antiviral prophylaxis<br>▪ 98.5% received valacyclovir<br>▪ 1.5% received acyclovir<br>▪ Mean for initiating: week 36<br>• 29% of patients underwent caesarean delivery, none for HSV
Rouse, 2000[48]	Estimates used in model<br>• 75% of partners will be willing to undergo HSV screening	
Stankiewicz Karita, 2017[25]		• Antiviral suppressive therapy:<br>▪ 55% HSV-2 antibody-positive only<br>▪ 65% history of symptomatic GUD<br>• Similar caesarean section rates for women with/without history of HSV/genital herpes:<br>▪ 25% without history of HSV-2/GH<br>▪ 30% on suppressive treatment<br>• 28% without suppressive treatment
**Neonatal herpes management**		
Ahmad, 2015[15]	• CSF PCR performed in 92.3%<br>• Blood PCR performed in 48.7%	• 9.4 – 9.8% require ICU stay<br>• Hospital stay: 83.1-84.6hr<br>• 71.8% received acyclovir
Ambroggio, 2009[41]		• Median length of stay: 13 days (IQR 4-21)
Bernard, 2013[16]		• Mean hospital admission: 28 -34 days
Donda, 2019[45]		• Median length of stay: 20
Flagg, 2011[51]		• Mean length of stay: 22 days<br>• Median length of stay: 2- days
Mahant, 2019[50]		• Median hospital stay: 18 days<br>• Post discharge,<br>○ 45.7% required ED visit<br>○ 16.2% required rehospitalisation
Owusu-Edusei, 2015[17]		• Mean hospital stay: 10.8 (11.5)<br>• Mean hospital stay among those with admission >7 days: 18.5 (12.5)

Table 4: Detailed cost incurred in genito-ulcer diseases due to HSV

Author,year	Outcomes	Unit cost ($) in original year	Unit cost in 2018 ($)
**Medication costs**			
Vickerman, 2008	One dose of IV benzathine penicillin 2.4MU	0.15 - 0.48	0.19-0.59
Vickerman, 2008	One tab of 500mg ciprofloxacin	0.10 - 0.21	0.12 - 0.26
Vickerman, 2008	One cap of 200mg acyclovir	0.53- 5.24	0.66 – 6.48
Fisman, 2003	Acyclovir therapy for relapse patients	16.00	22.72
Fisman, 2003	Acyclovir cost for suppressive monthly therapy	40.00	56.80
Almonte-Vega, 2020	Acyclovir therapy	86.33	86.33
Fisman, 2003	Condom cost	0.10	0.14
Szucs, 2001	Pharmacological treatment 1st episode (NS)	64.00	94.86
Szucs, 2001	Pharmacological treatment recurrent episode (NS)	131.00	194.18
Vickerman, 2008	Needle and syringe cost	0.15	0.19
Tao, 2000	Pharmacy claim	52.00	73.84
**Laboratory test**			
Szucs, 2001	Antibiotic testing based on expert opinion	76.50	113.39
Szucs, 2001	Antibiotic testing in first episode based on claims	12.80	18.97
Szucs, 2001	Antibiotic testing in subsequent episode based on claims	6.50	9.63
Szucs, 2001	Complete blood count based on expert opinion	21.29	31.56
Szucs, 2001	Complete blood count in first episode based on claims	4.60	6.82
Szucs, 2001	Complete blood count in subsequent episode based on claims	1.50	2.22
Szucs, 2001	Microbiological test for first GUD episode	17.60	26.09
Szucs, 2001	Microbiological test for subsequent GUD episode	6.70	9.93
Szucs, 2001	Microbiological test based on expert opinion	38.39	56.90
Almonte-Vega, 2020	Microbiological lab test	80.17	80.17

Fisman, 2003	Western blot	60.00	85.20
Szucs, 2001	Urine analysis based on expert opinion	12.59	18.66
Szucs, 2001	Urine analysis in first episode based on claims	4.60	6.82
Szucs, 2001	Urine analysis in subsequent episode based on claims	3.20	4.74
**Hospitalisation cost**			
Fisman, 2003	Excess obstetrical cost associated with history of symptomatic HSV2 infection	300.00	425.98
Fisman, 2003	Excess obstetrical cost due to symptomatic HSV2 infection	310.00	440.18
Tao, 2000	Inpatient cost	2,530.00	3592.46
Szucs, 2001	Hospital day	669.00	991.63
**Clinic visit**			
Fisman, 2003	Clinic visit related to GUD (for physician time, test, lost wages due to 2hr patient time)	120.00	170.39
Szucs, 2001	Clinical examination based on expert opinion	40.33	59.78
Szucs, 2001	Clinical examination first episode based on claims	39.80	58.99
Szucs, 2001	Clinical examination on subsequent episode based on claims	36.20	53.66
Szucs, 2001	Physician consultation based on expert opinion	73.00	108.21
Szucs, 2001	Physician consultation in first episode based on claims	62.60	92.79
Szucs, 2001	Physician consultation in subsequent episode based on claims	59.60	88.34
Tao, 2000	Outpatient and ED	59.00	83.78
Fisman, 2003	Outpatient visit	120.00	170.39
Tao, 2000	Office based physician and public clinic	67.00	95.14
Almonte-Vega, 2020	Consultation, clinical examination and diagnostic	161.85	161.85
Vickerman, 2008	Counselling cost	0.28	0.35
**Other costs**			
Szucs, 2001	Others miscellaneous cost related to first GUD episode(not reported)	33.00	48.91

Szucs, 2001	Others miscellaneous cost related to recurrent GUD episode(not reported)	12.30	18.23
Szucs, 2001	Production losses	60.00	88.94
Szucs, 2001	Total cost of active GUD	355.00	526.20
Szucs, 2001	Total cost of incident GUD	235.00	348.33
Szucs, 2001	Total cost of prevalent GUD	166.00	246.06
Szucs, 2001	Total cost of recurrent GUD	499.00	739.65
Fisman, 2003	Treatment cost for men assuming 2 clinic visit, 7 day course of acyclovir (400mg tds) and 2 days off work	450.00	638.97
Fisman, 2003	Treatment cost for women assuming 2 clinic visit, 7 day course of acyclovir (400mg tds) and 2 days off work	800.00	1135.95

Table 5: Detailed cost associated with genitoulcer disease prevention in people living with HIV

Author, year	Outcomes	Unit cost ($) in original year	Unit cost in 2018 ($)
Vickerman, 2011	Acyclovir 400mg	0.07	0.07
Vickerman, 2011	Staff cost- for default tracer over 3 months	24.00	22.32
Vickerman, 2011	Staff cost for training for STI diagnosis and default tracer	0.46	0.43
Vickerman, 2011	Labour cost for senior nurse	2.52	2.34
Vickerman, 2011	Counselling cost (10 mins)	0.88	0.82
Vickerman, 2011	CD-4 count test	7.90	7.35

NB- Cost reported after adjustment in 2017 were lower than those in the original study due to exchange rates at the time of study.

Table 6: Detailed cost associated with neonatal herpes prevention/management

Author,year	Outcomes	Unit cost (USD$)	Unit cost in 2017 ($)
**Medication costs**			
Randolph, 1996	One cap of acyclovir 400mg	1.14	1.72
Baker, 2004	Pharmaceutical cost for pregnant women	6.18	8.10
Baker, 2004	Pharmaceutical cost for partner	3.93	5.15
Baker, 2004	Valacyclovir 500mg	3.95	5.18
Baker, 2004	Valacyclovir 1000mg	6.49	8.51
Baker, 2004	Acyclovir 400mg	1.96	2.57
Barnabas, 2002	Acyclovir treatment for a couple for one pregnancy	37.00	51.37
Scott, 1998	Acyclovir 400mg	1.71	2.58
**Laboratory test**			
Randolph, 1996	Screening using herpes culture	35.00	52.83
Thung, 2005	HSV1 or 2 screening cost	37.50	49.15
Thung, 2005	HSV 1 and 2 screening	75.00	98.31
Rouse, 2000	HSV-2 antibody assay	4.00	5.68
Rouse, 2000	HSV-2 labour and reagent cost, QC etc	9.00	12.78
Tuite, 2010	ELISA screening for HSV	7.00	7.96
Scott, 1998	HSV culture	80.00	120.75
Baker, 2004	Labor and supplies for HSV-2 specific test	15.58	20.42
Baker, 2004	HSV test for partner	40.53	53.12
Barnabas, 2002	Diagnostic kit cost	70.00	97.18
Binkin, 1989	Viral culture	30.00	52.97
**Hospitalisation cost**			
Scott, 1998	Vaginal delivery with metritis, includes labour, delivery, postpartum and professional	8439.00	12,737.15

Scott, 1998	Vaginal delivery without metritis, includes labour, delivery, postpartum and professional	5,321.00	8,031.09
Ambroggio, 2009	Hospital charges	62,050.90	70,544.69
Tuite, 2010	Vaginal delivery	5,680.00	6,457.50
Little, 2005	Vaginal delivery	4,939.00	6,104.17
Randolph, 1996	Caesarean delivery over vaginal	3,500.00	5,282.62
Tuite, 2010	Caesarean section	8,780.00	9,981.84
Tao, 1999	Caesarean attributable to genital herpes	1,922.00	2729.13
Little, 2005	Caesarean delivery	9,490.00	11,728.80
Little, 2005	Caesarean delivery with lesion	7,608.00	9,402.82
Scott, 1998	Caesarean delivery with metritis, includes labour, delivery, postpartum and professional	9,039.00	13,642.74
Scott, 1998	Caesarean delivery without metritis, includes labour, delivery, postpartum and professional	10,553.00	15,927.85
Thung, 2005	Elective caesarean	7,425.00	9,732.37
Thung, 2005	Labour caesarean	9,283.00	12,167.75
Little, 2005	Hospital care due to neonatal herpes infection	32,483.00	40,146.12
Rouse, 2000	Hospital care due to neonatal herpes infection	11,126.00	15,798.28
Baker, 2004	Caesarean delivery	5,021.00	6,581.31
Binkin, 1989	Hospital stay due to complication	698.00	1,232.38
Binkin, 1989	Hospital care due to neonatal herpes infection	25,000.00	44,139.53
Barnabas, 2002	Caesarean delivery with lesion	11,084.00	15,388.48
**Clinic visit**			
Scott, 1998	Clinic visit	39.50	59.62
Thung, 2005	Counselling cost	13.00	17.04
Rouse, 2000	Counselling cost (10 mins)	3.50	4.97
Rouse, 2000	Counselling cost for couple (30 mins)	10.50	14.91
Randolph, 1996	Follow-up call and office visit following screening	74.00	111.69
Barnabas, 2002	Pharmacy dispensing and education cost	3.00	4.17
Barnabas, 2002	Obstetrician counselling and testing salary for screening	19.00	26.38

Barnabas, 2002	Obstetrician counselling and testing salary for treatment	12.00	16.66
**Long-term care cost**			
Scott, 1998	Infant treated for HSV (include drug and culture)	1,470.00	2,218.70
Scott, 1998	Neonatal care if using caesarean delivery	821.00	1,239.15
Scott, 1998	Neonatal care if using vaginal delivery	480.00	724.47
Randolph, 1996	Neonatal herpes acute hospital care	10,160.00	15,334.69
Thung, 2005	Acute and long term care for normal/mild deficit	13,202.00	17,304.61
Randolph, 1996	Long term medical cost for moderate disability (Y1-Y65)	85,000.00	128,292.20
Thung, 2005	Acute and long term care for moderate deficit	134,202.00	175,906.12
Little, 2005	Lifetime cost and care for moderately disabled child	349,753.00	432,263.77
Rouse, 2000	Lifetime cost and care for moderately disabled child 1999	48,519.00	68,894.21
Baker, 2004	Lifetime medical and institutionalised cost for neonatal herpes	92,350.00	121,048.35
Binkin, 1989	Lifetime cost and care for moderately disabled child	125,000.00	220,697.66
Fisman, 2003	Lifetime cost of neonatal HSV with moderate neurological sequel	97,000.00	13,7734.46
Randolph, 1996	Long term medical cost for severe disability (Y1-Y65)	255,000.00	384,876.59
Thung, 2005	Acute and long term care for severe deficit	325,602.00	426,784.88
Little, 2005	Lifetime cost and care for severely disabled child	1,049,260.00	1,296,792.56
Rouse, 2000	Lifetime cost and care for severely disabled child	163,879.00	232,698.82
Binkin, 1989	Lifetime cost and care for severely disabled child	250,000.00	441,395.33
Fisman, 2003	Lifetime cost of neonatal HSV with severe neurological sequel	291,000.00	413,203.38
Tuite, 2010	Lifetime cost of neonatal HSV	164,870.00	187,438.10
Fisman, 2003	Lifetime cost of neonatal HSV	110,000.0	156,193.72
Baker, 2004	Counselling cost nurse (15 mins)	5.98	7.84
Baker, 2004	Counselling cost physician (5 mins)	6.67	8.74
Baker, 2004	Labour cost and supplies	15.58	20.42
Baker, 2004	Total cost without screening program	1,181.35	1,548.46
Baker, 2004	Total cost with screening for women	1,211.95	1,588.57
Baker, 2004	Total cost with screening for women and partner	1,267.24	1,661.04

Barnabas, 2002	Maternal mortality cost	443,858.00	616,230.57
Thung, 2005	Mortality cost	13,202.00	17,304.61
Barnabas, 2002	Neonatal care after caesarean	885.00	1228.69
Barnabas, 2002	Medical services for care of neonatal herpes	273,712.00	380,008.25
Barnabas, 2002	Long term care for neonatal herpes	140,766.00	195,432.58
Barnabas, 2002	Caregiver cost for neonates due to neonatal herpes	149,943.00	208,173.47

Figure 1: Methodological quality of included economic studies using CHEC Checklist

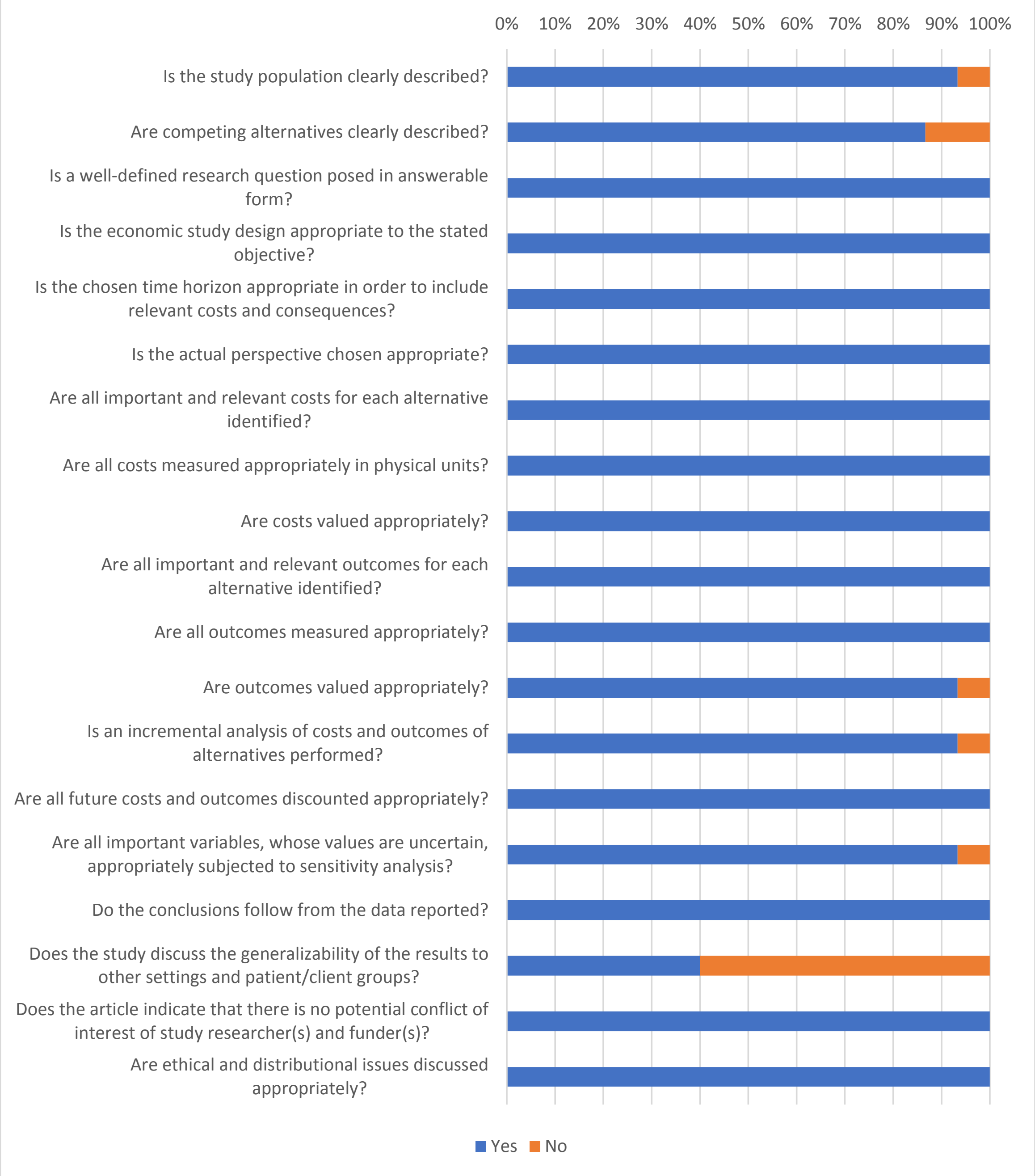

Figure 2: Methodological quality of included costing studies using Larg and Moss Checklist

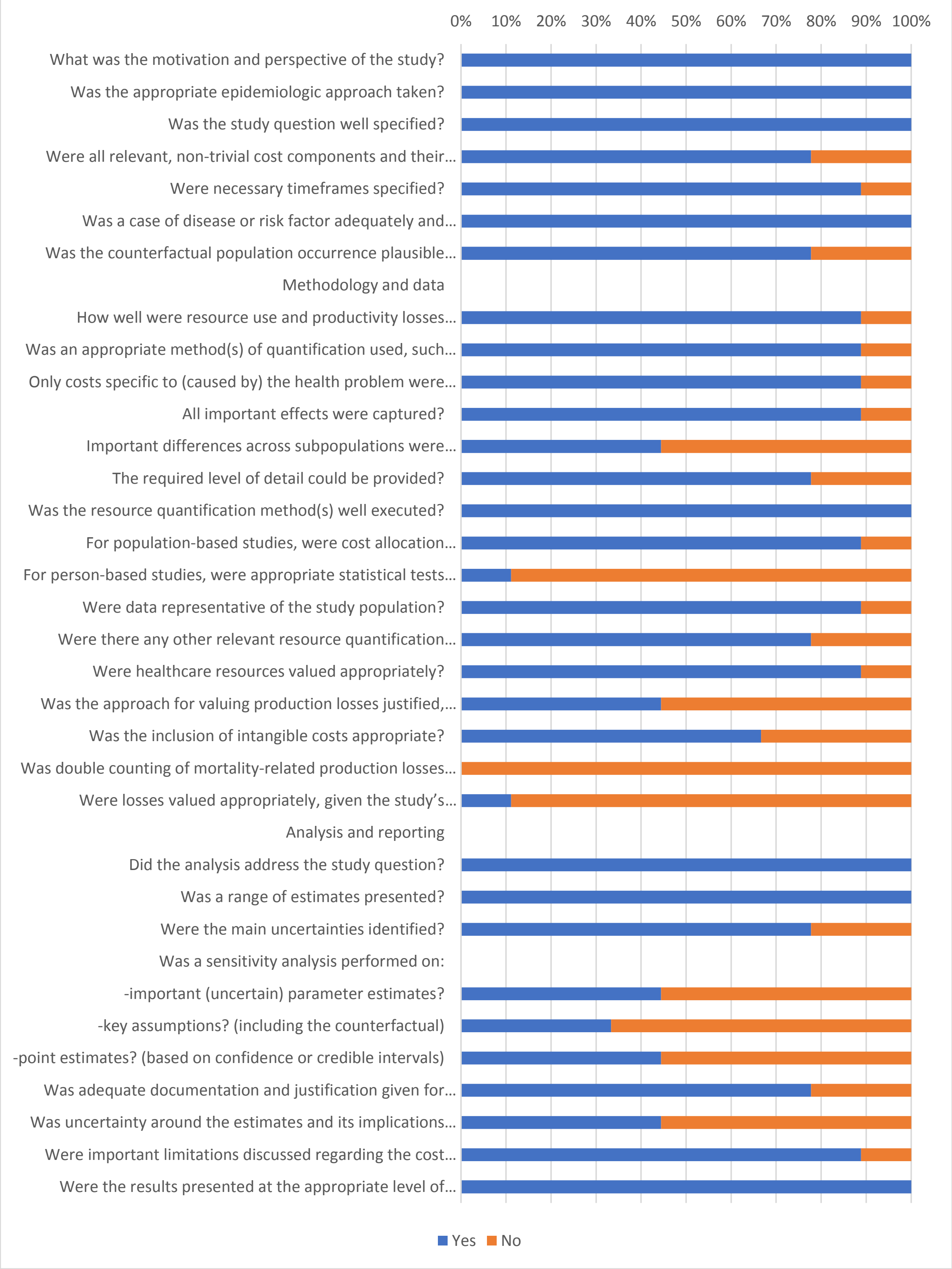

Supplement: Supplementary data [file bmjopen-2021-049618supp001.pdf]
