# Supplementary material for: Healthcare resource utilisation pattern and costs associated with herpes simplex virus diagnosis and management: a systematic review
Source: BMJ Open. 2022 Jan 4;12(1):e049618. doi: 10.1136/bmjopen-2021-049618 (PMC8728455; doi:10.1136/bmjopen-2021-049618)
Supplement: Author's manuscript [file bmjopen-2021-049618.draft_revisions.pdf]

BMJ Open

BMJ Open is committed to open peer review. As part of this commitment we make the peer review history of every article we publish publicly available.

When an article is published we post the peer reviewers' comments and the authors' responses online. We also post the versions of the paper that were used during peer review. These are the versions that the peer review comments apply to.

The versions of the paper that follow are the versions that were submitted during the peer review process. They are not the versions of record or the final published versions. They should not be cited or distributed as the published version of this manuscript.

BMJ Open is an open access journal and the full, final, typeset and author-corrected version of record of the manuscript is available on our site with no access controls, subscription charges or pay-per-view fees (http://bmjopen.bmj.com).

If you have any questions on BMJ Open's open peer review process please email info.bmjopen@bmj.com

BMJ Open

Health resource utilization pattern and costs associated with herpes simplex virus diagnosis and management : a global systematic review

Journal:	*BMJ Open*
Manuscript ID	bmjopen-2021-049618
Article Type:	Original research
Date Submitted by the Author:	28-Jan-2021
Complete List of Authors:	Lee, Shaun Wen Huey ; Monash University - Malaysia Campus, School of Pharmacy<br>Gottlieb, Sami L.; World Health Organization, Department of Reproductive Health and Research<br>Chaiyakunapruk, Nathorn; University of Utah Department of Pharmaceutics
Keywords:	HEALTH ECONOMICS, Health policy < HEALTH SERVICES ADMINISTRATION & MANAGEMENT, Public health < INFECTIOUS DISEASES

SCHOLARONE™
Manuscripts

I, the Submitting Author has the right to grant and does grant on behalf of all authors of the Work (as defined in the below author licence), an exclusive licence and/or a non-exclusive licence for contributions from authors who are: i) UK Crown employees; ii) where BMJ has agreed a CC-BY licence shall apply, and/or iii) in accordance with the terms applicable for US Federal Government officers or employees acting as part of their official duties; on a worldwide, perpetual, irrevocable, royalty-free basis to BMJ Publishing Group Ltd ("BMJ") its licensees and where the relevant Journal is co-owned by BMJ to the co-owners of the Journal, to publish the Work in this journal and any other BMJ products and to exploit all rights, as set out in our licence.

The Submitting Author accepts and understands that any supply made under these terms is made by BMJ to the Submitting Author unless you are acting as an employee on behalf of your employer or a postgraduate student of an affiliated institution which is paying any applicable article publishing charge ("APC") for Open Access articles. Where the Submitting Author wishes to make the Work available on an Open Access basis (and intends to pay the relevant APC), the terms of reuse of such Open Access shall be governed by a Creative Commons licence – details of these licences and which Creative Commons licence will apply to this Work are set out in our licence referred to above.

Other than as permitted in any relevant BMJ Author's Self Archiving Policies, I confirm this Work has not been accepted for publication elsewhere, is not being considered for publication elsewhere and does not duplicate material already published. I confirm all authors consent to publication of this Work and authorise the granting of this licence.

For peer review only

Health resource utilization pattern and costs associated with herpes simplex virus diagnosis and management : a global systematic review

Shaun Wen Huey Lee[1,2] , Sami L. Gottlieb[3], Nathorn Chaiyakunapruk[1,4]

[1] School of Pharmacy, Monash University Malaysia, Jalan Lagoon Selatan, 47500 Selangor, Malaysia

[2] School of Pharmacy, Taylor's University, Jalan Taylors, 47500 Selangor, Malaysia

[3] Department of Sexual and Reproductive Health and Research, World Health Organization, Geneva, Switzerland

[4]Department of Pharmacy, College of Pharmacy, University of Utah, Salt Lake City, Utah, USA

Corresponding author:

Nathorn Chaiyakunapruk
Department of Pharmacotherapy
University of Utah College of Pharmacy
30 South 2000 East, Room 4964
Salt Lake City, UT 84112
Office: 801.585.3092
nathorn.chaiyakunapruk@utah.edu

For peer review only

Financial Support

This work was funded by the WHO Department of Sexual and Reproductive Health and Research, via support from the UNDP-UNFPA-UNICEF-WHO-World Bank Special Programme of Research, Development and Research Training in Human Reproduction (HRP) and the U.S. National Institute of Allergy and Infectious Diseases, part of the National Institutes of Health (U01 AI108543).

ABSTRACT

Objectives: The World Health Organization (WHO) highlights the need for a vaccine against herpes simplex virus (HSV) partly due to a high disease burden globally. Little is known about its economic burden across countries. This article aims to summarize existing evidence on estimates of costs and resource utilization associated with genital and neonatal HSV and its methodological variation in healthcare systems.

Methods: We searched seven databases without language restriction. Studies reported either resource utilization or cost associated with HSV-related healthcare, including screening, diagnosis and treatment of genital HSV infection and neonatal herpes prevention and treatment. Studies published from inception to August, 31st 2020 were included. A focused search was also performed to supplement the results. Data were extracted and summarized descriptively.

Results: Out of 11,443 articles, 38 were included. Most studies (35/38, 94.6%) were conducted in high income countries, primarily the United States, and were more often related to the prevention or management of neonatal herpes (n=21) than HSV genital ulcer disease (n=17). Most analyses were conducted before 2010. There was substantial heterogeneity in the reporting of HSV-related healthcare resource utilzation and associated costs. Economic burden estimates based on these costs were similarly heterogeneous, with wide variation in methodology, assumptions, and outcome measures between studies. For example, lifetime costs of neonatal herpes ranged from USD$48,519 to as much as USD$1,296,792, depending on whether long-term disability care costs were included.

Conclusions: A paucity of evidence exists on health resource utilization and costs associated with HSV infection, especially among low- and middle-income countries. Future research is needed on costs and healthcare utilization patterns to improve overall understanding of the global economic burden of HSV.

(271/300 words)

Keywords: herpes simplex virus; healthcare resource utilzation; neonatal herpes; pregnancy; genital ulcer

For peer review only

Strengths and limitations of this study

- Herpes simplex virus infections are common and can cause genital ulcer disease, neonatal herpes, increased HIV risk, and psychosocial consequences, but current prevention interventions are limited.
- This systematic review demonstrated that there were only limited studies describing economic burden of HSV.
- Most studies were conducted in high income countries related to the prevention and management of neonatal herpes compared to genital ulcer disease.
- Results from this study form a repository to inform future economic evaluations, which will be crucial to determine the potential value of interventions for HSV, such as vaccines
- Further research on the healthcare resource utilization patterns and cost of HSV is needed, especially from other low-middle income countries.

For peer review only

INTRODUCTION

Herpes simplex virus (HSV)-1 and HSV-2 are DNA viruses that belong to Alphaherpesviridae, a subfamily of the Herpesviridae family.[1] Both viruses can cause genital infection, which can have a profound impact on sexual and reproductive health. HSV-2 is almost entirely transmitted during sexual activity and is the most common cause of genital herpes, affecting more than one in every 8 individuals, or 491.5 million people, aged 15-49 years in 2016.[2] HSV-1 is the main cause of oral herpes but can also be transmitted to the genital area through oral sex. HSV-1 affects an estimated 3.7 billion people under age 50 globally, of which over 120 million may have genital infection.[2] While the prevalence of HSV infection is high globally, it varies widely by region, with the highest prevalence of both HSV-1 and HSV-2 in the African region, which is primarily comprised of low- and middle-income countries (LMIC).[1 2]

Genital HSV infection is lifelong and characterised by periodic reactivation. Many infections are asymptomatic or unrecognized, but up to a third of people may develop painful, recurrent genital sores known collectively as genital ulcer disease (GUD).[3] Antiviral medications can be taken episodically to shorten GUD outbreaks or taken daily (suppressive therapy) to reduce the number of outbreaks, but they are not curative. Pregnant women with genital HSV infection can also transmit the virus to their infants in the peripartum period, resulting in neonatal herpes.[4] Although this occurs only rarely, neonatal herpes has a high fatality and disability rate among surviving infants. As such, particularly in high-income countries (HIC), prevention measures such as caesarean section are often undertaken if a mother has active HSV lesions at delivery. Genital HSV-2 infection has also been linked to an increased risk of acquisition and transmission of HIV infection.[5]

The World Health Organization (WHO) has highlighted the need for a vaccine against HSV-2, due to large numbers of infections globally and the resulting disease consequences including GUD, neonatal herpes, and increased risk of HIV acquisition.[6] Multiple vaccine candidates have been studied to date with modelling studies showing that prevention of HSV-2 infection with a vaccine could potentially also reduce the incidence of HIV infection.[7] Vaccines targeting HSV-2 might also have benefits against HSV-1.[8] Understanding the potential value of HSV vaccines requires not only predicting the impact of the vaccines on HSV-related disease burden, but also on its economic burden. However, little is known about the economic burden of HSV globally. As a first step in estimating HSV-related

For peer review only

economic burden, we conducted a broad systematic review with the aim of summarizing all available evidence on costs and resource utilization associated with diagnosing, treating, and managing HSV infection and disease, and specific cost drivers across healthcare systems.

For peer review only

METHODS

Data Sources and Search Strategy

We electronically searched for relevant articles published from database inception to August 31st 2020 in 7 databases: PubMed, PsychINFO, EMBASE, Centre for Review and Dissemination, EconLit, CEA registry and WHO Library Database (WHOLIS). The search strategy was based on a broad combined search string "Herpes Simplex Virus" AND "cost" OR "resource utilization" OR "econ*", with no language restriction. A complete search strategy is detailed in Appendix 1. In addition, bibliographies of relevant articles were examined to identify potential studies not indexed in the aforementioned databases.

Study Selection

Studies were included if they were original articles that investigated resource utilization patterns and costs related to HSV infection including the cost of any diagnostic tools, consultation time, treatment and hospital cost related to detecting and managing all types of HSV-1 or HSV-2 related neonatal and genital infections and associated disease outcomes. We included articles which were published in any languages. A focused supplemental search was performed using the keywords listed in Appendix 2 based upon the inclusion above.

Data Extraction and Quality Assessment

The study followed a 2-stage process, where two independent reviewers screened the titles and abstracts for relevant studies, before the full texts were screened by another two independent reviewers for eligibility. Relevant information from the identified studies was extracted independently by two reviewers using a standardized data extraction sheet. At all stages, any disagreement was resolved by discussion between reviewers through consensus. Information collected from the data extraction sheet included: 1) general study information including country of the study, 2) HSV subtype and disease, 3) study design, 4) healthcare resource utilization, 5) costs of relevant tests, clinical care, hospitalisation, and medications, and 6) summary estimates of HSV-related economic burden. Methodological quality of all included economic studies was assessed using the Consensus Health Economic Criteria (CHEC) list. This checklist has been recommended for critically appraising published economic evaluations. The checklist has 19 domains and includes reporting standards for economic model characteristics (population, time horizon, perspective and

For peer review only

discount rate), identification and valuation of costs and outcomes, discussion points, conclusions as well as funding and conflicts of interest. All cost of illness studies were evaluated for risk of bias using the Larg and Moss's checklist. No quality appraisal was performed on studies reporting healthcare resource utilzation.

Data Analysis

A component-based analysis was used to describe and synthesise the overall findings from all included studies. Specifically, tabulation methods were used to report on study characteristics, outcomes and costs. Tables for resource utilization and disaggregated costs were presented and summarized. All costs were presented according to the recommendations of Turner et al., 2019[9]. For studies that did not provide the year of cost data, the year of publication was used. Adjustment for inflation was done using the Gross Domestic Product deflator (GDP deflator) of the studied country. Cost estimates were then converted and reported in 2017 United States Dollars (USD). GDP deflator and exchange rates were obtained from the World Bank.[10]

Patient and public involvement

Patients were not involved in this systematic review. Their input was not sought in the design, interpretation or writing of the document.

For peer review only

RESULTS

Study Selection

Our search yielded a total of 11,443 articles of which 8,779 articles were excluded as they were not relevant for this review based on title screening. The remaining 2,664 articles were further screened by title and abstract and 299 articles were assessed for inclusion. We excluded 261 articles (n= 98 for not related to HSV, n =44 review articles/case report, n =116 not reporting resource utilization or cost, n =3 available only in abstract), leaving a total of 38 studies included in this review, as shown in Figure 1.

Overview of Study Characteristics

Of the 38 included articles, 14 studies[11-24] described resource utilzation only, 12 studies[25-36] reported on costs, and 12 studies[37-48] reported both resource utilization and costs of HSV diagnosis/management. These studies, published from 1989 to 2020, reported resource utilzation or costs related to the diagnosis and management of HSV-related GUD among adults/adolescents[14-18 24 26-30 33-36 40 48] (n=17), neonatal herpes prevention in pregnant mothers (n=13)[19-21 23 25 31 32 38 39 42-45] and neonatal herpes management[11-13 22 37 41 46 47] (n=8). The majority of studies were conducted in HIC (35/38, 94.6%) including the United States[11 13 16 18 21 23 25 26 30 31 34-48] (n= 26), Canada[14 15 22 32] (n=4), United Kingdom[19 29] (n=2), France[12 24] (n=2) and Ireland[20] (n=1)), while only one study (1/38, 2.6%) was conducted in a middle-income country, in particular South Africa[28]. A global survey focusing on the experiences of patients receiving care for genital herpes in 78 countries included some data on healthcare utilization.[17] In addition, a modelling study estimated the costs of implementing the Global Health Sector Strategy on Sexually Transmitted Infections (STIs), 2016-2021, in 117 LMICs, including costs related to syndromic management of GUD, the vast majority of which is caused by HSV-2.[33]

Methodological Heterogeneity

There was substantial heterogeneity in the reporting of the included studies. Most studies were cost or resource utilization studies (n=23), while the remaining were cost-effectiveness studies (n=15). Among cost or resource utilization studies, data were collected retrospectively (n=13), prospectively (n=7), or not reported (n=7). The number of participants in each study varied, which could be as few as 39 participants to as large as 42 million in studies that analysed claims datasets. Twenty-one studies

For peer review only

(21/38, 55.3%) included participants who had either HSV-1 or 2, ten studies (10/37, 27.0%) specifically included participants with HSV-2, while the remaining eight studies (8/38, 21.1%) did not specify which type of HSV they examined. A summary of the characteristics of these studies is presented in Table 1, and study findings are presented in Tables 2 and 3 (See appendix for detailed unit cost tables and accompanying references).

Cost and health resource utilization pattern of genital herpes infection

Among all 17 studies[14-18 24 26-30 33-36 40 48] investigating cost and health resource utilization pattern of genital herpes, 11 studies reported some cost components of care for genital herpes infection[26-30 33-36 40 48] (Tables 1 and 2). All but one of these studies were conducted in HIC and only one LMIC study (from South Africa) was found. The cost components of the included studies were variably reported. Three studies[27 30 48] reported laboratory testing costs associated with diagnosing HSV. Eight studies[26 27 29 30 33 36 40 48] described costs associated with syndromic management of GUD. In four studies[28 29 33 48], the authors describe the drug charges associated with treatment or prevention of HSV using oral acyclovir (doses of 200mg-400mg). The cost reported varied considerably, ranging between USD$0.53 to USD$16 for a 5 to 7 day treatment course for episodic GUD and USD$40 for a month of suppressive therapy with acyclovir. Two studies[27 40] provided the total drug charges associated with overall management of GUD, but no details related to the treatment regimen, duration or HSV of HSV being treated (Table 2). Seven studies[27-29 33 43 44 48] described labour and service delivery costs such as cost of physician visits, drug procurement cost, counselling cost and clinical examination associated with HSV. Similarly, there was variation in terms of reported labour and service delivery cost, which could be as low as USD$0.28 for 10-minute counselling[29] to as high as USD$120 for consultation and lost wages of patient time[48]. Indirect costs were considered only by Szucs *et al*, who estimated HSV-related productivity losses, which was estimated at USD$60 a visit[27].

Considering the cost components together, Owusu-Edusei *et al* estimated that the lifetime direct medical cost per case of genital HSV infection in the U.S. (considering only GUD-related costs and adjusted to 2017 USD) was USD$855 among men (range: USD$428- USD$1,284) and USD$698 among women (range: USD$350- USD$1,047)[26]. This translated to a total cost of USD$607.3 million (range: USD$303.59 million – USD$ 910.89 million in 2017 USD) for lifetime management of new or newly diagnosed cases of HSV-2 in the United States occurring in 2008. Scuzs *et al* meanwhile estimated that the annual direct and indirect medical costs in the United States would amount to

For peer review only - http://bmjopen.bmj.com/site/about/guidelines.xhtml

USD$983 million, based upon an estimated 3.1 million symptomatic genital HSV episodes (both new and recurrent) a year[27].

The only middle income country study, from South Africa[28], reported the diagnostic/ operational costs associated with medication, staff and laboratory costs for daily HSV-2 suppressive therapy among people living with HIV[28]. The median cost for HSV-2 suppressive therapy per life-year gained ranged between USD $685 to USD $951 (adjusted to 2017 dollar) among HIV-1 infected anti-retroviral naïve women. The authors estimated that this could be a cost-effective method for delaying HIV disease progression, especially when the price of acyclovir was lower than the price of USD $0.026/day for a twice daily 400mg dose. However, this study was conducted when ART use was recommended only when CD4 count fell below a threshold of <200 cells/µL or <350 cell/µL (Appendix Table 2). On a more global level, in Korenromp *et al*'s cost estimates for implementing the Global STI Strategy in 117 LMIC over 2016 to 2021, the authors reported that it would cost approximately USD$109 million to diagnose and treat HSV-related GUD episodes seen in clinical care, not including service delivery costs.[33] These costs were estimated despite assuming that only about 4% of all HSV-2 infected people would seek care for GUD (15% recognizing symptoms and 28% of those seeking care).

A total of 8 studies described health resource utilization patterns for genital herpes infection[14-18 27 36 40], and all were from high income countries (Tables 1 and 3). Five of these studies[14 16-18 36] reported the population rate of seeking medical care for HSV, based upon retrospective analyses of databases of patients from health surveys[16-18]. In the study by Xia and colleagues, the authors found that the total genital herpes associated ED use have increased from 24,747 visits in 2006 to 36,518 in 2013[36]. It is important to note that none of the studies reported the proportion of those seeking medical care among HSV-infected individuals. Most of these consultations were relatively short in nature, and were less than 15 minutes (79%)[17]. Two studies described the diagnostic methods used to determine HSV among their population. In the first study conducted in 2004, Patrick *et al.* surveyed physicians in 78 countries and reported that the most commonly used test was viral culture, which was performed in 49% of the individuals[17] (Table 3). At the time of the study, the use of PCR was not yet common in clinical practice. A recent study in France by Heggarty *et al.* in 2020 found that PCR is now more commonly used, with 43.3% of respondents in their survey stated that they would conduct PCR in addition to HSV serology while another 39.9% would conduct PCR only to confirm a HSV diagnosis[24].

For peer review only

Treatment patterns of individuals with genital herpes were also reported in four studies[15 17 24 40]. The study by DesHarnais *et al* in 1996 reported on antiviral use only among hospitalized patients with herpes infections, which is unlikely to be representative of the vast majority of people with HSV infection. Patrick *et al* in their survey found that 65% of people with genital herpes had ever been treated with antivirals, while 18% used topical prescription medication and 13% used over the counter topical cream. Among these individuals, 67% had received episodic therapy while 31% received chronic suppressive therapy (Table 2). Another study on herpes-related quality of life reported that 76.9% of respondents had ever been treated with antivirals, and 33.3% of the respondents with HSV were on suppressive antiviral therapy when the survey was administered[15].

Cost and health resource utilization pattern of prevention of neonatal herpes among pregnant mothers

Nine studies reported costs for neonatal herpes prevention among pregnant mothers[25 31 32 38 39 42-45] (Tables 1 and 2). Seven studies[31 32 38 39 42 43 45] provided estimates on the cost for treatment and childbirth delivery options, including caesarean and vaginal delivery in addition to inpatient costs. The cost of hospitalisation ranged considerably, and could be as low as USD$300 to as high as USD$32,483, while the cost of delivery ranged between USD$2,300 -$9,490. The costs associated with different laboratory tests used, such as ELISA screening or viral cultures[32 39] were reported, while detailed listing of the cost component of different delivery methods and hospital care were included in some studies (Appendix Table 3). The cost-effectiveness studies examined the impact of either acyclovir suppressive therapy[25 31 42 43] or routine antenatal screening[32 38 39 44 45] for prevention of neonatal herpes. In a study by Randolph *et al* in 1996[43], the authors found that prophylaxis with acyclovir during late pregnancy could be a cost-effective strategy to reduce the need for caesarean delivery due to genital herpes outbreaks during labour. Baker and colleagues in 2004 further expanded this work and estimated that adding serological testing to antiviral suppressive therapy had an incremental cost per quality-adjusted life year gained (QALY) of $18,680, compared with no screening or suppressive therapy[38]. A modelling study by Tuite *et al* in 2010 had similar findings related to screening for HSV in pregnancy[32].

For peer review only - http://bmjopen.bmj.com/site/about/guidelines.xhtml

Our focused search found a total of 10 studies which reported resource utilzation among pregnant mothers to prevent neonatal herpes[19-24 38 39 42 44]. Among these, four were cost-effectiveness studies which had provided some information regarding resource utilzation based upon estimates from literature or assumptions.[38 39 42 44] In one of the earliest studies by Brocklehurst in 1995, a survey of British obstetrician-gynaecologists revealed that most would recommend some form of antenatal screening for HSV using viral cultures usually by week 34 of gestation[19]. However, such screening is no longer recommended in the UK. Studies within HICs that have national obstetrics guidelines recommending caesarean delivery when HSV lesions are present at delivery have shown that most clinicians follow this guidance[20-23]. For example, in a Canadian study, caesarean section was offered "most of the time" to women with HSV lesions at delivery by 92% of obstetricians and 82% of family physicians[22]. In addition, in these settings women with genital herpes are often offered antiviral suppressive therapy in the third trimester[20 22]. Both valacyclovir and acyclovir have been used, with difference in preference by country. In the most recent survey of clinicians managing pregnant women with HSV by Heggarty *et al* in 2020, the authors noted that 68.4% "always" prescribe suppressive antiviral therapy during the third trimester and an additional 11.6% "often" prescribe it for women with symptomatic primary HSV infection during pregnancy.[25] For women with recurrent symptoms during pregnancy, 55.1% of providers "always" prescribe and 12.9% "often" prescribe antiviral prophylaxis in the third trimester.[24]

Cost and health resource utilization pattern of neonatal herpes management

Four studies[37 41 46 47] reported cost of neonatal herpes management and reported only direct medical costs (Tables 1 and 2). One study reported direct non-medical cost for long-term care of individuals with neurological disability due to sequelae of HSV[39]. All studies were in HIC. The reported cost of hospitalisation of neonatal HSV ranged considerably, from S27,843 to $92,664. One study reported the cost associated with hospital readmission, which was reportedly similar to the first hospitalisation episode[46]. Six studies[32 42-45 48] accounted for the costs of informal care in their calculation. Informal caregiving was defined as care provided by caregivers for infants who had neurological sequelae following neonatal herpes. In total, seven studies[32 39 42-45 48] estimated long-term care costs of neonatal herpes patients. One of these, by Thung *et al*[45], provided the estimated cost for long term care of neonates with mild neurological deficit due to HSV, which cost USD$17,304.61 after adjusting for inflation to 2017 values. Six studies[39 42-45 48] provided estimates for the lifetime cost of caring for a child with moderate and severe disability, and fall within the range USD$68,894 to USD$432,263 and USD$232,698 to USD$ 1,296,792 respectively. It is important to

For peer review only

note that all studies relied on estimation of long-term costs calculated by Weitzman[49] with some different assumptions, while one study[39] used other sources of data.

A total of 7 studies[11-13 37 41 46 47] described resource utilization among individuals with neonatal herpes (Tables 1 and 3). These studies described the length of stay for hospitalization which varied considerably, with median hospital stays ranging from 6-34 days[11 12]. Ahmad *et al* noted that nearly 9.4 to 9.8% of neonates who had HSV required ICU stay[11]. None of the studies reported the number of days for ICU hospitalization.

For peer review only

DISCUSSION

Our review revealed a heterogeneous body of evidence on the health resource utilization and costs associated with genital and neonatal HSV infection, as well as some summary economic estimates and cost-effectiveness studies of HSV intervention strategies, such as use of antivirals or screening, which included unit cost data. While the evidence base provides a starting point for understanding, several gaps remain. Despite the broad search strategy and inclusion criteria, we identified only 38 papers, which shows the paucity of data on HSV-related healthcare resource utilzation as well as economic costs, especially from LMIC settings. The lack of data from LMIC is particularly concerning, as these countries bear the greatest burden of HSV infection and disease.[2 3 50] The current review only identified one cost-effectiveness analysis from a middle income country[28] focused on people living with HIV only, and one high-level modelling study predicting costs of implementing care for HSV GUD across 117 LMIC globally[33]. In addition, many of the studies we found were relatively old and may not reflect current practices such as the use of newer diagnostics (e.g. PCR) and newer care recommendations.

While data on resource utilzation and costing were most comprehensive from the US, large gaps remain in many areas. For example, Gilbert and colleagues[16] described the proportions of individuals seeking care for genital herpes among adults aged 18 to 24 from 2000 to 2006, but since then there have been no new updates. In terms of costing, we noticed similar trends, as studies[26] mostly referenced cost data collected in 2001 by Szucs *et al*[27]. This lack of data is similarly noted related to HSV infection during pregnancy. While some information from health surveys exists, healthcare resource utilzation information is rarely tracked or reported. Our search demonstrated that for most of the world, data on HSV related resource utilzation are sparse. As such, new data sources and better data collection efforts are needed to collect these standardized non-fatal data from diverse healthcare settings. One major need is an understanding of how closely clinicians follow national guidelines on HSV care and treatment, such as the studies by Kenny *et al*[22] and Heggarty *et*[24] *al* from Canada and France respectively.

Our review was also constrained in summarizing findings across studies or countries and in conducting across-study comparisons, due to the limited data and differing methodologies, healthcare settings, and practices, particularly for healthcare resource utilzation. Another concern was the heterogeneity in data presentation in many studies identified. For example, the length of

For peer review only

hospital stay reported in studies varied considerably, with different assumptions used by authors, and as a result, the cost of hospitalisation varied significantly even within the United States, which limits the potential generalizability of these findings across different settings[12 37 41 47]. Healthcare practices also differ between LMIC and HIC with respect to how HSV is managed, eg, most HSV cases in LMICs are treated as part of syndromic management for GUD, without diagnostic testing. This may mean that additional testing costs might need to be considered for HICs, whereas additional treatment, for example for syphilis and chancroid, which can also cause GUD syndromes, might need to be considered for LMICs. The focus on GUD more generally in LMICs may have made it more challenging to identify potentially relevant HSV-specific studies for LMIC settings.

In order to estimate the global economic burden of HSV to contribute to the understanding of the potential value of HSV interventions, research on HSV-related costs and healthcare utilization patterns is urgently needed, especially from LMIC settings. Standardization of methods for the measurement and reporting of economic costs would enhance across-study comparisons and inform prioritization strategies of global funders. Only one study broadly attempted to quantify the economic burden of HSV, which the authors estimated would require a projected investment of around USD$109 million from 2016 to 2021, just for the management of HSV-associated GUD, not considering service delivery costs[33]. However, this analysis only modelled treatment of HSV GUD for a small proportion of people with HSV-2 infection (approximately 4%: assuming 15% would recognize symptoms and 28% of those would seek care) and did not account for HSV recurrences within a given year. New global estimates of HSV GUD suggest this is likely an underestimate.[3] In addition, as this model lacked country-level estimates of baseline disease and did not take into account the full spectrum of disease outcomes related to HSV nor the burden on health systems, the costing estimates remain imprecise and incomplete, suggesting the need for a more comprehensive model.

This is the first systematic review of scientific literature on the healthcare resource utilzation for HSV. We conducted a comprehensive literature search and included grey literature through our focused search. Nevertheless, most studies were only conducted in HIC especially from the USA. We did not find any study that originated from the Asia region. As the practice and thus utilzation of resources will vary between setting and countries due to epidemiological and health systems, this will limit the generalisability of findings. We assessed study quality of all included studies, which allows for readers to assess the internal validity of these studies. The literature search was also

For peer review only

limited to English language. As data on healthcare resource utilzation may be published in government reports, or book chapters, these may not have been retrieved and included into this review.

For peer review only

CONCLUSION

This review is the first attempt and a key step towards providing data needed to understand the global economic burden of HSV infection, for both HICs and LMICs. Available economic estimates, primarily from HICs, suggest the economic burden of HSV infection could be substantial. However, the global picture remains incomplete. Results obtained from this study will form a repository which can inform future economic evaluations of interventions for HSV infection, including HSV vaccines, microbicides, or new antiviral medications.[51] These types of economic data are crucial not only to improve the planning and development of any future HSV-related healthcare interventions, but also to optimize the allocation of healthcare expenditures and medical resources.

For peer review only

Contributors

SWHL served as the lead author, conducted the research, conducted the analyses, integrated the input from all team members and drafted the initial manuscript. SG directed the initial research and contributed to the initial draft, integrated her view points and served as an expert in this work.. NC conducted the research, mediated the discussion and helped refine the draft. All authors approved the final manuscript.

Data sharing statement

All relevant information and data have been presented in this article and its accompanying supplement.

For peer review only

Table 1: Summary of included studies reporting healthcare costs and/or resource utilzation related to HSV infection

Author, year Country	Population and setting	Study design	Study objective	Sample size	HSV-subtype		Cost data		Healthcare resource utilzation	
					1	2	Healthcare delivery process	Treatment	Healthcare delivery process	Treatment
**HSV genital ulcer disease among adults/adolescents**										
Almonte-Vega, 2020 USA[35]	General population aged 15-49 years old	Cost-analysis	To study the dynamics of HSV–2 transmission, control and impact of treatment policies	-		x		x		
Aslam, 2012 Canada[14]	Records of individuals in the Canadian Disease and Therapeutic Index (CDTI)	Retrospective study	To investigate the rates of diagnosed cases of GH in Canada from 2002 to 2007	652					x	
Desharnais, 1996 USA[40]	Adults with herpes diagnosis from the HCIA Clinical Pathways Data Base	Retrospective study	To describe patterns of antiviral drug use for patients hospitalized with chickenpox, herpes simplex, and herpes zoster infections, and also for a subgroup of herpes patients with severe infections (systemic infections, eye infections, encephalitis, hemorrhagic pneumonitis, and other severe conditions)	3011	x	x		x		x
Fisman, 2002 USA[34]	Individuals aged 15 to 39 years	Cost-effectiveness	To project the future burden of HSV-2 infection in the	-		x	x	x		

For peer review only

			United States, using a mathematical model that incorporated epidemiologic trends documented between 1976 and 1994							
Fisman, 2003 USA[48]	Heterosexual couples	Modelling study	To evaluate the projected cost effectiveness of strategies to prevent HSV-2 transmission in couples with no history of HSV-2 infection	-		x		x		
Fisman, 2005 Canada[15]	Individuals with recurrent genital ulcer	Prospective study	To estimate the impact on health-related quality of life associated with both symptomatic and asymptomatic GH	39	x	x				x
Gilbert, 2010 USA[16]	Young adults	Retrospective study	To investigate characteristics associated with GH screening and diagnosis in sexually active young adults aged 18 to 24	Add Health Data: 11,570 NCHA: 222,740	x	x			x	
Korenromp, 2017[33]	People 15-49 year old living with HSV-2	Modelling study	To estimate the costs of reaching the 2020 STI strategy milestones for the period 2016–2021, to support policy, planning, implementation, and future cost-benefit evaluation of the global STI strategy 2016–2021.	-		x	x	x		
Owusu-Edusei, 2013a	People aged 15-25 years	Retrospective study	To examine the utilization and cost of the diagnostic methods used for STI screening among	-		x	x			

For peer review only

USA[30]			privately insured adolescent and young adult population							
Owusu-Edusei, 2013b USA[26]	-	Cost of illness analysis	To update the estimates of lifetime direct medical cost for 8 major STI	-				x		
Patrick, 2004 Worldwide survey from 78 countries[17]	Subjects with genital herpes	Survey	To describe patient experiences and views regarding genital herpes management	2075	x	x			x	x
Szucs, 2001 USA[27]	General population	Economic analysis	To estimate the economic burden of GH in the USA, using two different costing approaches	465,075			x	x		
Tao, 2000 USA[18]	General population	Cost-of-illness analysis	To assess the US direct medical expenditures for genital herpes and its complications to assist policy makers in allocating limited STD resources efficiently	-		x			x	
Vickerman, 2008 UK[29]	-	Cost-effectiveness	To compare the cost per ulcer treated of using the 1994 and 2003 algorithms amongst individuals presenting with GUD	-		x		x		
Vickerman, 2011 South Africa[28]	HIV+ women	Cost-effectiveness	To estimate the cost-effectiveness of daily acyclovir for delaying HIV-1 disease progression in women not eligible for antiretroviral therapy (ART)	300		x		x		

For peer review only

Xia, 2018 United States[36]	General population	Retrospective study	To determine the utilization and cost burden associated with HSV infection visits to U.S. EDs in recent years from 2006-2013	704,728			x		x	
**Neonatal herpes prevention among pregnant women**										
Baker, 2004 USA[38]	-	Cost-effectiveness	To determine whether serologic testing for herpes simplex virus type 2 (HSV-2) in pregnant women and their partners is cost-effective	100,000		x		x	x	x
Barnabas, 2002[25] USA	-	Cost-effectiveness	To assess the potential effectiveness, cost effectiveness, and benefit of suppressive therapy among herpes simplex virus serodiscordant sex partners during pregnancy		x	x	x	x		
Binkin, 1989 USA[39]	Pregnant women with HSV	Cost-effectiveness	To present a reanalysis of the cost effectiveness of maternal herpes screening and a review of the changes that have occurred in the screening recommendations since 1980	3,600,000	x	x	x	x	x	
Brocklehurst, 1995 UK[19]	All members and Fellows of the Royal College of Obstetricians and Gynaecologist resident	Survey	To determine the clinical practice among obstetricians in the antepartum and intrapartum management of women with recurrent genital herpes infection	2252	x	x			x	x
Brown, 2003 USA[23]	Pregnant women from university,	Cohort study	To determine the effects of viral shedding, maternal HSV	58362	x	x				x

For peer review only

	army and community hospitals		serological status and delivery route on risk of transmission of HSV from mother to infant							
Heggarty, 2020 France[24]	Healthcare providers for pregnant women	Survey	To evaluate health care provider knowledge, and collect information on management of genital herpes during pregnancy and infants born to mothers with herpes	354	x	x			x	x
Kenny, 2013 Canada[22]	Obstetrician, gynaecologist and family physicians offering maternity care practicing in Alberta	Survey	To identify the practice patterns of physicians providing prenatal care in Alberta with respect to prevention of neonatal HSV infection, including their prescribing of antiviral therapy to pregnant women in the third trimester.	183	x	x			x	x
Little, 2005 USA[42]	Women with a history of diagnosed genital HSV	Cost-effectiveness	To determine the clinical benefits and cost-effectiveness of prophylactic acyclovir in women with a history of HSV but no recurrence during pregnancy	-	x	x		x		x
Lynn, 2017 Ireland[20]	Pregnant women with genital HSV from a university hospital	Antenatal chart review	To describe the HSV management in pregnancy at a joint antenatal genital maternity hospital	107	x	x			x	x
Randolph, 1996 USA[43]	Antenatal women with recurrent genital HSV	Cost-effectiveness	To compare the cost-effectiveness of oral acyclovir prophylaxis in late pregnancy compared to caesarean delivery for genital herpes	10,000			x	x		

For peer review only

			lesions in the prevention of neonatal herpes transmission from mothers with recurrent genital infections							
Rouse, 2000 USA[44]	Antenatal women	Cost-effectiveness	To evaluate the potential cost effectiveness of herpes simplex virus antibody screening	8,538	x	x	x	x	x	
Scott, 1998 USA[31]	-	Cost-effectiveness	To determine whether acyclovir suppression provides a greater cost savings over no medical therapy in the management of recurrent genital herpes (HSV) in pregnancy	-	x	x	x	x		
Stankiewicz Karita, 2017 USA[21]	Pregnant women from a hospital	Retrospective study	To investigate the frequency of invasive obstetric procedures and caesarean deliveries for women with known HSV infection	449		x			x	
Thung, 2005 USA[45]	Married women	Cost-effectiveness	To determine the cost-effectiveness of routine antenatal screening for HSV-1 and HSV-2 in women without a known history of genital herpes.	100,000	x	x		x		
Tuite, 2010 Canada[32]	Pregnant women	Cost-effectiveness	To assess the effectiveness and cost effectiveness of identifying pregnant women at risk of de novo HSV acquisition to prevent vertical HSV transmission	100,000	x	x	x	x		

For peer review only

**Neonatal herpes management**										
Ahmad, 2015 USA[11]	Neonates who sought care in emergency department	Retrospective study	To evaluate whether guideline implementation affected the ED's decision to test for HSV, ED use of HSV polymerase chain reaction (PCR) and acyclovir	308	x	x				x
Ambroggio, 2009 USA[37]	Neonates with HSV and received intravenous acyclovir and discharge from Paediatric Health Information System	Retrospective study	To quantify the economic burden of neonatal HSV during initial hospitalization while focusing on factors, such as congenital anomalies and HSV-associated complications, which increase hospital charges and length of hospital stay among neonates with HSV	406	x	x		x		x
Bernard, 2013 France[12]	Patients aged 28 days and above from the French national hospital discharge database	Prospective study	To compare the data from the French national hospital discharge database (Programme de Me´ dicalisation des Syste` mes d'Information; PMSI) and from the prospective study conducted in 2007 and evaluate the reliability of PMSI as a tool to assess the trends of encephalitis in France	1,947	x	x				x
Donda, 2019 USA[41]	Neonates with ICD-9 codes for neonatal HSV in the National	Retrospective study	To examine the temporal trends in the incidence and outcomes of neonatal HSV in the United States	42,726, 336				x		x

For peer review only - http://bmjopen.bmj.com/site/about/guidelines.xhtml

	Inpatient Sample from 2003-2014									
Flagg, 2011 USA[47]	Inpatient records of infants aged 60 days or younger from the Healthcare Cost and Utilization Project Kids' Inpatient Database	Retrospective study	To estimate the incidence of HSV infections for the United States during 2006, as well as demographic-specific rates, by using nationally and regionally weighted estimates from a population-based sample of inpatient data	4,106,488	x	x		x		x
Mahant, 2019 USA[46]	Records of neonates from the Medicaid claims database from 2009 - 2015	Retrospective study	To examine the incidence, mortality, and health care use related to neonatal herpes HSV infection.	2,107,124				x		x
Owusu-Edusei, 2015 USA[13]	Insurance claim data on inpatient admission from the Truven Health Analytics MarketScan Commercial Claims and Encounters Database	Cost-of-illness analysis	To estimate the average excess inpatient cost of neonatal herpes simplex virus (NHSV) infection from 2005 to 2009 insurance claims data	474,743	x	x				x

For peer review only

Table 2: Detailed description of studies reporting cost (unit cost)

Author, year Country	Population and setting	Diagnostic costs (range)	Treatment costs* in original year of value (range)	Hospitalisation costs (range)	Other healthcare delivery costs (range)	Lifetime management cost (range)
**Genital ulcer disease among adults/adolescents**						
Almonte-Vega, 2020 USA[35]	General population aged 15-49 years old	Microbiological lab test (unspecified): $80.17	Acyclovir treatment (duration not specified): $86.33	NR	Consultation, clinical examination and diagnostic: $161.85	NR
Desharnais, 1996[40]	Adults with herpes diagnosis identified from the HCIA database	NR	Total drug charges: $1941<br>Antiviral drug charges (not specified): $1070	Hospital charges: $5637	NR	NR
Fisman, 2002[34]	Individuals aged 15 to 39 years	NR	Cost of treatment for primary syndrome<br>Male: $470 ($370-5$60)<br>Female: $830 ($670-$1000)<br><br>Antiviral therapy<br>Relapse: $17 ($9-$36)<br>Monthly suppressive therapy: $40 ($20-$220)	NR	Clinic visit: $120 ($90-$150)<br>Obstetrical care: $310 ($130-$800)	Initial cost of caring for neonates with HSV: $42,600<br>Lifetime medical and long-term care cost for infants with moderate neurological sequalae: $97,000<br>Lifetime medical and long-term care cost for infants with severe neurological sequalae: $291,000

For peer review only

Fisman, 2003[48]	Heterosexual couples	Western blot: $60 ($45-$90)<br>ELISA: $5 ($3-$35)	Cost of treatment for primary syndrome<br>Male: $450 ($360-5$40)<br>Female: $800 ($640-$960)<br><br>Acyclovir (per episode): $16 ($9-$35)<br>Acyclovir (monthly suppressive): $40 ($20-$215)	NR	Clinic visit: $120 ($90-$145)<br>Labour: $120 ($90-$145)	Lifetime cost of care of neonatal HSV-2: $110,000 ($85,000-$860,000)
Korenromp, 2017[33]	People 15-49 year old living with HSV-2	NR	Acyclovir 400mg per tab: $0.04	NR	Treatment service delivery (not specified): $10<br>Procurement cost: $0.21	NR
Owusu-Edusei, 2013a[30]	People aged 15-25 years	Laboratory test (unspecified): $24.30-27.05	NR	NR	NR	NR
Owusu-Edusei, 2013b[26]	-	NR	NR	NR	NR	Lifetime medical cost per case, median(range):<br>Men: $761 (381-1,142)<br>Women: $621(311 - 932)<br>Lifetime cost of new infections acquired in 2008: $435.9 million

For peer review only

Szucs, 2001[27]	General population	Laboratory test: $1.5-76.50	Drug: $64-131	Hospitalisation: $669	Labour: $39.8 -62.6<br>Clinic visit: $36.20-73<br>Day off work: $144	NR
Vickerman, 2008[29]	-	NR	Acyclovir 200mg tds for 5 days: $0.53- 5.24	NR	Counselling cost: $0.28	NR
Vickerman, 2011[28]	HIV+ women	NR	Acyclovir 400mg: $0.07<br>Yearly ART cost: $1700 (1359-2000)	NR	Staff costs/women 3m treatment cycle: $15.60	NR
Xia, 2018[36]	General population	NR	NR	ED: $1,069		
**Neonatal herpes prevention among pregnant mothers**						
Baker, 2004[38]	-	Laboratory test with labor cost for HSV-2: 15.58 – 60.00	Average antiviral daily cost (assuming 50% on generic acyclovir 400mg tds and 50% on valacylovir qd): $1.70-7.90<br>Acyclovir 400mg: $0.366- 1.955<br>Valacyclovir 500mg/tab: $3.95<br>Valacyclovir 1g/tab: $6.49	Delivery: $4,779-22,838	Labour cost: $15.58 – $60<br>Counselling cost: $5.98-$6.67	Lifetime cost of care of neonatal HSV: $54,516- $129,576

For peer review only

Barnabas, 2002[25]	-	Diagnostic cost: $16-$100	Drug cost per couple per pregnancy: $37<br>Acute neonatal herpes treatment $1,500-50,000	C/S cost (personnel, supplies, surgery and ward care): $11,084	Labour cost: $200-1628<br>Counselling cost: $12-$19	Neonatal care after C/S: $884<br>Long term care for neonatal herpes: $140,766 - $273,712
Binkin, 1989[39]	Pregnant women with HSV	Viral culture: $30	NR	Hospitalisation for complication: $300-698<br>Hospital care associated with neonatal herpes: $25,000<br>Delivery: $2,300-3,600	NR	Long term care for neonatal herpes: $125,000-$250,000
Little, 2005[42]	Women with a history of diagnosed genital HSV	NR	Acyclovir (prophylaxis) from 36 weeks of gestation: $46	Delivery: $4,939-9,490<br>Hospitalisation: $32,483	NR	Lifetime cost of care of neonatal HSV: $349,7533-$1,049,260
Randolph, 1996[43]	Antenatal women with recurrent genital HSV	Laboratory: $35	Acyclovir 400mg (200caps): $228	Delivery: $3,500	Labour: $74	Lifetime cost of care of neonatal HSV: $85,000- 255,000
Rouse, 2000[44]	Antenatal women	Laboratory: $4 – 13	NR	Hospitalisation for neonatal care: $11,126	Labour: $3.50-10.50	Lifetime cost of care of neonatal HSV: $48,519- 163,879
Scott, 1998[31]	-	HSV culture: $80	Acyclovir 400mg tds for 4 weeks: $180	Hospitalisation for neonatal care: $480-1470<br>Delivery: $5,321 – 9,039	NR	NR

For peer review only

Thung, 2005[45]	Married women	HSV screening: $37.5-$75	Acyclovir 400mg tds for 4 weeks: $71	Delivery: $4,281 - 9,283	Counselling cost: $13	Lifetime cost of care of neonatal HSV: $13,202 – 325,602
Tuite, 2010[32]	Pregnant women	ELISA test: $7-$14	NR	Delivery: $5680- 8780	NR	Lifetime cost and consequence of neonatal HSV: $164,870
**Neonatal herpes management**						
Ambroggio, 2009[37]	Neonates with HSV and received intravenous acyclovir and discharge from Paediatric Health Information System	NR	Median pharmaceutical (not specified): $4,231<br>Median Imaging: $2,010	Median hospital charge: $37,431	NR	NR
Donda, 2019[41]	Patients aged 28 days and above from the French national hospital discharge database	NR	NR	Hospitalisation: $27,843	NR	NR
Flagg, 2011[47]	Neonates with ICD-9 codes for neonatal HSV	NR	NR	Hospitalisation: $92,664	NR	NR

For peer review only

	in the National Inpatient Sample from 2003-2014					
Mahant, 2019[46]	Records of neonates from the Medicaid claims database from 2009 - 2015	NR	NR	Hospitalisation: $32,683<br>Hospital readmission: $31,531<br>ED visit: $527	NR	NR

*All costs are mean costs except where explicitly labelled as median costs.

C/S – Caesarean section; ED - Emergency department; NR – Not reported

For peer review only

Table 3: Detailed description of studies reporting resource utilzation

Author, year	Healthcare seeking and diagnosis	Treatment phase
**Genital ulcer disease among adults/adolescents**		
Aslam, 2012[14]	• 74.1-93.2% sought care once within 12 months<br>• 6.8-25.9% sought care twice to 8x a year	
Desharnais, 1996[40]		• Oral treatment only: 16.1%<br>• IV treatment: 16.2%<br>• Hospital stay: 5.4 days
Fisman, 2005[15]		• 33.3% used antiviral drugs for HSV<br>• 15.8% had pregnancy complicated by HSV
Gilbert, 2010[16]	• 1.32% of young adults ever tested for genital herpes	
Patrick, 2004[17]	• 49% had viral culture performed<br>• 9% had antibody test<br>• 34% had physical examination	• 65% received oral antiviral therapy<br>• 18% received topical antiviral therapy<br>• 17% obtained alternative therapy
Tao, 2000[18]	• Estimated annual genital herpes visit 499,655 yearly<br>• 2% were inpatient visit<br>• 9% outpatient & ED visit<br>• 20% public STD clinic<br>• 69% private office based visit	
Xia, 2018[36]	From 2006-2013<br>• 245,484 ED visits with primary diagnosis of genital herpes or 37.3% of total ED visits for HSV<br>• Total charges: $278,335,295<br><br>ED visits trend from 2006 – 2013<br>• 24,747 (33.8%); 26,440 (34.1%); 27,484 (36.1%), 28,440 (36.5%); 33,258 (37.8%); 33,095 (38.3%); 35,501 (40.0%); 36,518 (40.3%)	
**Neonatal herpes prevention among pregnant mothers**		
Baker, 2004[38]	Estimates used in model<br>• 75% of partners will be willing to undergo HSV screening	Estimates used in model<br>• 1.32% women HSV-2 negative acquiring HSV during last 8 weeks of pregnancy<br>• 57% women or partner offered and accept antiviral therapy with testing

For peer review only

		• 82% women taking antivirals from week 36 compliant
Binkin, 1989[39]	Estimates used in model<br>• Average number of cultures per patient: 8	
Brocklehurst, 1995[19]	• 60% of obstetricians advocated some form of antenatal screening<br>Among those performing screening<br>• 64% perform regular viral cultures<br>• 54% recommend screening ≤34 weeks of gestation	• 92% of providers: visible active lesions at labor are cause for caesarean delivery
Brown, 2003[23]		• All women with HSV genital lesions noted at delivery had caesarean delivery (n=60) unless lesions not noted until too late to proceed with caesarean or lesions noted after delivery (n=14)
Heggarty, 2020[24]	For suspected primary genital HSV:<br>▪ 43.3% would conduct PCR of lesions plus HSV serology<br>▪ 39.9% would conduct PCR of lesions alone<br>▪ 0.4% would conduct HSV serology only	• If primary HSV GUD during pregnancy, 68.4% "always" and 11.6% "often" prescribe antiviral prophylaxis in 3rd trimester<br>• If recurrent HSV GUD during pregnancy, 55.1% "always" and 12.9% "often" prescribe antiviral prophylaxis in 3rd trimester<br>• 83% recommend caesarean delivery if genital HSV lesions suspected during labour
Kenny, 2013[22]	• 30% physicians will perform type-specific serology "most of the time" for patients with no history of herpes but partner with known HSV	• Antiviral suppressive therapy prescribed in third trimester by 90% of doctors (97% of obstetricians and 84% family physicians)<br>▪ 62% prescribed for any past history of GUD including pre-pregnancy<br>▪ 28% only after outbreak during pregnancy<br>▪ More commonly prescribed acyclovir (63%) than valacyclovir (38%)<br>• 65% offer elective caesarean if primary HSV in third trimester<br>• 95% of obstetricians and 84% of family physicians recommend caesarean delivery if HSV lesions during labour
Little, 2005[42]		Estimates used in model<br>• 24% of women will undergo caesarean delivery if no lesion was present

Lynn, 2017[20]	• 89% of patients had type-specific serology sent	• 63% received antiviral prophylaxis<br>▪ 98.5% received valacyclovir<br>▪ 1.5% received acyclovir<br>▪ Mean for initiating: week 36<br>• 29% of patients underwent caesarean delivery, none for HSV
Rouse, 2000[44]	Estimates used in model<br><br>• 75% of partners will be willing to undergo HSV screening	
Stankiewicz Karita, 2017[21]		• Antiviral suppressive therapy:<br>▪ 55% HSV-2 antibody-positive only<br>▪ 65% history of symptomatic GUD<br>• Similar caesarean section rates for women with/without history of HSV/genital herpes:<br>▪ 25% without history of HSV-2/GH<br>▪ 30% on suppressive treatment<br>• 28% without suppressive treatment
**Neonatal herpes management**		
Ahmad, 2015[11]	• CSF PCR performed in 92.3%<br>• Blood PCR performed in 48.7%	• 9.4 – 9.8% require ICU stay<br>• Hospital stay: 83.1-84.6hr<br>• 71.8% received acyclovir
Ambroggio, 2009[37]		• Median length of stay: 13 days (IQR 4-21)
Bernard, 2013[12]		• Mean hospital admission: 28 -34 days
Donda, 2019[41]		• Median length of stay: 20
Flagg, 2011[47]		• Mean length of stay: 22 days<br>• Median length of stay: 2- days
Mahant, 2019[46]		• Median hospital stay: 18 days<br>• Post discharge,<br>○ 45.7% required ED visit<br>○ 16.2% required rehospitalisation
Owusu-Edusei, 2015[13]		• Mean hospital stay: 10.8 (11.5)<br>• Mean hospital stay among those with admission >7 days: 18.5 (12.5)

For peer review only

Figure legend

Figure 1. Flow diagram of study selection process

For peer review only

References

1. Gupta R, Warren T, Wald A. Genital herpes. *The Lancet* 2007;370(9605):2127-37. doi: 10.1016/S0140-6736(07)61908-4
2. James C, Harfouche M, Welton N, et al. Herpes simplex virus: global infection prevalence and incidence estimates, 2016. *Bull World Health Organ* 2020;98:315-29. doi: 10.2471/BLT.19.237149
3. Looker KJ, Johnston C, Welton NJ, et al. The global and regional burden of genital ulcer disease due to herpes simplex virus: a natural history modelling study. *BMJ global health* 2020;5(3):e001875.
4. Pinninti SG, Kimberlin DW. Maternal and neonatal herpes simplex virus infections. *American journal of perinatology* 2013;30(02):113-20.
5. Looker KJ, Welton NJ, Sabin KM, et al. Global and regional estimates of the contribution of herpes simplex virus type 2 infection to HIV incidence: a population attributable fraction analysis using published epidemiological data. *Lancet Infect Dis* 2020;20(2):240-49. doi: 10.1016/s1473-3099(19)30470-0 [published Online First: 2019/11/23]
6. Gottlieb SL, Low N, Newman LM, et al. Toward global prevention of sexually transmitted infections (STIs): the need for STI vaccines. *Vaccine* 2014;32(14):1527-35.
7. Freeman EE, White RG, Bakker R, et al. Population-level effect of potential HSV2 prophylactic vaccines on HIV incidence in sub-Saharan Africa. *Vaccine* 2009;27(6):940-46. doi: https://doi.org/10.1016/j.vaccine.2008.11.074
8. Belshe RB, Heineman TC, Bernstein DI, et al. Correlate of immune protection against HSV-1 genital disease in vaccinated women. *J Infect Dis* 2014;209(6):828-36. doi: 10.1093/infdis/jit651 [published Online First: 2013/11/29]
9. Turner HC, Lauer JA, Tran BX, et al. Adjusting for Inflation and Currency Changes Within Health Economic Studies. *Value in Health* 2019
10. The World Bank. GDP deflator: linked series (annual %) 2019 2019 [Available from: https://data.worldbank.org/indicator/NY.GDP.DEFL.KD.ZG.AD accessed 25 November 2019.
11. Ahmad FA, Storch GA, Miller AS. Impact of an institutional guideline on the care of neonates at risk for herpes simplex virus in the emergency department. *Pediatric emergency care* 2017;33(6):396-401.
12. Bernard S, Mailles A, Stahl J. Epidemiology of infectious encephalitis, differences between a prospective study and hospital discharge data. *Epidemiology & Infection* 2013;141(11):2256-68.
13. Owusu-Edusei Jr K, Flagg EW, Gift TL. Hospitalization cost per case of neonatal herpes simplex virus infection from claims data. *Journal of pediatric nursing* 2015;30(2):346-52.
14. Aslam M, Kropp RY, Jayaraman G, et al. Genital herpes in Canada: Deciphering the hidden epidemic. *Canadian Journal of Infectious Diseases and Medical Microbiology* 2012;23(1):e6-e9.
15. Fisman D. Health related quality of life in genital herpes: a pilot comparison of measures. *Sexually transmitted infections* 2005;81(3):267-70.
16. Gilbert LK, Levandowski BA, Roberts CM. Characteristics associated with genital herpes testing among young adults: assessing factors from two national data sets. *Journal of American College Health* 2010;59(3):143-50.
17. Patrick D, Rosenthal S, Stanberry L, et al. Patient satisfaction with care for genital herpes: Insights from a global survey. *Sexually transmitted infections* 2004;80(3):192-97.
18. Tao G, Kassler WJ, Rein DB. Medical care expenditures for genital herpes in the United States. *Sexually transmitted diseases* 2000;27(1):32-38.

For peer review only - http://bmjopen.bmj.com/site/about/guidelines.xhtml

19. Brocklehurst P, Carney O, Ross E, et al. The management of recurrent genital herpes infection in pregnancy: A postal survey of obstetric practice. *British Journal of Obstetrics and Gynaecology* 1995;102(10):791-97.
20. Lynn N, Murphy S, Cunningham O, et al. HSV management in pregnancy at a joint antenatal-genitourinary clinic in a large maternity hospital in Dublin, Ireland-a model of care. *Sexually Transmitted Infections* 2017;93 (Supplement 1):A91.
21. Stankiewicz Karita HC, Moss NJ, Laschansky E, et al. Invasive obstetric procedures and cesarean sections in women with known herpes simplex virus status during pregnancy. *Open Forum Infectious Diseases* 2017;4(4) doi: 10.1093/ofid/ofx248
22. Kenny K, Leung W, Stephanson K, et al. Clinical practice in prevention of neonatal HSV infection: A survey of obstetrical care providers in Alberta. *Journal of Obstetrics and Gynaecology Canada* 2013;35(2):131-37. doi: https://doi.org/10.1016/S1701-2163(15)31017-3
23. Brown ZA, Wald A, Morrow RA, et al. Effect of serologic status and cesarean delivery on transmission rates of herpes simplex virus from mother to infant. *Jama* 2003;289(2):203-09.
24. Heggarty E, Sibiude J, Mandelbrot L, et al. Genital herpes and pregnancy: Evaluating practices and knowledge of French health care providers. *European journal of obstetrics, gynecology, and reproductive biology* 2020;249:84-91. doi: 10.1016/j.ejogrb.2020.02.039 [published Online First: 2020/03/04]
25. Barnabas R, Carabin H, Garnett G. The potential role of suppressive therapy for sex partners in the prevention of neonatal herpes: a health economic analysis. *Sexually transmitted infections* 2002;78(6):425-29.
26. Owusu-Edusei Jr K, Chesson HW, Gift TL, et al. The estimated direct medical cost of selected sexually transmitted infections in the United States, 2008. *Sexually transmitted diseases* 2013b;40(3):197-201.
27. Szucs TD, Berger K, Fisman DN, et al. The estimated economic burden of genital herpes in the United States. An analysis using two costing approaches. *BMC infectious diseases* 2001;1(1):5.
28. Vickerman P, Devine A, Foss AM, et al. The cost-effectiveness of herpes simplex virus-2 suppressive therapy with daily aciclovir for delaying HIV disease progression among HIV-1-infected women in South Africa. *Sexually Transmitted Diseases* 2011;38(5):401-09. doi: 10.1097/OLQ.0b013e31820b8bc8
29. Vickerman P, Ndowa F, Mayaud P. Cost-effectiveness of incorporating episodic treatment for Herpes simplex virus type-2 (HSV-2) into the syndromic algorithm for genital ulcer disease. *Sexually Transmitted Infections* 2008;84:243-48.
30. Owusu-Edusei Jr K, Nguyen HT, Gift TL. Utilization and cost of diagnostic methods for sexually transmitted infection screening among insured American youth, 2008. *Sexually transmitted diseases* 2013a;40(5):354-61.
31. Scott LL, Alexander J. Cost-effectiveness of acyclovir suppression to prevent recurrent genital herpes in term pregnancy. *American journal of perinatology* 1998;15(01):57-62.
32. Tuite AR, McCabe CJ, Ku J, et al. Projected cost-savings with herpes simplex virus screening in pregnancy: towards a new screening paradigm. *Sexually transmitted infections* 2011;87(2):141-48.
33. Korenromp EL, Wi T, Resch S, et al. Costing of national STI program implementation for the global STI control strategy for the health sector, 2016-2021. *PLoS One* 2017;12(1):e0170773.
34. Fisman DN, Lipsitch M, HOOK III EW, et al. Projection of the future dimensions and costs of the genital herpes simplex type 2 epidemic in the United States. *Sexually transmitted diseases* 2002;29(10):608-22.
35. Almonte-Vega L, Colón-Vargas M, Luna-Jarrín L, et al. Cost analysis of treatment strategies for the control of HSV–2 infection in the U.S.: A mathematical modeling-based case study. *Mathematical Biosciences* 2020;324:108347. doi: https://doi.org/10.1016/j.mbs.2020.108347

36. Xia F, Fuhlbrigge M, Dommasch E, et al. Cost of Routine Herpes Simplex Virus Infection Visits to US Emergency Departments 2006–2013. *Western Journal of Emergency Medicine* 2018;19(4):689.
37. Ambroggio L, Lorch SA, Mohamad Z, et al. Congenital anomalies and resource utilization in neonates infected with herpes simplex virus. *Sexually transmitted diseases* 2009;36(11):680.
38. Baker D, Brown Z, Hollier LM, et al. Cost-effectiveness of herpes simplex virus type 2 serologic testing and antiviral therapy in pregnancy. *American journal of obstetrics and gynecology* 2004;191(6):2074-84.
39. Binkin NJ, Koplan JP. The high cost and low efficacy of weekly viral cultures for pregnant women with recurrent genital herpes: a reappraisal. *Medical Decision Making* 1989;9(4):225-30.
40. DesHarnais S, Simpson KN, Paul JE. Variations in practice patterns: antiviral drug use in hospitalized patients with herpes infections. *American Journal of Medical Quality* 1996;11(1):33-42.
41. Donda K, Sharma M, Amponsah JK, et al. Trends in the incidence, mortality, and cost of neonatal herpes simplex virus hospitalizations in the United States from 2003 to 2014. *Journal of Perinatology* 2019;39(5):697-707. doi: 10.1038/s41372-019-0352-7
42. Little SE, Caughey AB. Acyclovir prophylaxis for pregnant women with a known history of herpes simplex virus: a cost-effectiveness analysis. *American journal of obstetrics and gynecology* 2005;193(3):1274-79.
43. Randolph AG, Hartshorn RM, Washington AE. Acyclovir prophylaxis in late pregnancy to prevent neonatal herpes: a cost-effectiveness analysis. *Obstetrics & Gynecology* 1996;88(4):603-10.
44. Rouse DJ, Stringer JS. An appraisal of screening for maternal type-specific herpes simplex virus antibodies to prevent neonatal herpes. *American journal of obstetrics and gynecology* 2000;183(2):400-06.
45. Thung SF, Grobman WA. The cost-effectiveness of routine antenatal screening for maternal herpes simplex virus–1 and–2 antibodies. *American journal of obstetrics and gynecology* 2005;192(2):483-88.
46. Mahant S, Hall M, Schondelmeyer AC, et al. Neonatal herpes simplex virus infection among Medicaid-enrolled children: 2009–2015. *Pediatrics* 2019;143(4):e20183233. doi: 10.1542/peds.2018-3233
47. Flagg EW, Weinstock H. Incidence of neonatal herpes simplex virus infections in the United States, 2006. *Pediatrics* 2011;127(1):e1-e8.
48. Fisman D, Hook E, Goldie S. Estimating the costs and benefits of screening monogamous, heterosexual couples for unrecognised infection with herpes simplex virus type 2. *Sexually transmitted infections* 2003;79(1):45-52.
49. Waitzman NJ, Romano PS, Scheffler RM. Estimates of the economic costs of birth defects. *Inquiry* 1994:188-205.
50. Looker KJ, Elmes JAR, Gottlieb SL, et al. Effect of HSV-2 infection on subsequent HIV acquisition: an updated systematic review and meta-analysis. *Lancet Infect Dis* 2017;17(12):1303-16. doi: 10.1016/S1473-3099(17)30405-X [published Online First: 08/23]
51. Gottlieb SL, Deal CD, Giersing B, et al. The global roadmap for advancing development of vaccines against sexually transmitted infections: update and next steps. *Vaccine* 2016;34(26):2939-47.

For peer review only

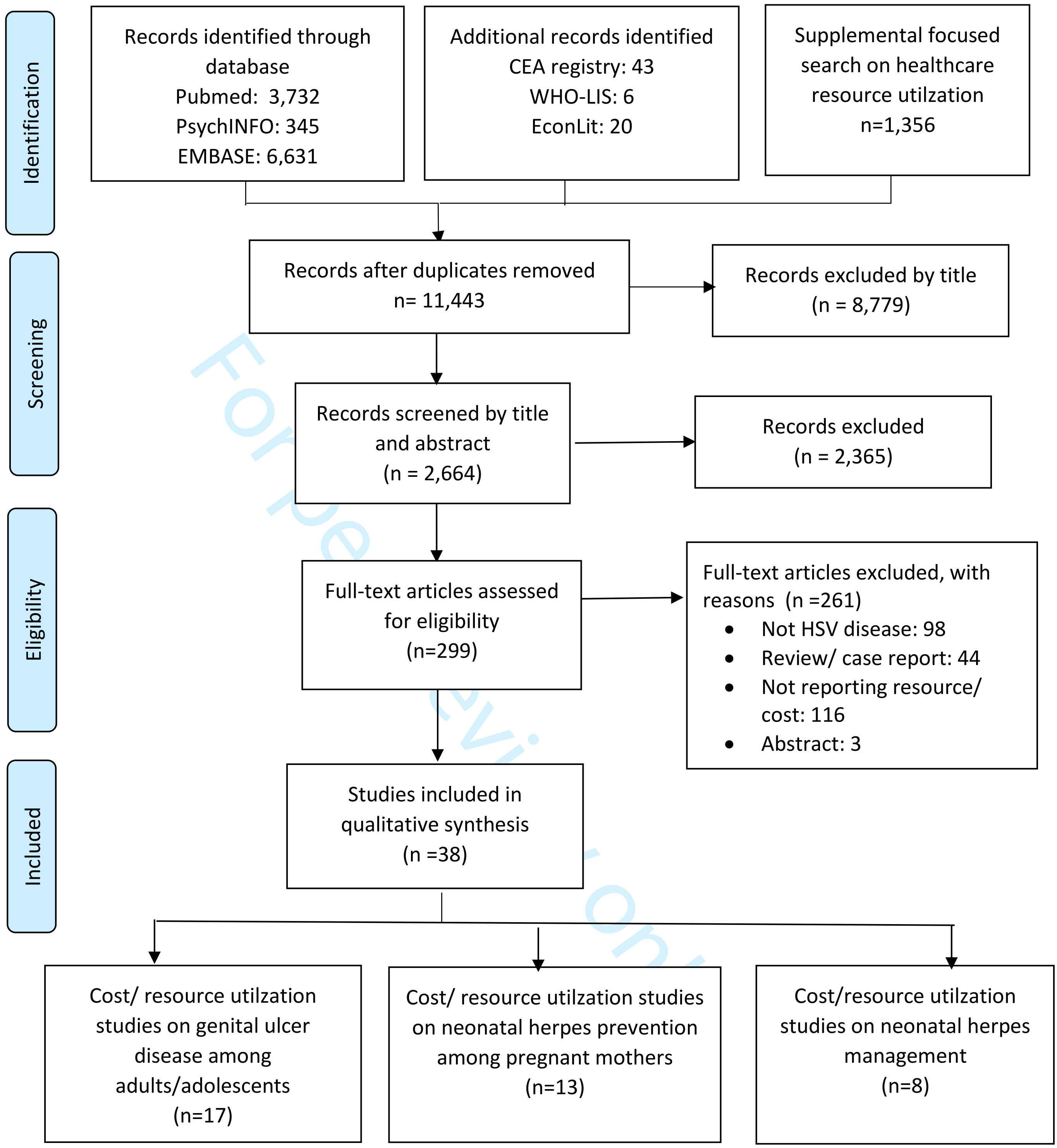
Identification
Screening
Eligibility
Included
Records identified through database
Pubmed: 3,732
PsychINFO: 345
EMBASE: 6,631
Additional records identified
CEA registry: 43
WHO-LIS: 6
EconLit: 20
Supplemental focused search on healthcare resource utilzation
n=1,356
Records after duplicates removed
n= 11,443
Records excluded by title
(n = 8,779)
Records screened by title and abstract
(n = 2,664)
Records excluded
(n = 2,365)
Full-text articles assessed for eligibility
(n=299)
Full-text articles excluded, with reasons (n =261)
Not HSV disease: 98
Review/ case report: 44
Not reporting resource/ cost: 116
Abstract: 3
Studies included in qualitative synthesis
(n =38)
Cost/ resource utilzation studies on genital ulcer disease among adults/adolescents
(n=17)
Cost/ resource utilzation studies on neonatal herpes prevention among pregnant mothers
(n=13)
Cost/resource utilzation studies on neonatal herpes management
(n=8)

APPENDIX

TABLE OF CONTENTS

Search methodology 2

Text 1: Keyword terms used in the search.. 3

Text 2: Keywords used in focused search using exploding terms.. 4

Table 1: Detailed cost incurred in genito-ulcer diseases due to HSV 5

Table 2: Detailed cost associated with genitoulcer disease prevention in people living with HIV8

Table 3: Detailed cost associated with neonatal herpes prevention/management.................... 9

Figure 1: Methodological quality of included economic studies using CHEC Checklist............. 13

Figure 2: Methodological quality of included costing studies using Larg and Moss Checklist ... 14

For peer review only

Search methodology

1. Search strategy

- The current search strategy was developed based upon keywords which have been used in previous existing HSV reviews commissioned by WHO. All search keywords used were subsequently cross-checked with the following articles to ensure comprehensiveness

    - Looker, 2017. Effect of HSV-2 infection on subsequent HIV acquisition: an updated systematic review and meta-analysis
    - Khard, 2019. The Epidemiology of Herpes Simplex Virus Type 1 in Asia: Systematic Review, Meta-analyses, and Meta-regressions
    - Looker,2012. Global estimates of prevalent and incident herpes simplex virus type 2 infections in 2012. PLoS One 2015;10(1) : e114989-e89. Doi: 10.1371/journal.pone. 0114989
- The following databases were identified for the search including: PubMed, PsychINFO, EMBASE, Centre for Review and Dissemination, EconLit, CEA registry and WHO Library Database (WHOLIS)

2. **Keywords search** was revised to compare between a) search including exploding terms and b) search including title and abstract. A total of 10,113 articles was found for search when terms were exploded versus 5,966 when these terms were not exploded. As such, the methods will only use search including exploding terms to minimize the risk of missing relevant study despite its low specificity. The initial search was performed in April 2019, with an updated search in October 2019.

3. Neonate search

- We also conducted search over again using all relevant HSV terms with neonate as keyword. All articles identified in the search overlapped with existing broader search, thereby there is no need to add neonate as key words

For peer review only

Text 1: Keyword terms used in the search

No.	Keyword
#1	Genital ulcer disease.mp.
#2	Herpes labialis.mp.
#3	Herpes genitalis.mp.
#4	Genital herpes.mp.
#5	Herpesvirus.mp.
#6	Herpes virus.mp.
#7	HSV.mp.
#8	Herpes simplex.mp.
#9	1 or 2 or 3 or 4 or 5 or 6 or 7 or 8
#10	Healthcare util*ation.mp.
#11	Util*ation.mp.
#12	Physician visit.mp.
#13	General practitioner visit.mp.
#14	Hospital visit.mp.
#15	Clinic visit.mp.
#16	Hospital stay.mp.
#17	Hospitali*ation.mp.
#18	Hospital readmission.mp.
#19	Cost.mp.
#20	Cost-effectiveness.mp.
#21	Cost-utility.mp.
#22	Cost-benefit.mp.
#23	Cost-minimi*ation.mp.
#24	Counselling.mp.
#25	Seek care.mp.
#26	Behavio*r.mp.
#27	10 or 11 or 12 or 13 or 14 or 15 or 16 or 17 or 18 or 19 or 20 or 21 or 22 or 23 or 24 or 25 or 26
#28	9 and 27

For peer review only

Text 2: Keywords used in focused search using exploding terms.

No.	Keyword
#1	Genital ulcer disease.mp.
#2	Herpes labialis.mp.
#3	Herpes genitalis.mp.
#4	Genital herpes.mp.
#5	Herpesvirus.mp.
#6	Herpes virus.mp.
#7	HSV.mp.
#8	Herpes simplex.mp.
#9	1 or 2 or 3 or 4 or 5 or 6 or 7 or 8
#10	pregnancy.mp.
#11	pregnant.mp.
#12	c*esarean.mp.
#13	delivery.mp.
#14	10 or 11
#15	12 or 13
#16	9 AND 14 AND 15

For peer review only

Table 1: Detailed cost incurred in genito-ulcer diseases due to HSV

Author,year	Outcomes	Unit cost ($) in original year	Unit cost in 2018 ($)
**Medication costs**			
Vickerman, 2008	One dose of IV benzathine penicillin 2.4MU	0.15 - 0.48	0.19-0.59
Vickerman, 2008	One tab of 500mg ciprofloxacin	0.10 - 0.21	0.12 - 0.26
Vickerman, 2008	One cap of 200mg acyclovir	0.53- 5.24	0.66 – 6.48
Fisman, 2003	Acyclovir therapy for relapse patients	16.00	22.72
Fisman, 2003	Acyclovir cost for suppressive monthly therapy	40.00	56.80
Almonte-Vega, 2020	Acyclovir therapy	86.33	86.33
Fisman, 2003	Condom cost	0.10	0.14
Szucs, 2001	Pharmacological treatment 1st episode (NS)	64.00	94.86
Szucs, 2001	Pharmacological treatment recurrent episode (NS)	131.00	194.18
Vickerman, 2008	Needle and syringe cost	0.15	0.19
Tao, 2000	Pharmacy claim	52.00	73.84
**Laboratory test**			
Szucs, 2001	Antibiotic testing based on expert opinion	76.50	113.39
Szucs, 2001	Antibiotic testing in first episode based on claims	12.80	18.97
Szucs, 2001	Antibiotic testing in subsequent episode based on claims	6.50	9.63
Szucs, 2001	Complete blood count based on expert opinion	21.29	31.56
Szucs, 2001	Complete blood count in first episode based on claims	4.60	6.82
Szucs, 2001	Complete blood count in subsequent episode based on claims	1.50	2.22
Szucs, 2001	Microbiological test for first GUD episode	17.60	26.09
Szucs, 2001	Microbiological test for subsequent GUD episode	6.70	9.93
Szucs, 2001	Microbiological test based on expert opinion	38.39	56.90
Almonte-Vega, 2020	Microbiological lab test	80.17	80.17

For peer review only

Fisman, 2003	Western blot	60.00	85.20
Szucs, 2001	Urine analysis based on expert opinion	12.59	18.66
Szucs, 2001	Urine analysis in first episode based on claims	4.60	6.82
Szucs, 2001	Urine analysis in subsequent episode based on claims	3.20	4.74
**Hospitalisation cost**			
Fisman, 2003	Excess obstetrical cost associated with history of symptomatic HSV2 infection	300.00	425.98
Fisman, 2003	Excess obstetrical cost due to symptomatic HSV2 infection	310.00	440.18
Tao, 2000	Inpatient cost	2,530.00	3592.46
Szucs, 2001	Hospital day	669.00	991.63
**Clinic visit**			
Fisman, 2003	Clinic visit related to GUD (for physician time, test, lost wages due to 2hr patient time)	120.00	170.39
Szucs, 2001	Clinical examination based on expert opinion	40.33	59.78
Szucs, 2001	Clinical examination first episode based on claims	39.80	58.99
Szucs, 2001	Clinical examination on subsequent episode based on claims	36.20	53.66
Szucs, 2001	Physician consultation based on expert opinion	73.00	108.21
Szucs, 2001	Physician consultation in first episode based on claims	62.60	92.79
Szucs, 2001	Physician consultation in subsequent episode based on claims	59.60	88.34
Tao, 2000	Outpatient and ED	59.00	83.78
Fisman, 2003	Outpatient visit	120.00	170.39
Tao, 2000	Office based physician and public clinic	67.00	95.14
Almonte-Vega, 2020	Consultation, clinical examination and diagnostic	161.85	161.85
Vickerman, 2008	Counselling cost	0.28	0.35
**Other costs**			
Szucs, 2001	Others miscellaneous cost related to first GUD episode(not reported)	33.00	48.91

For peer review only

Szucs, 2001	Others miscellaneous cost related to recurrent GUD episode(not reported)	12.30	18.23
Szucs, 2001	Production losses	60.00	88.94
Szucs, 2001	Total cost of active GUD	355.00	526.20
Szucs, 2001	Total cost of incident GUD	235.00	348.33
Szucs, 2001	Total cost of prevalent GUD	166.00	246.06
Szucs, 2001	Total cost of recurrent GUD	499.00	739.65
Fisman, 2003	Treatment cost for men assuming 2 clinic visit, 7 day course of acyclovir (400mg tds) and 2 days off work	450.00	638.97
Fisman, 2003	Treatment cost for women assuming 2 clinic visit, 7 day course of acyclovir (400mg tds) and 2 days off work	800.00	1135.95

For peer review only

Table 2: Detailed cost associated with genitoulcer disease prevention in people living with HIV

Author, year	Outcomes	Unit cost ($) in original year	Unit cost in 2018 ($)
Vickerman, 2011	Acyclovir 400mg	0.07	0.07
Vickerman, 2011	Staff cost- for default tracer over 3 months	24.00	22.32
Vickerman, 2011	Staff cost for training for STI diagnosis and default tracer	0.46	0.43
Vickerman, 2011	Labour cost for senior nurse	2.52	2.34
Vickerman, 2011	Counselling cost (10 mins)	0.88	0.82
Vickerman, 2011	CD-4 count test	7.90	7.35

NB- Cost reported after adjustment in 2017 were lower than those in the original study due to exchange rates at the time of study.

For peer review only

Table 3: Detailed cost associated with neonatal herpes prevention/management

Author,year	Outcomes	Unit cost (USD$)	Unit cost in 2017 ($)
**Medication costs**			
Randolph, 1996	One cap of acyclovir 400mg	1.14	1.72
Baker, 2004	Pharmaceutical cost for pregnant women	6.18	8.10
Baker, 2004	Pharmaceutical cost for partner	3.93	5.15
Baker, 2004	Valacyclovir 500mg	3.95	5.18
Baker, 2004	Valacyclovir 1000mg	6.49	8.51
Baker, 2004	Acyclovir 400mg	1.96	2.57
Barnabas, 2002	Acyclovir treatment for a couple for one pregnancy	37.00	51.37
Scott, 1998	Acyclovir 400mg	1.71	2.58
**Laboratory test**			
Randolph, 1996	Screening using herpes culture	35.00	52.83
Thung, 2005	HSV1 or 2 screening cost	37.50	49.15
Thung, 2005	HSV 1 and 2 screening	75.00	98.31
Rouse, 2000	HSV-2 antibody assay	4.00	5.68
Rouse, 2000	HSV-2 labour and reagent cost, QC etc	9.00	12.78
Tuite, 2010	ELISA screening for HSV	7.00	7.96
Scott, 1998	HSV culture	80.00	120.75
Baker, 2004	Labor and supplies for HSV-2 specific test	15.58	20.42
Baker, 2004	HSV test for partner	40.53	53.12
Barnabas, 2002	Diagnostic kit cost	70.00	97.18
Binkin, 1989	Viral culture	30.00	52.97
**Hospitalisation cost**			
Scott, 1998	Vaginal delivery with metritis, includes labour, delivery, postpartum and professional	8439.00	12,737.15

For peer review only

Scott, 1998	Vaginal delivery without metritis, includes labour, delivery, postpartum and professional	5,321.00	8,031.09
Ambroggio, 2009	Hospital charges	62,050.90	70,544.69
Tuite, 2010	Vaginal delivery	5,680.00	6,457.50
Little, 2005	Vaginal delivery	4,939.00	6,104.17
Randolph, 1996	Caesarean delivery over vaginal	3,500.00	5,282.62
Tuite, 2010	Caesarean section	8,780.00	9,981.84
Tao, 1999	Caesarean attributable to genital herpes	1,922.00	2729.13
Little, 2005	Caesarean delivery	9,490.00	11,728.80
Little, 2005	Caesarean delivery with lesion	7,608.00	9,402.82
Scott, 1998	Caesarean delivery with metritis, includes labour, delivery, postpartum and professional	9,039.00	13,642.74
Scott, 1998	Caesarean delivery without metritis, includes labour, delivery, postpartum and professional	10,553.00	15,927.85
Thung, 2005	Elective caesarean	7,425.00	9,732.37
Thung, 2005	Labour caesarean	9,283.00	12,167.75
Little, 2005	Hospital care due to neonatal herpes infection	32,483.00	40,146.12
Rouse, 2000	Hospital care due to neonatal herpes infection	11,126.00	15,798.28
Baker, 2004	Caesarean delivery	5,021.00	6,581.31
Binkin, 1989	Hospital stay due to complication	698.00	1,232.38
Binkin, 1989	Hospital care due to neonatal herpes infection	25,000.00	44,139.53
Barnabas, 2002	Caesarean delivery with lesion	11,084.00	15,388.48
**Clinic visit**			
Scott, 1998	Clinic visit	39.50	59.62
Thung, 2005	Counselling cost	13.00	17.04
Rouse, 2000	Counselling cost (10 mins)	3.50	4.97
Rouse, 2000	Counselling cost for couple (30 mins)	10.50	14.91
Randolph, 1996	Follow-up call and office visit following screening	74.00	111.69
Barnabas, 2002	Pharmacy dispensing and education cost	3.00	4.17
Barnabas, 2002	Obstetrician counselling and testing salary for screening	19.00	26.38

For peer review only

Barnabas, 2002	Obstetrician counselling and testing salary for treatment	12.00	16.66
**Long-term care cost**			
Scott, 1998	Infant treated for HSV (include drug and culture)	1,470.00	2,218.70
Scott, 1998	Neonatal care if using caesarean delivery	821.00	1,239.15
Scott, 1998	Neonatal care if using vaginal delivery	480.00	724.47
Randolph, 1996	Neonatal herpes acute hospital care	10,160.00	15,334.69
Thung, 2005	Acute and long term care for normal/mild deficit	13,202.00	17,304.61
Randolph, 1996	Long term medical cost for moderate disability (Y1-Y65)	85,000.00	128,292.20
Thung, 2005	Acute and long term care for moderate deficit	134,202.00	175,906.12
Little, 2005	Lifetime cost and care for moderately disabled child	349,753.00	432,263.77
Rouse, 2000	Lifetime cost and care for moderately disabled child 1999	48,519.00	68,894.21
Baker, 2004	Lifetime medical and institutionalised cost for neonatal herpes	92,350.00	121,048.35
Binkin, 1989	Lifetime cost and care for moderately disabled child	125,000.00	220,697.66
Fisman, 2003	Lifetime cost of neonatal HSV with moderate neurological sequel	97,000.00	13,7734.46
Randolph, 1996	Long term medical cost for severe disability (Y1-Y65)	255,000.00	384,876.59
Thung, 2005	Acute and long term care for severe deficit	325,602.00	426,784.88
Little, 2005	Lifetime cost and care for severely disabled child	1,049,260.00	1,296,792.56
Rouse, 2000	Lifetime cost and care for severely disabled child	163,879.00	232,698.82
Binkin, 1989	Lifetime cost and care for severely disabled child	250,000.00	441,395.33
Fisman, 2003	Lifetime cost of neonatal HSV with severe neurological sequel	291,000.00	413,203.38
Tuite, 2010	Lifetime cost of neonatal HSV	164,870.00	187,438.10
Fisman, 2003	Lifetime cost of neonatal HSV	110,000.0	156,193.72
Baker, 2004	Counselling cost nurse (15 mins)	5.98	7.84
Baker, 2004	Counselling cost physician (5 mins)	6.67	8.74
Baker, 2004	Labour cost and supplies	15.58	20.42
Baker, 2004	Total cost without screening program	1,181.35	1,548.46
Baker, 2004	Total cost with screening for women	1,211.95	1,588.57
Baker, 2004	Total cost with screening for women and partner	1,267.24	1,661.04

For peer review only

Barnabas, 2002	Maternal mortality cost	443,858.00	616,230.57
Thung, 2005	Mortality cost	13,202.00	17,304.61
Barnabas, 2002	Neonatal care after caesarean	885.00	1228.69
Barnabas, 2002	Medical services for care of neonatal herpes	273,712.00	380,008.25
Barnabas, 2002	Long term care for neonatal herpes	140,766.00	195,432.58
Barnabas, 2002	Caregiver cost for neonates due to neonatal herpes	149,943.00	208,173.47

For peer review only

Figure 1: Methodological quality of included economic studies using CHEC Checklist

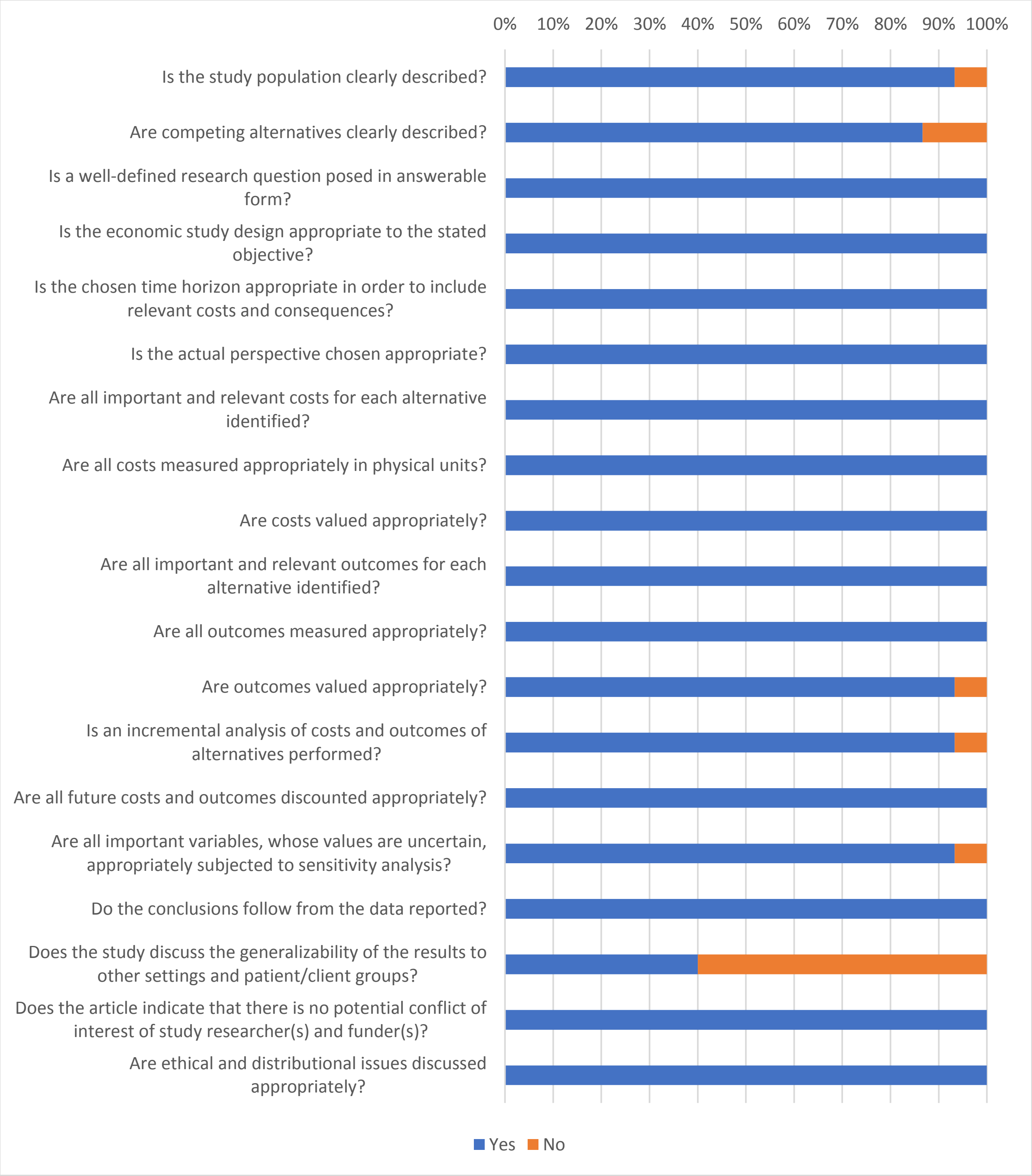

Figure 2: Methodological quality of included costing studies using Larg and Moss Checklist

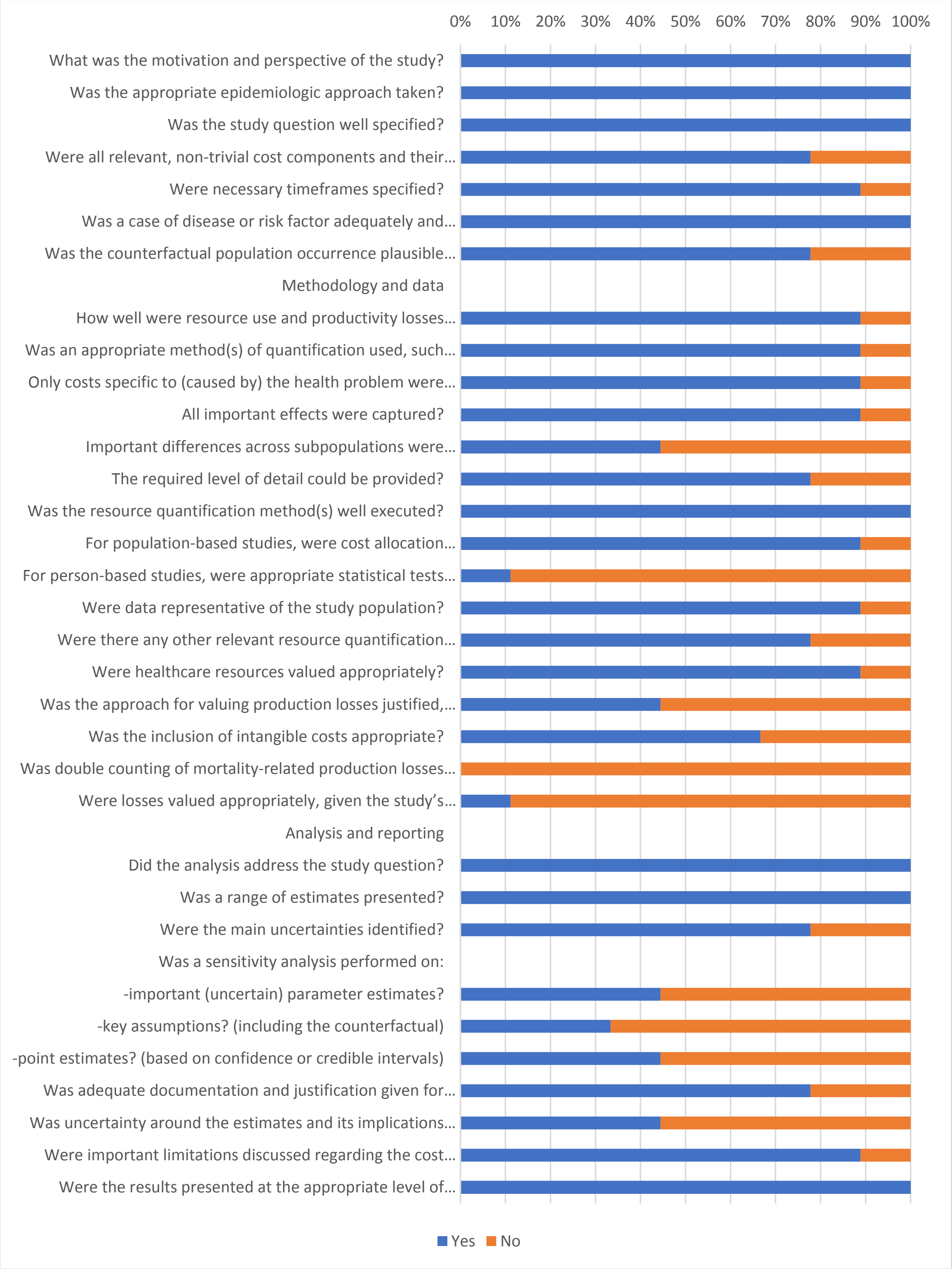

PRISMA 2009 Checklist

Section/topic	#	Checklist item	Reported on page #
**TITLE**			
Title	1	Identify the report as a systematic review, meta-analysis, or both.	1
**ABSTRACT**			
Structured summary	2	Provide a structured summary including, as applicable: background; objectives; data sources; study eligibility criteria, participants, and interventions; study appraisal and synthesis methods; results; limitations; conclusions and implications of key findings; systematic review registration number.	2
**INTRODUCTION**			
Rationale	3	Describe the rationale for the review in the context of what is already known.	4
Objectives	4	Provide an explicit statement of questions being addressed with reference to participants, interventions, comparisons, outcomes, and study design (PICOS).	4-5
**METHODS**			
Protocol and registration	5	Indicate if a review protocol exists, if and where it can be accessed (e.g., Web address), and, if available, provide registration information including registration number.	6
Eligibility criteria	6	Specify study characteristics (e.g., PICOS, length of follow-up) and report characteristics (e.g., years considered, language, publication status) used as criteria for eligibility, giving rationale.	6
Information sources	7	Describe all information sources (e.g., databases with dates of coverage, contact with study authors to identify additional studies) in the search and date last searched.	6
Search	8	Present full electronic search strategy for at least one database, including any limits used, such that it could be repeated.	6
Study selection	9	State the process for selecting studies (i.e., screening, eligibility, included in systematic review, and, if applicable, included in the meta-analysis).	6
Data collection process	10	Describe method of data extraction from reports (e.g., piloted forms, independently, in duplicate) and any processes for obtaining and confirming data from investigators.	7
Data items	11	List and define all variables for which data were sought (e.g., PICOS, funding sources) and any assumptions and simplifications made.	7
Risk of bias in individual studies	12	Describe methods used for assessing risk of bias of individual studies (including specification of whether this was done at the study or outcome level), and how this information is to be used in any data synthesis.	7
Summary measures	13	State the principal summary measures (e.g., risk ratio, difference in means).	7
Synthesis of results	14	Describe the methods of handling data and combining results of studies, if done, including measures of consistency (e.g., I^2) for each meta-analysis.	7

For peer review only

PRISMA 2009 Checklist

Section/topic	#	Checklist item	Reported on page #
Risk of bias across studies	15	Specify any assessment of risk of bias that may affect the cumulative evidence (e.g., publication bias, selective reporting within studies).	7
Additional analyses	16	Describe methods of additional analyses (e.g., sensitivity or subgroup analyses, meta-regression), if done, indicating which were pre-specified.	7
**RESULTS**			
Study selection	17	Give numbers of studies screened, assessed for eligibility, and included in the review, with reasons for exclusions at each stage, ideally with a flow diagram.	8
Study characteristics	18	For each study, present characteristics for which data were extracted (e.g., study size, PICOS, follow-up period) and provide the citations.	8
Risk of bias within studies	19	Present data on risk of bias of each study and, if available, any outcome level assessment (see item 12).	8-9
Results of individual studies	20	For all outcomes considered (benefits or harms), present, for each study: (a) simple summary data for each intervention group (b) effect estimates and confidence intervals, ideally with a forest plot.	8-9
Synthesis of results	21	Present results of each meta-analysis done, including confidence intervals and measures of consistency.	-
Risk of bias across studies	22	Present results of any assessment of risk of bias across studies (see Item 15).	8
Additional analysis	23	Give results of additional analyses, if done (e.g., sensitivity or subgroup analyses, meta-regression [see Item 16]).	8-13
**DISCUSSION**			
Summary of evidence	24	Summarize the main findings including the strength of evidence for each main outcome; consider their relevance to key groups (e.g., healthcare providers, users, and policy makers).	14-16
Limitations	25	Discuss limitations at study and outcome level (e.g., risk of bias), and at review-level (e.g., incomplete retrieval of identified research, reporting bias).	15-16
Conclusions	26	Provide a general interpretation of the results in the context of other evidence, and implications for future research.	17
**FUNDING**			
Funding	27	Describe sources of funding for the systematic review and other support (e.g., supply of data); role of funders for the systematic review.	1

From: Moher D, Liberati A, Tetzlaff J, Altman DG, The PRISMA Group (2009). Preferred Reporting Items for Systematic Reviews and Meta-Analyses: The PRISMA Statement. PLoS Med 6(7): e1000097. doi:10.1371/journal.pmed1000097

For more information, visit: **www.prisma-statement.org**.

For peer review only - http://bmjopen.bmj.com/site/about/guidelines.xhtml

BMJ Open

Healthcare resource utilization pattern and costs associated with herpes simplex virus diagnosis and management : a systematic review

Journal:	*BMJ Open*
Manuscript ID	bmjopen-2021-049618.R1
Article Type:	Original research
Date Submitted by the Author:	28-Sep-2021
Complete List of Authors:	Lee, Shaun Wen Huey ; Monash University - Malaysia Campus, School of Pharmacy<br>Gottlieb, Sami L.; World Health Organization, Department of Reproductive Health and Research<br>Chaiyakunapruk, Nathorn; University of Utah Department of Pharmaceutics
**Primary Subject Heading**:	Health economics
Secondary Subject Heading:	Global health, Infectious diseases, Public health, Sexual health
Keywords:	HEALTH ECONOMICS, Health policy < HEALTH SERVICES ADMINISTRATION & MANAGEMENT, Public health < INFECTIOUS DISEASES

SCHOLARONE™
Manuscripts

I, the Submitting Author has the right to grant and does grant on behalf of all authors of the Work (as defined in the below author licence), an exclusive licence and/or a non-exclusive licence for contributions from authors who are: i) UK Crown employees; ii) where BMJ has agreed a CC-BY licence shall apply, and/or iii) in accordance with the terms applicable for US Federal Government officers or employees acting as part of their official duties; on a worldwide, perpetual, irrevocable, royalty-free basis to BMJ Publishing Group Ltd ("BMJ") its licensees and where the relevant Journal is co-owned by BMJ to the co-owners of the Journal, to publish the Work in this journal and any other BMJ products and to exploit all rights, as set out in our licence.

The Submitting Author accepts and understands that any supply made under these terms is made by BMJ to the Submitting Author unless you are acting as an employee on behalf of your employer or a postgraduate student of an affiliated institution which is paying any applicable article publishing charge ("APC") for Open Access articles. Where the Submitting Author wishes to make the Work available on an Open Access basis (and intends to pay the relevant APC), the terms of reuse of such Open Access shall be governed by a Creative Commons licence – details of these licences and which Creative Commons licence will apply to this Work are set out in our licence referred to above.

Other than as permitted in any relevant BMJ Author's Self Archiving Policies, I confirm this Work has not been accepted for publication elsewhere, is not being considered for publication elsewhere and does not duplicate material already published. I confirm all authors consent to publication of this Work and authorise the granting of this licence.

For peer review only

Healthcare resource utilization pattern and costs associated with herpes simplex virus diagnosis and management : a systematic review

Shaun Wen Huey Lee[1,2,3] , Sami L. Gottlieb[4], Nathorn Chaiyakunapruk[1,5]

[1] School of Pharmacy, Monash University Malaysia, Jalan Lagoon Selatan, 47500 Selangor, Malaysia

[2] School of Pharmacy, Taylor's University, Jalan Taylors, 47500 Selangor, Malaysia

[3]Center of Global Health, University of Pennsylvania, Philadelphia, Pennsylvania, USA

[3] Department of Sexual and Reproductive Health and Research, World Health Organization, Geneva, Switzerland

[4]Department of Pharmacy, College of Pharmacy, University of Utah, Salt Lake City, Utah, USA

Corresponding author:

Nathorn Chaiyakunapruk
Department of Pharmacotherapy
University of Utah College of Pharmacy
30 South 2000 East, Room 4964
Salt Lake City, UT 84112
Office: 801.585.3092
nathorn.chaiyakunapruk@utah.edu

Financial Support

This work was funded by the WHO Department of Sexual and Reproductive Health and Research, via support from the UNDP-UNFPA-UNICEF-WHO-World Bank Special Programme of Research, Development and Research Training in Human Reproduction (HRP) and the U.S. National Institute of Allergy and Infectious Diseases, part of the National Institutes of Health (U01 AI108543).

For peer review only

ABSTRACT

Objectives: Little is known about the economic burden of HSV across countries. This article aims to summarize existing evidence on estimates of costs and healthcare resource utilization associated with genital and neonatal HSV infection.

Design: Systematic literature review

Data sources: Seven databases were searched from inception to August 31st 2020. A focused search was performed to supplement the results.

Eligibility criteria: Studies which reported either healthcare resource utilization or costs associated with HSV-related healthcare, including screening, diagnosis and treatment of genital HSV infection and neonatal herpes prevention and treatment

Data extraction and synthesis: Two independent reviewers extracted data and assessed the risk of bias using the Larg and Moss's checklist. All data were summarized narratively

Results: Out of 11,443 articles, 38 were included. Most studies (35/38, 94.6%) were conducted in high income countries, primarily the United States, and were more often related to the prevention or management of neonatal herpes (n=21) than HSV genital ulcer disease (n=17). Most analyses were conducted before 2010. There was substantial heterogeneity in the reporting of HSV-related healthcare resource utilization, with 74% to 93% individuals who sought care for HSV, 11.6% to 68.4% individuals who received care, while neonates with herpes required a median of 6 to 34 hospitalisation days. The costs reported were similarly heterogeneous, with wide variation in methodology, assumptions, and outcome measures between studies. Cost for screening ranged from $7 to $100, treatment ranged from $0.53 to $35 for an episodic therapy, $240 to $2580 yearly for suppressive therapy, while hospitalisation for neonatal care ranged from $5,321-$32,683.

Conclusions: A paucity of evidence exists on healthcare resource utilization and costs associated with HSV infection, especially among low- and middle-income countries. Future research is needed on costs and healthcare utilization patterns to improve overall understanding of the global economic burden of HSV.

(298/300 words)

Keywords: herpes simplex virus; healthcare resource utilization; neonatal herpes; pregnancy; genital ulcer

For peer review only

Strengths and limitations of this study

- This is the first systematic review to assess the healthcare resource utilisation and costs associated with herpes simplex virus (HSV) infections.
- Comprehensive literature searches were conducted, which were supplemented by a focused search.
- Heterogeneity of study designs and outcome measures limited the meta-analysis of study results.
- Relatively few studies described the healthcare resource utilization patterns and cost of HSV, especially from low-middle income countries.

For peer review only

INTRODUCTION

Herpes simplex virus (HSV)-1 and HSV-2 are DNA viruses that belong to Alphaherpesviridae, a subfamily of the Herpesviridae family.[1] Both viruses can cause genital infection, which can have a profound impact on sexual and reproductive health. HSV-2 is almost entirely transmitted during sexual activity and is the most common cause of genital herpes, affecting more than one in every 8 individuals, or 491.5 million people, aged 15-49 years in 2016.[2] HSV-1 is the main cause of oral herpes but can also be transmitted to the genital area through oral sex. HSV-1 affects an estimated 3.7 billion people under age 50 globally, of which over 120 million may have genital infection.[2] While the prevalence of HSV infection is high globally, it varies widely by region. The highest prevalence of both HSV-1 (88% in females and males) and HSV-2 (44% in females; 25% in males) is in the African region, which is primarily comprised of low- and middle-income countries (LMIC).[1 2]

Genital HSV infection is lifelong and characterised by periodic reactivation. Many infections are asymptomatic or unrecognized, but up to a third of people may develop painful, recurrent genital sores known collectively as genital ulcer disease (GUD).[3] Antiviral medications can be taken episodically to shorten GUD outbreaks or taken daily (suppressive therapy) to reduce the number of outbreaks, but they are not curative. Pregnant women with genital HSV infection can also transmit the virus to their infants in the peripartum period, resulting in neonatal herpes.[4] Although this occurs only rarely, neonatal herpes has a high fatality and disability rate among surviving infants. As such, particularly in high-income countries (HIC), prevention measures such as caesarean section are often undertaken if a mother has active HSV lesions at delivery. Genital HSV-2 infection has also been linked to an increased risk of acquisition and transmission of human immunodeficiency virus (HIV) infection.[5]

The World Health Organization (WHO) has highlighted the need for a vaccine against HSV-2, due to large numbers of infections globally and the resulting disease consequences including GUD, neonatal herpes, and increased risk of HIV acquisition.[6-8] Multiple vaccine candidates have been studied to date with modelling studies showing that prevention of HSV-2 infection with a vaccine could potentially also reduce the incidence of HIV infection.[9] Vaccines targeting HSV-2 might also have benefits against HSV-1.[10] Understanding the potential value of HSV vaccines requires not only

For peer review only

predicting the impact of the vaccines on HSV-related disease burden, but also on its economic burden. However, little is known about the economic burden of HSV globally. As a first step in estimating HSV-related economic burden, we conducted a broad systematic review with the aim of summarizing all available evidence on costs and resource utilization associated with diagnosing, treating, and managing genital and neonatal HSV infection.

For peer review only

METHODS

The current study followed the guidelines of the Cochrane Handbook for Systematic Reviews of Intervention.[11] The review was reported in accordance with the Preferred Reporting Items for Systematic Review and Meta-Analyses.[12]

Data Sources and Search Strategy

We electronically searched for relevant articles published from database inception to August 31st 2020 in 7 databases: PubMed, PsychINFO, EMBASE, Centre for Review and Dissemination, EconLit, CEA registry and WHO Library Database (WHOLIS). The search strategy was based on a broad combined search string “Herpes Simplex Virus” AND “cost” OR “resource utilization” OR “econ*”, with no language restriction. A complete search strategy is detailed in Appendix Text 1. In addition, bibliographies of relevant articles were examined to identify potential studies not indexed in the aforementioned databases. A focused supplemental search on Google Scholar was performed using the keywords listed in Appendix Text 2 based upon the inclusion above.

Study Selection

Studies were included if they were original articles that investigated resource utilization patterns and costs related to HSV infection including the cost of any diagnostic tools, consultation time, treatment and hospital cost related to detecting and managing all types of HSV-1 or HSV-2 related neonatal and genital infections and associated disease outcomes. We included articles which were published in English languages.

Data Extraction and Quality Assessment

The study followed a 2-stage process, where two independent reviewers screened the titles and abstracts for relevant studies, before the full texts were screened by another two independent reviewers for eligibility. Relevant information from the identified studies was extracted independently by two reviewers using a standardized data extraction sheet. At all stages, any disagreement was resolved by discussion between reviewers through consensus. Information

For peer review only

collected from the data extraction sheet included: 1) general study information including country of the study, 2) HSV subtype and disease, 3) study design, 4) healthcare resource utilization, 5) costs of relevant tests, clinical care, hospitalisation, and medications, and 6) summary estimates of HSV-related economic burden. Methodological quality of all included economic studies was assessed using the Consensus Health Economic Criteria (CHEC) list. This checklist has been recommended for critically appraising published economic evaluations. The checklist has 19 domains and includes reporting standards for economic model characteristics (population, time horizon, perspective and discount rate), identification and valuation of costs and outcomes, discussion points, conclusions as well as funding and conflicts of interest. All cost of illness studies were evaluated for risk of bias using the Larg and Moss's checklist. No quality appraisal was performed on studies reporting healthcare resource utilization.

Data Analysis

A component-based analysis was used to describe and synthesise the overall findings from all included studies. Specifically, tabulation methods were used to report on study characteristics, outcomes and costs. Tables for resource utilization and disaggregated costs were presented and summarized. All costs were presented according to the recommendations of Turner et al., 2019[13]. For studies that did not provide the year of cost data, the year of publication was used. Adjustment for inflation was done using the Gross Domestic Product deflator (GDP deflator) of the studied country. Cost estimates were then converted and reported in 2017 United States Dollars (USD). GDP deflator and exchange rates were obtained from the World Bank.[14]

Patient and public involvement

Patients were not involved in this systematic review. Their input was not sought in the design, interpretation or writing of the document.

For peer review only

RESULTS

Study Selection

Our search yielded a total of 11,443 articles of which 8,779 articles were excluded as they were not relevant for this review based on title screening. The remaining 2,664 articles were further screened by title and abstract and 299 articles were assessed for inclusion. We excluded 261 articles (n= 98 for not related to HSV, n =44 review articles/case report, n =116 not reporting resource utilization or cost, n =3 available only in abstract), leaving a total of 38 studies included in this review, as shown in Figure 1.

Overview of Study Characteristics

Of the 38 included articles, 14 studies[15-28] described resource utilization only, 12 studies[29-40] reported on costs, and 12 studies[41-52] reported both resource utilization and costs of HSV diagnosis/management. These studies, published from 1989 to 2020, reported resource utilization or costs related to the diagnosis and management of HSV-related GUD among adults/adolescents[18-22 28 30-34 37-40 44 52] (n=17), neonatal herpes prevention in pregnant mothers (n=13)[23-25 27 29 35 36 42 43 46-49] and neonatal herpes management[15-17 26 41 45 50 51] (n=8). The majority of studies were conducted in HIC (35/38, 94.6%) including the United States[15 17 20 22 25 27 29 30 34 35 38-52] (n= 26), Canada[18 19 26 36] (n=4), United Kingdom[23 33] (n=2), France[16 28] (n=2) and Ireland[24] (n=1)), while only one study (1/38, 2.6%) was conducted in a middle-income country, in particular South Africa[32]. A global survey focusing on the experiences of patients receiving care for genital herpes in 78 countries included some data on healthcare utilization.[21] In addition, a modelling study estimated the costs of implementing the Global Health Sector Strategy on Sexually Transmitted Infections (STIs), 2016-2021, in 117 LMICs, including costs related to syndromic management of GUD, the vast majority of which is caused by HSV-2.[37] The quality of included studies are summarised in Appendix Figure 1 and Figure 2.

Methodological Heterogeneity

There was substantial heterogeneity in the reporting of the included studies. Most studies were cost or resource utilization studies (n=23), while the remaining were cost-effectiveness studies (n=15). Among cost or resource utilization studies, data were collected retrospectively (n=13), prospectively (n=7), or not reported (n=7). The number of participants in each study varied, which could be as few

For peer review only

as 39 participants to as large as 42 million in studies that analysed claims datasets. Twenty-one studies (21/38, 55.3%) included participants who had either HSV-1 or 2, ten studies (10/37, 27.0%) specifically included participants with HSV-2, while the remaining eight studies (8/38, 21.1%) did not specify which type of HSV they examined. A summary of the characteristics of these studies is presented in Appendix Table 1, and study findings are presented in Appendix Tables 2 and Appendix Table 3 (See appendix for detailed unit cost tables and accompanying references).

Cost and healthcare resource utilization pattern of genital herpes infection

Among all 17 studies[18-22 28 30-34 37-40 44 52] investigating cost and healthcare resource utilization pattern of genital herpes, 11 studies reported some cost components of care for genital herpes infection[30-34 37-40 44 52] (Appendix Tables 1, 2 and 4). All but one of these studies were conducted in HIC and only one LMIC study (from South Africa) was found. The cost components of the included studies were variably reported. Three studies[31 34 52] reported laboratory testing costs associated with diagnosing HSV. Eight studies[30 31 33 34 37 40 44 52] described costs associated with syndromic management of GUD. In four studies[32 33 37 52], the authors describe the drug charges associated with treatment or prevention of HSV using oral acyclovir (doses of 200mg-400mg). The cost reported varied considerably, ranging between USD$0.53 to USD$16 for a 5 to 7 day treatment course for episodic GUD and USD$40 for a month of suppressive therapy with acyclovir. Two studies[31 44] provided the total drug charges associated with overall management of GUD, but no details related to the treatment regimen, duration or HSV of HSV being treated (Appendix Table 2). Seven studies[31-33 37 47 48 52] described labour and service delivery costs such as cost of physician visits, drug procurement cost, counselling cost and clinical examination associated with HSV. Similarly, there was variation in terms of reported labour and service delivery cost, which could be as low as USD$0.28 for 10-minute counselling[33] to as high as USD$120 for consultation and lost wages of patient time[52]. Indirect costs were considered only by Szucs *et al*, who estimated HSV-related productivity losses, which was estimated at USD$60 a visit[31].

Considering the cost components together, Owusu-Edusei *et al* estimated that the lifetime direct medical cost per case of genital HSV infection in the U.S. (considering only GUD-related costs and adjusted to 2017 USD) was USD$855 among men (range: USD$428- USD$1,284) and USD$698 among women (range: USD$350- USD$1,047)[30]. This translated to a total cost of USD$607.3 million (range: USD$303.59 million – USD$ 910.89 million in 2017 USD) for lifetime management of new or

newly diagnosed cases of HSV-2 in the United States occurring in 2008. Scuzs *et al* meanwhile estimated that the annual direct and indirect medical costs in the United States would amount to USD$983 million, based upon an estimated 3.1 million symptomatic genital HSV episodes (both new and recurrent) a year[31].

The only middle income country study, from South Africa[32], reported the diagnostic/ operational costs associated with medication, staff and laboratory costs for daily HSV-2 suppressive therapy among people living with HIV[32]. The median cost for HSV-2 suppressive therapy per life-year gained ranged between USD $685 to USD $951 (adjusted to 2017 dollar) among HIV-1 infected anti-retroviral naïve women. The authors estimated that this could be a cost-effective method for delaying HIV disease progression, especially when the price of acyclovir was lower than the price of USD $0.026/day for a twice daily 400mg dose. However, this study was conducted when ART use was recommended only when CD4 count fell below a threshold of <200 cells/μL or <350 cell/μL (Appendix Table 5). On a more global level, in Korenromp *et al*'s cost estimates for implementing the Global STI Strategy in 117 LMIC over 2016 to 2021, the authors reported that it would cost approximately USD$109 million to diagnose and treat HSV-related GUD episodes seen in clinical care, not including service delivery costs.[37] These costs were estimated despite assuming that only about 4% of all HSV-2 infected people would seek care for GUD (15% recognizing symptoms and 28% of those seeking care).

A total of 8 studies described healthcare resource utilization patterns for genital herpes infection[18-22 31 40 44], and all were from high income countries (Appendix Tables 1 and 3). Five of these studies[18 20-22 40] reported the population rate of seeking medical care for HSV, based upon retrospective analyses of databases of patients from health surveys[20-22]. In the study by Xia and colleagues, the authors found that the total genital herpes associated ED use increased from 24,747 visits in 2006 to 36,518 in 2013[40]. It is important to note that none of the studies reported the proportion of those seeking medical care among HSV-infected individuals. Most of these consultations were relatively short in nature, and were less than 15 minutes (79%)[21]. Two studies described the diagnostic methods used to determine HSV among their population. In the first study conducted in 2004, Patrick *et al.* surveyed physicians in 78 countries and reported that the most commonly used test was viral culture, which was performed in 49% of the individuals[21] (Appendix Table 3). A recent study in France by Heggarty *et al.* in 2020 found that 43.3% of respondents in their survey stated that they

For peer review only

would conduct PCR plus HSV serology and another 39.9% would conduct PCR only to confirm a HSV diagnosis[28].

Treatment patterns of individuals with genital herpes were also reported in four studies[19 21 28 44]. The study by DesHarnais *et al* in 1996 reported on antiviral use only among hospitalized patients with herpes infections, which is unlikely to be representative of the vast majority of people with HSV infection. Patrick *et al* in their survey found that 65% of people with genital herpes had ever been treated with antivirals, while 18% used topical prescription medication and 13% used over the counter topical cream. Among these individuals, 67% had received episodic therapy while 31% received chronic suppressive therapy (Appendix Table 2). Another study on herpes-related quality of life reported that 76.9% of respondents had ever been treated with antivirals, and 33.3% of the respondents with HSV were on suppressive antiviral therapy when the survey was administered[19].

Cost and healthcare resource utilization pattern of prevention of neonatal herpes among pregnant mothers

Nine studies reported costs for neonatal herpes prevention among pregnant mothers[29 35 36 42 43 46-49] (Appendix Tables 1, 2 and 6). Seven studies[35 36 42 43 46 47 49] provided estimates on the cost for treatment and childbirth delivery options, including caesarean and vaginal delivery in addition to inpatient costs. The cost of hospitalisation ranged considerably, and could be as low as USD$300 to as high as USD$32,483, while the cost of delivery ranged between USD$2,300 -$9,490. The costs associated with different laboratory tests used, such as ELISA screening or viral cultures[36 43] were reported, while detailed listing of the cost component of different delivery methods and hospital care were included in some studies (Appendix Table 6). The cost-effectiveness studies examined the impact of either acyclovir suppressive therapy[29 35 46 47] or routine antenatal screening[36 42 43 48 49] for prevention of neonatal herpes. In a study by Randolph *et al* in 1996[47], the authors found that prophylaxis with acyclovir during late pregnancy could be a cost-effective strategy to reduce the need for caesarean delivery due to genital herpes outbreaks during labour. Baker and colleagues in 2004 further expanded this work and estimated that adding serological testing to antiviral suppressive therapy had an incremental cost per quality-adjusted life year gained (QALY) of $18,680, compared with no screening or suppressive therapy[42]. A modelling study by Tuite *et al* in 2010 had similar findings related to screening for HSV in pregnancy[36].

For peer review only - http://bmjopen.bmj.com/site/about/guidelines.xhtml

Our focused search found a total of 10 studies which reported resource utilization among pregnant mothers to prevent neonatal herpes[23-28 42 43 46 48]. Among these, four were cost-effectiveness studies which had provided some information regarding resource utilization based upon estimates from literature or assumptions.[42 43 46 48] In one of the earliest studies by Brocklehurst in 1995, a survey of British obstetrician-gynaecologists revealed that most would recommend some form of antenatal screening for HSV using viral cultures usually by week 34 of gestation[23]. However, such screening is no longer recommended in the UK. Studies within HICs that have national obstetrics guidelines recommending caesarean delivery when HSV lesions are present at delivery have shown that most clinicians follow this guidance[24-27]. For example, in a Canadian study, caesarean section was offered "most of the time" to women with HSV lesions at delivery by 92% of obstetricians and 82% of family physicians[26]. In addition, in these settings women with genital herpes are often offered antiviral suppressive therapy in the third trimester[24 26]. Both valacyclovir and acyclovir have been used, with difference in preference by country. In the most recent survey of clinicians managing pregnant women with HSV by Heggarty *et al* in 2020, the authors noted that 68.4% "always" prescribe suppressive antiviral therapy during the third trimester and an additional 11.6% "often" prescribe it for women with symptomatic primary HSV infection during pregnancy.[25] For women with recurrent symptoms during pregnancy, 55.1% of providers "always" prescribe and 12.9% "often" prescribe antiviral prophylaxis in the third trimester.[28]

Cost and healthcare resource utilization pattern of neonatal herpes management

Four studies[41 45 50 51] reported cost of neonatal herpes management and reported only direct medical costs (Appendix Tables 1 and 2). One study reported direct non-medical cost for long-term care of individuals with neurological disability due to sequelae of HSV[43]. All studies were in HIC. The reported cost of hospitalisation of neonatal HSV ranged considerably, from S27,843 to $92,664. One study reported the cost associated with hospital readmission, which was reportedly similar to the first hospitalisation episode[50]. Six studies[36 46-49 52] accounted for the costs of informal care in their calculation. Informal caregiving was defined as care provided by caregivers for infants who had neurological sequelae following neonatal herpes. In total, seven studies[36 43 46-49 52] estimated long-term care costs of neonatal herpes patients. One of these, by Thung *et al*[49], provided the estimated cost for long term care of neonates with mild neurological deficit due to HSV, which cost USD$17,304.61 after adjusting for inflation to 2017 values. Six studies[43 46-49 52] provided estimates for the lifetime cost of caring for a child with moderate and severe disability, and fall within the range

For peer review only

USD$68,894 to USD$432,263 and USD$232,698 to USD$ 1,296,792 respectively. It is important to note that all studies relied on estimation of long-term costs calculated by Weitzman[53] with some different assumptions, while one study[43] used other sources of data.

A total of 7 studies[15-17 41 45 50 51] described resource utilization among individuals with neonatal herpes (Appendix Tables 1 and 3). These studies described the length of stay for hospitalization which varied considerably, with median hospital stays ranging from 6-34 days[15 16]. Ahmad *et al* noted that nearly 9.4 to 9.8% of neonates who had HSV required ICU stay[15]. None of the studies reported the number of days for ICU hospitalization.

For peer review only

DISCUSSION

Our review revealed a heterogeneous body of evidence on the healthcare resource utilization and costs associated with genital and neonatal HSV infection, as well as some summary economic estimates and cost-effectiveness studies of HSV intervention strategies, such as use of antivirals or screening, which included unit cost data. While the evidence base provides a starting point for understanding, several gaps remain. Despite the broad search strategy and inclusion criteria, we identified only 38 papers, which shows the paucity of data on HSV-related healthcare resource utilization as well as economic costs, especially from LMIC settings. The lack of data from LMIC is particularly concerning, as these countries bear the greatest burden of HSV infection and disease.[2 3 54] The current review only identified one cost-effectiveness analysis from a middle income country[32] focused on people living with HIV only, and one high-level modelling study predicting costs of implementing care for HSV GUD across 117 LMIC globally[37]. In addition, many of the studies we found were relatively old and may not reflect current practices such as the use of newer diagnostics (e.g. PCR) and newer care recommendations. For example, the global study by Patrick *et al.* reported that viral culture was the most common test used to diagnose HSV but this is likely because the use of PCR was not yet common in clinical practice at the time of the study. The 2020 study in France by Heggarty *et al.* reveals that PCR is now the most commonly used test, at least in this HIC setting, with and without HSV serology[28].

While data on resource utilization and costing were most comprehensive from the US, large gaps remain in many areas. For example, Gilbert and colleagues[20] described the proportions of individuals seeking care for genital herpes among adults aged 18 to 24 from 2000 to 2006, but since then there have been no new updates. In terms of costing, we noticed similar trends, as studies[30] mostly referenced cost data collected in 2001 by Szucs *et al*[31]. This lack of data is similarly noted related to HSV infection during pregnancy. While some information from health surveys exists, healthcare resource utilization information is rarely tracked or reported. Our search demonstrated that for most of the world, data on HSV related resource utilization are sparse. As such, new data sources and better data collection efforts are needed to collect these standardized non-fatal data from diverse healthcare settings. One major need is an understanding of how closely clinicians follow national guidelines on HSV care and treatment, such as the studies by Kenny *et al*[26] and Heggarty *et*[28] *al* from Canada and France respectively. For example, while there are structured guidelines for the workup of neonatal herpes and its related management, our review did not identify any studies

For peer review only

that described the compliance to these guidelines. Such information can provide us with vital clues into the economic burden of neonatal HSV as there is substantial cost due to the high mortality rates neonatal HSV was not treated.

Our review was also constrained in summarizing findings across studies or countries and in conducting across-study comparisons, due to the limited data and differing methodologies, healthcare settings, and practices, particularly for healthcare resource utilization. Another concern was the heterogeneity in data presentation in many studies identified. For example, the length of hospital stay reported in studies varied considerably, with different assumptions used by authors, and as a result, the cost of hospitalisation varied significantly even within the United States, which limits the potential generalizability of these findings across different settings[16 41 45 51]. Healthcare practices also differ between LMIC and HIC with respect to how HSV is managed, e.g., most HSV cases in LMICs are treated as part of syndromic management for GUD, without diagnostic testing. This may mean that additional testing costs might need to be considered for HICs, whereas additional treatment, for example for syphilis and chancroid, which can also cause GUD syndromes, might need to be considered for LMICs. The focus on GUD more generally in LMICs may have made it more challenging to identify potentially relevant HSV-specific studies for LMIC settings.

In order to estimate the global economic burden of HSV to contribute to the understanding of the potential value of HSV interventions, research on HSV-related costs and healthcare utilization patterns is urgently needed, especially from LMIC settings. Standardization of methods for the measurement and reporting of economic costs would enhance across-study comparisons and inform prioritization strategies of global funders. Only one study broadly attempted to quantify the economic burden of HSV, which the authors estimated would require a projected investment of around USD$109 million from 2016 to 2021, just for the management of HSV-associated GUD, not considering service delivery costs[37]. However, this analysis only modelled treatment of HSV GUD for a small proportion of people with HSV-2 infection (approximately 4%: assuming 15% would recognize symptoms and 28% of those would seek care) and did not account for HSV recurrences within a given year. New global estimates of HSV GUD suggest this is likely an underestimate.[3] In addition, as this model lacked country-level estimates of baseline disease and did not take into account the full spectrum of disease outcomes related to HSV nor the burden on health systems, the costing estimates remain imprecise and incomplete, suggesting the need for a more comprehensive model.

For peer review only

This is the first systematic review of scientific literature on the healthcare resource utilization for HSV. We conducted a comprehensive literature search and included grey literature through our focused search. Nevertheless, most studies were only conducted in HIC especially from the USA. As the practice and thus utilization of resources will vary between settings and countries due to epidemiological and health systems differences, this will limit the generalisability of findings. Nevertheless, results of this study will serve as a future repository for studies that wish to examine the economic evaluations of any public health interventions for HSV. This review also highlights the importance and need for more studies to describe on the healthcare resource utilization and associated cost of HSV, especially from LMIC. We assessed study quality of all included studies, which allows readers to assess the internal validity of these studies. The literature search was also limited to studies published in English language. As data on healthcare resource utilization may be published in government reports, or book chapters, these may not have been retrieved and included in this review, which may partly explain the lack of studies describing healthcare resource utilization from LMIC.

For peer review only

CONCLUSION

This review is the first attempt and a key step towards providing data needed to understand the global economic burden of HSV infection, for both HICs and LMICs. Available economic estimates, primarily from HICs, suggest the economic burden of HSV infection could be substantial. However, the global picture remains incomplete. Nevertheless, results obtained from this study will form a repository which can inform future economic evaluations of interventions for HSV infection, including HSV vaccines, microbicides, or new antiviral medications.[55] These types of economic data are crucial not only to improve the planning and development of any future HSV-related healthcare interventions, but also to optimize the allocation of healthcare expenditures and medical resources.

For peer review only

Contributors

SWHL served as the lead author, conducted the research, conducted the analyses, integrated the input from all team members and drafted the initial manuscript. SG directed the initial research and contributed to the initial draft, integrated her view points and served as an expert in this work.. NC conducted the research, mediated the discussion and helped refine the draft. All authors approved the final manuscript.

Data sharing statement

All relevant information and data have been presented in this article and its accompanying supplement.

Competing interest

All authors have no competing interest to report

Ethics Statement

This systematic review did not require ethical approval.

For peer review only

Figure legend

Figure 1. Flow diagram of study selection process

For peer review only

References

1. Gupta R, Warren T, Wald A. Genital herpes. *The Lancet* 2007;370(9605):2127-37. doi: 10.1016/S0140-6736(07)61908-4
2. James C, Harfouche M, Welton N, et al. Herpes simplex virus: global infection prevalence and incidence estimates, 2016. *Bulletin of the World Health Organization* 2020;98:315-29. doi: 10.2471/BLT.19.237149
3. Looker KJ, Johnston C, Welton NJ, et al. The global and regional burden of genital ulcer disease due to herpes simplex virus: a natural history modelling study. *BMJ global health* 2020;5(3):e001875.
4. Pinninti SG, Kimberlin DW. Maternal and neonatal herpes simplex virus infections. *American journal of perinatology* 2013;30(02):113-20.
5. Looker KJ, Welton NJ, Sabin KM, et al. Global and regional estimates of the contribution of herpes simplex virus type 2 infection to HIV incidence: a population attributable fraction analysis using published epidemiological data. *Lancet Infect Dis* 2020;20(2):240-49. doi: 10.1016/s1473-3099(19)30470-0 [published Online First: 2019/11/23]
6. Gottlieb SL, Low N, Newman LM, et al. Toward global prevention of sexually transmitted infections (STIs): the need for STI vaccines. *Vaccine* 2014;32(14):1527-35.
7. Gottlieb SL, Giersing BK, Hickling J, et al. Meeting report: Initial World Health Organization consultation on herpes simplex virus (HSV) vaccine preferred product characteristics, March 2017. *Vaccine* 2019;37(50):7408-18.
8. Gottlieb SL, Deal CD, Giersing B, et al. The global roadmap for advancing development of vaccines against sexually transmitted infections: Update and next steps. *Vaccine* 2016;34(26):2939-47. doi: https://doi.org/10.1016/j.vaccine.2016.03.111
9. Freeman EE, White RG, Bakker R, et al. Population-level effect of potential HSV2 prophylactic vaccines on HIV incidence in sub-Saharan Africa. *Vaccine* 2009;27(6):940-46. doi: https://doi.org/10.1016/j.vaccine.2008.11.074
10. Belshe RB, Heineman TC, Bernstein DI, et al. Correlate of immune protection against HSV-1 genital disease in vaccinated women. *J Infect Dis* 2014;209(6):828-36. doi: 10.1093/infdis/jit651 [published Online First: 2013/11/29]
11. Cochrane handbook of systematic review of interventions. 6.2 ed, 2021.
12. Page MJ, McKenzie JE, Bossuyt PM, et al. The PRISMA 2020 statement: an updated guideline for reporting systematic reviews. *BMJ* 2021;372:n71. doi: 10.1136/bmj.n71
13. Turner HC, Lauer JA, Tran BX, et al. Adjusting for Inflation and Currency Changes Within Health Economic Studies. *Value in Health* 2019
14. The World Bank. GDP deflator: linked series (annual %) 2019 2019 [Available from: https://data.worldbank.org/indicator/NY.GDP.DEFL.KD.ZG.AD accessed 25 November 2019.
15. Ahmad FA, Storch GA, Miller AS. Impact of an institutional guideline on the care of neonates at risk for herpes simplex virus in the emergency department. *Pediatric emergency care* 2017;33(6):396-401.
16. Bernard S, Mailles A, Stahl J. Epidemiology of infectious encephalitis, differences between a prospective study and hospital discharge data. *Epidemiology & Infection* 2013;141(11):2256-68.
17. Owusu-Edusei Jr K, Flagg EW, Gift TL. Hospitalization cost per case of neonatal herpes simplex virus infection from claims data. *Journal of pediatric nursing* 2015;30(2):346-52.
18. Aslam M, Kropp RY, Jayaraman G, et al. Genital herpes in Canada: Deciphering the hidden epidemic. *Canadian Journal of Infectious Diseases and Medical Microbiology* 2012;23(1):e6-e9.

For peer review only

19. Fisman D. Health related quality of life in genital herpes: a pilot comparison of measures. *Sexually transmitted infections* 2005;81(3):267-70.
20. Gilbert LK, Levandowski BA, Roberts CM. Characteristics associated with genital herpes testing among young adults: assessing factors from two national data sets. *Journal of American College Health* 2010;59(3):143-50.
21. Patrick D, Rosenthal S, Stanberry L, et al. Patient satisfaction with care for genital herpes: Insights from a global survey. *Sexually transmitted infections* 2004;80(3):192-97.
22. Tao G, Kassler WJ, Rein DB. Medical care expenditures for genital herpes in the United States. *Sexually transmitted diseases* 2000;27(1):32-38.
23. Brocklehurst P, Carney O, Ross E, et al. The management of recurrent genital herpes infection in pregnancy: A postal survey of obstetric practice. *British Journal of Obstetrics and Gynaecology* 1995;102(10):791-97.
24. Lynn N, Murphy S, Cunningham O, et al. HSV management in pregnancy at a joint antenatal-genitourinary clinic in a large maternity hospital in Dublin, Ireland-a model of care. *Sexually Transmitted Infections* 2017;93 (Supplement 1):A91.
25. Stankiewicz Karita HC, Moss NJ, Laschansky E, et al. Invasive obstetric procedures and cesarean sections in women with known herpes simplex virus status during pregnancy. *Open Forum Infectious Diseases* 2017;4(4) doi: 10.1093/ofid/ofx248
26. Kenny K, Leung W, Stephanson K, et al. Clinical practice in prevention of neonatal HSV infection: A survey of obstetrical care providers in Alberta. *Journal of Obstetrics and Gynaecology Canada* 2013;35(2):131-37. doi: https://doi.org/10.1016/S1701-2163(15)31017-3
27. Brown ZA, Wald A, Morrow RA, et al. Effect of serologic status and cesarean delivery on transmission rates of herpes simplex virus from mother to infant. *Jama* 2003;289(2):203-09.
28. Heggarty E, Sibiude J, Mandelbrot L, et al. Genital herpes and pregnancy: Evaluating practices and knowledge of French health care providers. *Eur J Obstet Gynecol Reprod Biol* 2020;249:84-91. doi: 10.1016/j.ejogrb.2020.02.039 [published Online First: 2020/03/04]
29. Barnabas R, Carabin H, Garnett G. The potential role of suppressive therapy for sex partners in the prevention of neonatal herpes: a health economic analysis. *Sexually transmitted infections* 2002;78(6):425-29.
30. Owusu-Edusei Jr K, Chesson HW, Gift TL, et al. The estimated direct medical cost of selected sexually transmitted infections in the United States, 2008. *Sexually transmitted diseases* 2013b;40(3):197-201.
31. Szucs TD, Berger K, Fisman DN, et al. The estimated economic burden of genital herpes in the United States. An analysis using two costing approaches. *BMC infectious diseases* 2001;1(1):5.
32. Vickerman P, Devine A, Foss AM, et al. The cost-effectiveness of herpes simplex virus-2 suppressive therapy with daily aciclovir for delaying HIV disease progression among HIV-1-infected women in South Africa. *Sexually Transmitted Diseases* 2011;38(5):401-09. doi: 10.1097/OLQ.0b013e31820b8bc8
33. Vickerman P, Ndowa F, Mayaud P. Cost-effectiveness of incorporating episodic treatment for Herpes simplex virus type-2 (HSV-2) into the syndromic algorithm for genital ulcer disease. *Sexually Transmitted Infections* 2008;84:243-48.
34. Owusu-Edusei Jr K, Nguyen HT, Gift TL. Utilization and cost of diagnostic methods for sexually transmitted infection screening among insured American youth, 2008. *Sexually transmitted diseases* 2013a;40(5):354-61.
35. Scott LL, Alexander J. Cost-effectiveness of acyclovir suppression to prevent recurrent genital herpes in term pregnancy. *American journal of perinatology* 1998;15(01):57-62.
36. Tuite AR, McCabe CJ, Ku J, et al. Projected cost-savings with herpes simplex virus screening in pregnancy: towards a new screening paradigm. *Sexually transmitted infections* 2011;87(2):141-48.

For peer review only

37. Korenromp EL, Wi T, Resch S, et al. Costing of national STI program implementation for the global STI control strategy for the health sector, 2016-2021. *PloS one* 2017;12(1):e0170773.
38. Fisman DN, Lipsitch M, HOOK III EW, et al. Projection of the future dimensions and costs of the genital herpes simplex type 2 epidemic in the United States. *Sexually transmitted diseases* 2002;29(10):608-22.
39. Almonte-Vega L, Colón-Vargas M, Luna-Jarrín L, et al. Cost analysis of treatment strategies for the control of HSV–2 infection in the U.S.: A mathematical modeling-based case study. *Mathematical Biosciences* 2020;324:108347. doi: https://doi.org/10.1016/j.mbs.2020.108347
40. Xia F, Fuhlbrigge M, Dommasch E, et al. Cost of Routine Herpes Simplex Virus Infection Visits to US Emergency Departments 2006–2013. *Western Journal of Emergency Medicine* 2018;19(4):689.
41. Ambroggio L, Lorch SA, Mohamad Z, et al. Congenital anomalies and resource utilization in neonates infected with herpes simplex virus. *Sexually transmitted diseases* 2009;36(11):680.
42. Baker D, Brown Z, Hollier LM, et al. Cost-effectiveness of herpes simplex virus type 2 serologic testing and antiviral therapy in pregnancy. *Am J Obstet Gynecol* 2004;191(6):2074-84. doi: 10.1016/j.ajog.2004.05.034 [published Online First: 2004/12/14]
43. Binkin NJ, Koplan JP. The high cost and low efficacy of weekly viral cultures for pregnant women with recurrent genital herpes: a reappraisal. *Medical Decision Making* 1989;9(4):225-30.
44. DesHarnais S, Simpson KN, Paul JE. Variations in practice patterns: antiviral drug use in hospitalized patients with herpes infections. *American Journal of Medical Quality* 1996;11(1):33-42.
45. Donda K, Sharma M, Amponsah JK, et al. Trends in the incidence, mortality, and cost of neonatal herpes simplex virus hospitalizations in the United States from 2003 to 2014. *Journal of Perinatology* 2019;39(5):697-707. doi: 10.1038/s41372-019-0352-7
46. Little SE, Caughey AB. Acyclovir prophylaxis for pregnant women with a known history of herpes simplex virus: a cost-effectiveness analysis. *American journal of obstetrics and gynecology* 2005;193(3):1274-79.
47. Randolph AG, Hartshorn RM, Washington AE. Acyclovir prophylaxis in late pregnancy to prevent neonatal herpes: a cost-effectiveness analysis. *Obstetrics & Gynecology* 1996;88(4):603-10.
48. Rouse DJ, Stringer JS. An appraisal of screening for maternal type-specific herpes simplex virus antibodies to prevent neonatal herpes. *American journal of obstetrics and gynecology* 2000;183(2):400-06.
49. Thung SF, Grobman WA. The cost-effectiveness of routine antenatal screening for maternal herpes simplex virus–1 and–2 antibodies. *American journal of obstetrics and gynecology* 2005;192(2):483-88.
50. Mahant S, Hall M, Schondelmeyer AC, et al. Neonatal herpes simplex virus infection among Medicaid-enrolled children: 2009–2015. *Pediatrics* 2019;143(4):e20183233. doi: 10.1542/peds.2018-3233
51. Flagg EW, Weinstock H. Incidence of neonatal herpes simplex virus infections in the United States, 2006. *Pediatrics* 2011;127(1):e1-e8.
52. Fisman D, Hook E, Goldie S. Estimating the costs and benefits of screening monogamous, heterosexual couples for unrecognised infection with herpes simplex virus type 2. *Sexually transmitted infections* 2003;79(1):45-52.
53. Waitzman NJ, Romano PS, Scheffler RM. Estimates of the economic costs of birth defects. *Inquiry* 1994:188-205.
54. Looker KJ, Elmes JAR, Gottlieb SL, et al. Effect of HSV-2 infection on subsequent HIV acquisition: an updated systematic review and meta-analysis. *The Lancet Infectious diseases* 2017;17(12):1303-16. doi: 10.1016/S1473-3099(17)30405-X [published Online First: 08/23]

For peer review only

55. Gottlieb SL, Deal CD, Giersing B, et al. The global roadmap for advancing development of vaccines against sexually transmitted infections: update and next steps. *Vaccine* 2016;34(26):2939-47.

For peer review only

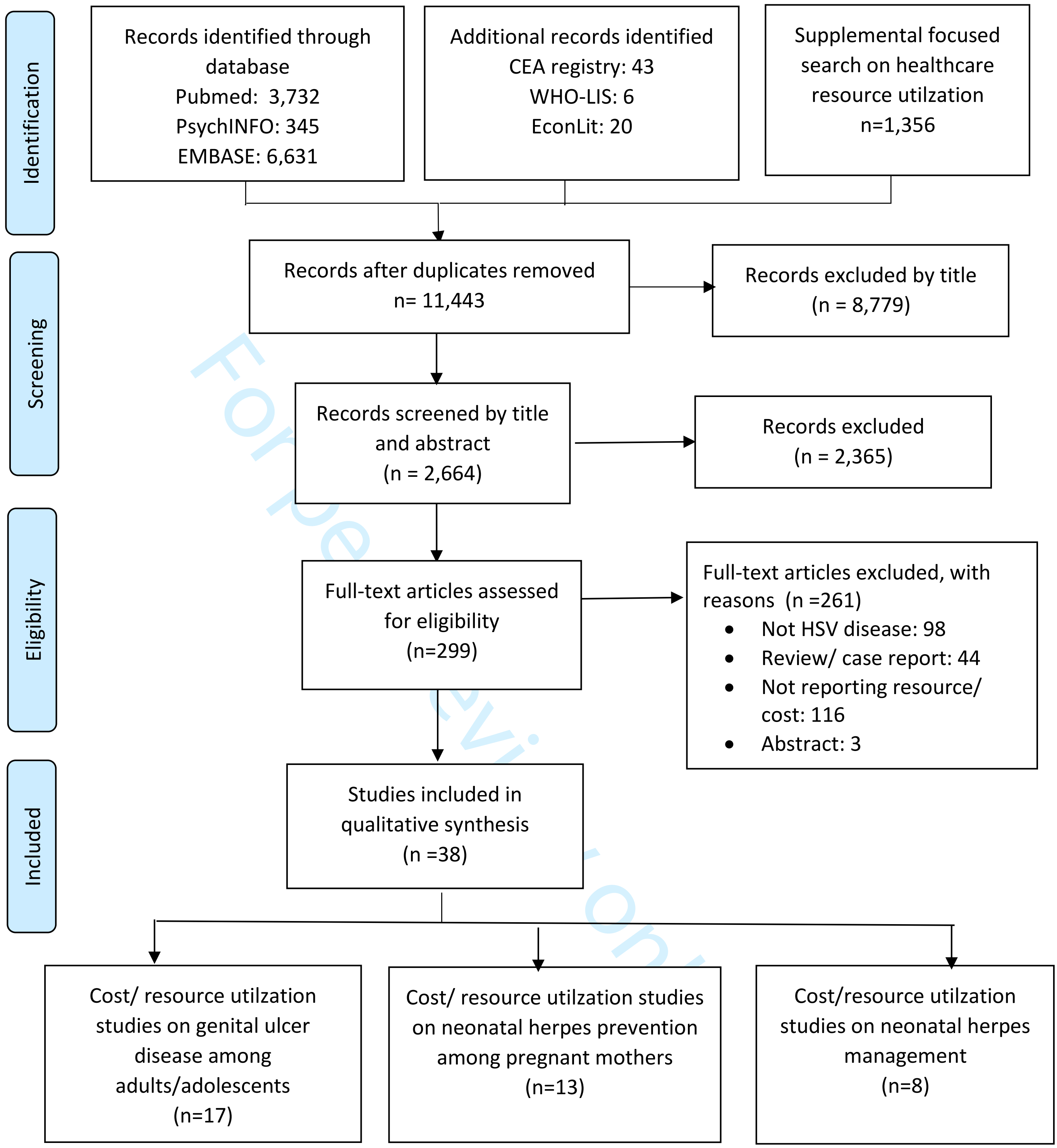
Identification
Screening
Eligibility
Included
Records identified through database
Pubmed: 3,732
PsychINFO: 345
EMBASE: 6,631
Additional records identified
CEA registry: 43
WHO-LIS: 6
EconLit: 20
Supplemental focused search on healthcare resource utilzation
n=1,356
Records after duplicates removed
n= 11,443
Records excluded by title
(n = 8,779)
Records screened by title and abstract
(n = 2,664)
Records excluded
(n = 2,365)
Full-text articles assessed for eligibility
(n=299)
Full-text articles excluded, with reasons (n =261)
Not HSV disease: 98
Review/ case report: 44
Not reporting resource/ cost: 116
Abstract: 3
Studies included in qualitative synthesis
(n =38)
Cost/ resource utilzation studies on genital ulcer disease among adults/adolescents
(n=17)
Cost/ resource utilzation studies on neonatal herpes prevention among pregnant mothers
(n=13)
Cost/resource utilzation studies on neonatal herpes management
(n=8)

APPENDIX

TABLE OF CONTENTS

Search methodology 2

Text 1: Keyword terms used in the search.. 3

Text 2: Keywords used in focused search using exploding terms.. 4

Table 1: Summary of included studies reporting healthcare costs and/or resource utilization related to HSV infection.. 5

Table 2: Detailed description of studies reporting cost (unit cost) 13

Table 3: Detailed description of studies reporting resource utilization 19

Table 4: Detailed cost incurred in genito-ulcer diseases due to HSV 22

Table 5: Detailed cost associated with genitoulcer disease prevention in people living with HIV 25

Table 6: Detailed cost associated with neonatal herpes prevention/management.................. 26

Figure 1: Methodological quality of included economic studies using CHEC Checklist............. 30

Figure 2: Methodological quality of included costing studies using Larg and Moss Checklist ... 31

For peer review only

Search methodology

1. Search strategy

- The current search strategy was developed based upon keywords which have been used in previous existing HSV reviews commissioned by WHO. All search keywords used were subsequently cross-checked with the following articles to ensure comprehensiveness

    - Looker, 2017. Effect of HSV-2 infection on subsequent HIV acquisition: an updated systematic review and meta-analysis
    - Khard, 2019. The Epidemiology of Herpes Simplex Virus Type 1 in Asia: Systematic Review, Meta-analyses, and Meta-regressions
    - Looker,2012. Global estimates of prevalent and incident herpes simplex virus type 2 infections in 2012. PLoS One 2015;10(1) : e114989-e89. Doi: 10.1371/journal.pone.0114989
- The following databases were identified for the search including: PubMed, PsychINFO, EMBASE, Centre for Review and Dissemination, EconLit, CEA registry and WHO Library Database (WHOLIS)

2. **Keywords search** was revised to compare between a) search including exploding terms and b) search including title and abstract. A total of 10,113 articles was found for search when terms were exploded versus 5,966 when these terms were not exploded. As such, the methods will only use search including exploding terms to minimize the risk of missing relevant study despite its low specificity. The initial search was performed in April 2019, with an updated search in October 2019.

3. Neonate search

- We also conducted search over again using all relevant HSV terms with neonate as keyword. All articles identified in the search overlapped with existing broader search, thereby there is no need to add neonate as key words

For peer review only

Text 1: Keyword terms used in the search

No.	Keyword
#1	Genital ulcer disease.mp.
#2	Herpes labialis.mp.
#3	Herpes genitalis.mp.
#4	Genital herpes.mp.
#5	Herpesvirus.mp.
#6	Herpes virus.mp.
#7	HSV.mp.
#8	Herpes simplex.mp.
#9	1 or 2 or 3 or 4 or 5 or 6 or 7 or 8
#10	Healthcare util*ation.mp.
#11	Util*ation.mp.
#12	Physician visit.mp.
#13	General practitioner visit.mp.
#14	Hospital visit.mp.
#15	Clinic visit.mp.
#16	Hospital stay.mp.
#17	Hospitali*ation.mp.
#18	Hospital readmission.mp.
#19	Cost.mp.
#20	Cost-effectiveness.mp.
#21	Cost-utility.mp.
#22	Cost-benefit.mp.
#23	Cost-minimi*ation.mp.
#24	Counselling.mp.
#25	Seek care.mp.
#26	Behavio*r.mp.
#27	10 or 11 or 12 or 13 or 14 or 15 or 16 or 17 or 18 or 19 or 20 or 21 or 22 or 23 or 24 or 25 or 26
#28	9 and 27

For peer review only

Text 2: Keywords used in focused search using exploding terms.

No.	Keyword
#1	Genital ulcer disease.mp.
#2	Herpes labialis.mp.
#3	Herpes genitalis.mp.
#4	Genital herpes.mp.
#5	Herpesvirus.mp.
#6	Herpes virus.mp.
#7	HSV.mp.
#8	Herpes simplex.mp.
#9	1 or 2 or 3 or 4 or 5 or 6 or 7 or 8
#10	pregnancy.mp.
#11	pregnant.mp.
#12	c*esarean.mp.
#13	delivery.mp.
#14	10 or 11
#15	12 or 13
#16	9 AND 14 AND 15

For peer review only

Table 1: Summary of included studies reporting healthcare costs and/or resource utilization related to HSV infection

Author, year Country	Population and setting	Study design	Study objective	Sample size	HSV-subtype		Cost data		Healthcare resource utilization	
					1	2	Healthcare delivery process	Treatment	Healthcare delivery process	Treatment
**HSV genital ulcer disease among adults/adolescents**										
Almonte-Vega, 2020 USA[39]	General population aged 15-49 years old	Cost-analysis	To study the dynamics of HSV–2 transmission, control and impact of treatment policies	-		x		x		
Aslam, 2012 Canada[18]	Records of individuals in the Canadian Disease and Therapeutic Index (CDTI)	Retrospective study	To investigate the rates of diagnosed cases of GH in Canada from 2002 to 2007	652					x	
Desharnais, 1996 USA[44]	Adults with herpes diagnosis from the HCIA Clinical Pathways Data Base	Retrospective study	To describe patterns of antiviral drug use for patients hospitalized with chickenpox, herpes simplex, and herpes zoster infections, and also for a subgroup of herpes patients with severe infections (systemic infections, eye infections, encephalitis, hemorrhagic pneumonitis, and other severe conditions)	3011	x	x		x		x
Fisman, 2002 USA[38]	Individuals aged 15 to 39 years	Cost-effectiveness	To project the future burden of HSV-2 infection in the United States, using a	-		x	x	x		

For peer review only

			mathematical model that incorporated epidemiologic trends documented between 1976 and 1994							
Fisman, 2003 USA[52]	Heterosexual couples	Modelling study	To evaluate the projected cost effectiveness of strategies to prevent HSV-2 transmission in couples with no history of HSV-2 infection	-		x		x		
Fisman, 2005 Canada[19]	Individuals with recurrent genital ulcer	Prospective study	To estimate the impact on health-related quality of life associated with both symptomatic and asymptomatic GH	39	x	x				x
Gilbert, 2010 USA[20]	Young adults	Retrospective study	To investigate characteristics associated with GH screening and diagnosis in sexually active young adults aged 18 to 24	Add Health Data: 11,570 NCHA: 222,740	x	x			x	
Korenromp, 2017[37]	People 15-49 year old living with HSV-2	Modelling study	To estimate the costs of reaching the 2020 STI strategy milestones for the period 2016–2021, to support policy, planning, implementation, and future cost-benefit evaluation of the global STI strategy 2016–2021.	-		x	x	x		
Owusu-Edusei, 2013a USA[34]	People aged 15-25 years	Retrospective study	To examine the utilization and cost of the diagnostic methods used for STI screening among	-		x	x			

For peer review only

			privately insured adolescent and young adult population							
Owusu-Edusei, 2013b USA[30]	-	Cost of illness analysis	To update the estimates of lifetime direct medical cost for 8 major STI	-				x		
Patrick, 2004 Worldwide survey from 78 countries[21]	Subjects with genital herpes	Survey	To describe patient experiences and views regarding genital herpes management	2075	x	x			x	x
Szucs, 2001 USA[31]	General population	Economic analysis	To estimate the economic burden of GH in the USA, using two different costing approaches	465,07 5			x	x		
Tao, 2000 USA[22]	General population	Cost-of-illness analysis	To assess the US direct medical expenditures for genital herpes and its complications to assist policy makers in allocating limited STD resources efficiently	-		x			x	
Vickerman, 2008 UK[33]	-	Cost-effectiveness	To compare the cost per ulcer treated of using the 1994 and 2003 algorithms amongst individuals presenting with GUD	-		x		x		
Vickerman, 2011 South Africa[32]	HIV+ women	Cost-effectiveness	To estimate the cost-effectiveness of daily acyclovir for delaying HIV-1 disease progression in women not eligible for antiretroviral therapy (ART)	300		x		x		

For peer review only

Xia, 2018 United States[40]	General population	Retrospective study	To determine the utilization and cost burden associated with HSV infection visits to U.S. EDs in recent years from 2006-2013	704,728			x		x	
**Neonatal herpes prevention among pregnant women**										
Baker, 2004 USA[42]	-	Cost-effectiveness	To determine whether serologic testing for herpes simplex virus type 2 (HSV-2) in pregnant women and their partners is cost-effective	100,000		x		x	x	x
Barnabas, 2002[29] USA	-	Cost-effectiveness	To assess the potential effectiveness, cost effectiveness, and benefit of suppressive therapy among herpes simplex virus serodiscordant sex partners during pregnancy		x	x	x	x		
Binkin, 1989 USA[43]	Pregnant women with HSV	Cost-effectiveness	To present a reanalysis of the cost effectiveness of maternal herpes screening and a review of the changes that have occurred in the screening recommendations since 1980	3,600,000	x	x	x	x	x	
Brocklehurst, 1995 UK[23]	All members and Fellows of the Royal College of Obstetricians and Gynaecologist resident	Survey	To determine the clinical practice among obstetricians in the antepartum and intrapartum management of women with recurrent genital herpes infection	2252	x	x			x	x
Brown, 2003 USA[27]	Pregnant women from university,	Cohort study	To determine the effects of viral shedding, maternal HSV	58362	x	x				x

For peer review only

	army and community hospitals		serological status and delivery route on risk of transmission of HSV from mother to infant							
Heggarty, 2020 France[28]	Healthcare providers for pregnant women	Survey	To evaluate health care provider knowledge, and collect information on management of genital herpes during pregnancy and infants born to mothers with herpes	354	x	x			x	x
Kenny, 2013 Canada[26]	Obstetrician, gynaecologist and family physicians offering maternity care practicing in Alberta	Survey	To identify the practice patterns of physicians providing prenatal care in Alberta with respect to prevention of neonatal HSV infection, including their prescribing of antiviral therapy to pregnant women in the third trimester.	183	x	x			x	x
Little, 2005 USA[46]	Women with a history of diagnosed genital HSV	Cost-effectiveness	To determine the clinical benefits and cost-effectiveness of prophylactic acyclovir in women with a history of HSV but no recurrence during pregnancy	-	x	x		x		x
Lynn, 2017 Ireland[24]	Pregnant women with genital HSV from a university hospital	Antenatal chart review	To describe the HSV management in pregnancy at a joint antenatal genital maternity hospital	107	x	x			x	x
Randolph, 1996 USA[47]	Antenatal women with recurrent genital HSV	Cost-effectiveness	To compare the cost-effectiveness of oral acyclovir prophylaxis in late pregnancy compared to caesarean delivery for genital herpes	10,000			x	x		

For peer review only

			lesions in the prevention of neonatal herpes transmission from mothers with recurrent genital infections							
Rouse, 2000 USA[48]	Antenatal women	Cost-effectiveness	To evaluate the potential cost effectiveness of herpes simplex virus antibody screening	8,538	x	x	x	x	x	
Scott, 1998 USA[35]	-	Cost-effectiveness	To determine whether acyclovir suppression provides a greater cost savings over no medical therapy in the management of recurrent genital herpes (HSV) in pregnancy	-	x	x	x	x		
Stankiewicz Karita, 2017 USA[25]	Pregnant women from a hospital	Retrospective study	To investigate the frequency of invasive obstetric procedures and caesarean deliveries for women with known HSV infection	449		x			x	
Thung, 2005 USA[49]	Married women	Cost-effectiveness	To determine the cost-effectiveness of routine antenatal screening for HSV-1 and HSV-2 in women without a known history of genital herpes.	100,000	x	x		x		
Tuite, 2010 Canada[36]	Pregnant women	Cost-effectiveness	To assess the effectiveness and cost effectiveness of identifying pregnant women at risk of de novo HSV acquisition to prevent vertical HSV transmission	100,000	x	x	x	x		

For peer review only

**Neonatal herpes management**										
Ahmad, 2015 USA[15]	Neonates who sought care in emergency department	Retrospective study	To evaluate whether guideline implementation affected the ED's decision to test for HSV, ED use of HSV polymerase chain reaction (PCR) and acyclovir	308	x	x				x
Ambroggio, 2009 USA[41]	Neonates with HSV and received intravenous acyclovir and discharge from Paediatric Health Information System	Retrospective study	To quantify the economic burden of neonatal HSV during initial hospitalization while focusing on factors, such as congenital anomalies and HSV-associated complications, which increase hospital charges and length of hospital stay among neonates with HSV	406	x	x		x		x
Bernard, 2013 France[16]	Patients aged 28 days and above from the French national hospital discharge database	Prospective study	To compare the data from the French national hospital discharge database (Programme de Me´ dicalisation des Syste` mes d'Information; PMSI) and from the prospective study conducted in 2007 and evaluate the reliability of PMSI as a tool to assess the trends of encephalitis in France	1,947	x	x				x
Donda, 2019 USA[45]	Neonates with ICD-9 codes for neonatal HSV in the National	Retrospective study	To examine the temporal trends in the incidence and outcomes of neonatal HSV in the United States	42,726, 336				x		x

For peer review only

	Inpatient Sample from 2003-2014									
Flagg, 2011 USA[51]	Inpatient records of infants aged 60 days or younger from the Healthcare Cost and Utilization Project Kids' Inpatient Database	Retrospective study	To estimate the incidence of HSV infections for the United States during 2006, as well as demographic-specific rates, by using nationally and regionally weighted estimates from a population-based sample of inpatient data	4,106,488	x	x		x		x
Mahant, 2019 USA[50]	Records of neonates from the Medicaid claims database from 2009 - 2015	Retrospective study	To examine the incidence, mortality, and health care use related to neonatal herpes HSV infection.	2,107,124				x		x
Owusu-Edusei, 2015 USA[17]	Insurance claim data on inpatient admission from the Truven Health Analytics MarketScan Commercial Claims and Encounters Database	Cost-of-illness analysis	To estimate the average excess inpatient cost of neonatal herpes simplex virus (NHSV) infection from 2005 to 2009 insurance claims data	474,743	x	x				x

For peer review only

Table 2: Detailed description of studies reporting cost (unit cost)

Author, year Country	Population and setting	Diagnostic costs (range)	Treatment costs* in original year of value (range)	Hospitalisation costs (range)	Other healthcare delivery costs (range)	Lifetime management cost (range)
**Genital ulcer disease among adults/adolescents**						
Almonte-Vega, 2020 USA[39]	General population aged 15-49 years old	Microbiological lab test (unspecified): $80.17	Acyclovir treatment (duration not specified): $86.33	NR	Consultation, clinical examination and diagnostic: $161.85	NR
Desharnais, 1996[44]	Adults with herpes diagnosis identified from the HCIA database	NR	Total drug charges: $1941<br>Antiviral drug charges (not specified): $1070	Hospital charges: $5637	NR	NR
Fisman, 2002[38]	Individuals aged 15 to 39 years	NR	Cost of treatment for primary syndrome<br>Male: $470 ($370-5$60)<br>Female: $830 ($670-$1000)<br><br>Antiviral therapy<br>Relapse: $17 ($9-$36)<br>Monthly suppressive therapy: $40 ($20-$220)	NR	Clinic visit: $120 ($90-$150)<br>Obstetrical care: $310 ($130-$800)	Initial cost of caring for neonates with HSV: $42,600<br>Lifetime medical and long-term care cost for infants with moderate neurological sequalae: $97,000<br>Lifetime medical and long-term care cost for infants with severe neurological sequalae: $291,000

Fisman, 2003[52]	Heterosexual couples	Western blot: $60 ($45-$90)<br>ELISA: $5 ($3-$35)	Cost of treatment for primary syndrome<br>Male: $450 ($360-5$40)<br>Female: $800 ($640-$960)<br><br>Acyclovir (per episode): $16 ($9-$35)<br>Acyclovir (monthly suppressive): $40 ($20-$215)	NR	Clinic visit: $120 ($90-$145)<br>Labour: $120 ($90-$145)	Lifetime cost of care of neonatal HSV-2: $110,000 ($85,000-$860,000)
Korenromp, 2017[37]	People 15-49 year old living with HSV-2	NR	Acyclovir 400mg per tab: $0.04	NR	Treatment service delivery (not specified): $10<br>Procurement cost: $0.21	NR
Owusu-Edusei, 2013a[34]	People aged 15-25 years	Laboratory test (unspecified): $24.30-27.05	NR	NR	NR	NR
Owusu-Edusei, 2013b[30]	-	NR	NR	NR	NR	Lifetime medical cost per case, median(range):<br>Men: $761 (381-1,142)<br>Women: $621(311 - 932)<br>Lifetime cost of new infections acquired in 2008: $435.9 million

For peer review only

Szucs, 2001[31]	General population	Laboratory test: $1.5-76.50	Drug: $64-131	Hospitalisation: $669	Labour: $39.8 -62.6<br>Clinic visit: $36.20-73<br>Day off work: $144	NR
Vickerman, 2008[33]	-	NR	Acyclovir 200mg tds for 5 days: $0.53- 5.24	NR	Counselling cost: $0.28	NR
Vickerman, 2011[32]	HIV+ women	NR	Acyclovir 400mg: $0.07<br>Yearly ART cost: $1700 (1359-2000)	NR	Staff costs/women 3m treatment cycle: $15.60	NR
Xia, 2018[40]	General population	NR	NR	ED: $1,069		
**Neonatal herpes prevention among pregnant mothers**						
Baker, 2004[42]	-	Laboratory test with labor cost for HSV-2: 15.58 – 60.00	Average antiviral daily cost (assuming 50% on generic acyclovir 400mg tds and 50% on valacylovir qd): $1.70-7.90<br>Acyclovir 400mg: $0.366- 1.955<br>Valacyclovir 500mg/tab: $3.95<br>Valacyclovir 1g/tab: $6.49	Delivery: $4,779-22,838	Labour cost: $15.58 – $60<br>Counselling cost: $5.98-$6.67	Lifetime cost of care of neonatal HSV: $54,516- $129,576

For peer review only

Barnabas, 2002[29]	-	Diagnostic cost: $16-$100	Drug cost per couple per pregnancy: $37<br>Acute neonatal herpes treatment $1,500-50,000	C/S cost (personnel, supplies, surgery and ward care): $11,084	Labour cost: $200-1628<br>Counselling cost: $12-$19	Neonatal care after C/S: $884<br>Long term care for neonatal herpes: $140,766 - $273,712
Binkin, 1989[43]	Pregnant women with HSV	Viral culture: $30	NR	Hospitalisation for complication: $300-698<br>Hospital care associated with neonatal herpes: $25,000<br>Delivery: $2,300-3,600	NR	Long term care for neonatal herpes: $125,000-$250,000
Little, 2005[46]	Women with a history of diagnosed genital HSV	NR	Acyclovir (prophylaxis) from 36 weeks of gestation: $46	Delivery: $4,939-9,490<br>Hospitalisation: $32,483	NR	Lifetime cost of care of neonatal HSV: $349,7533-$1,049,260
Randolph, 1996[47]	Antenatal women with recurrent genital HSV	Laboratory: $35	Acyclovir 400mg (200caps): $228	Delivery: $3,500	Labour: $74	Lifetime cost of care of neonatal HSV: $85,000- 255,000
Rouse, 2000[48]	Antenatal women	Laboratory: $4 – 13	NR	Hospitalisation for neonatal care: $11,126	Labour: $3.50-10.50	Lifetime cost of care of neonatal HSV: $48,519- 163,879
Scott, 1998[35]	-	HSV culture: $80	Acyclovir 400mg tds for 4 weeks: $180	Hospitalisation for neonatal care: $480-1470<br>Delivery: $5,321 – 9,039	NR	NR

For peer review only - http://bmjopen.bmj.com/site/about/guidelines.xhtml

Thung, 2005[49]	Married women	HSV screening: $37.5-$75	Acyclovir 400mg tds for 4 weeks: $71	Delivery: $4,281 - 9,283	Counselling cost: $13	Lifetime cost of care of neonatal HSV: $13,202 – 325,602
Tuite, 2010[36]	Pregnant women	ELISA test: $7-$14	NR	Delivery: $5680- 8780	NR	Lifetime cost and consequence of neonatal HSV: $164,870
**Neonatal herpes management**						
Ambroggio, 2009[41]	Neonates with HSV and received intravenous acyclovir and discharge from Paediatric Health Information System	NR	Median pharmaceutical (not specified): $4,231 Median Imaging: $2,010	Median hospital charge: $37,431	NR	NR
Donda, 2019[45]	Patients aged 28 days and above from the French national hospital discharge database	NR	NR	Hospitalisation: $27,843	NR	NR
Flagg, 2011[51]	Neonates with ICD-9 codes for neonatal HSV	NR	NR	Hospitalisation: $92,664	NR	NR

For peer review only

	in the National Inpatient Sample from 2003-2014					
Mahant, 2019[50]	Records of neonates from the Medicaid claims database from 2009 - 2015	NR	NR	Hospitalisation: $32,683<br>Hospital readmission: $31,531<br>ED visit: $527	NR	NR

*All costs are mean costs except where explicitly labelled as median costs.

C/S – Caesarean section; ED - Emergency department; NR – Not reported

For peer review only

Table 3: Detailed description of studies reporting resource utilization

Author, year	Healthcare seeking and diagnosis	Treatment phase
**Genital ulcer disease among adults/adolescents**		
Aslam, 2012[18]	• 74.1-93.2% sought care once within 12 months<br>• 6.8-25.9% sought care twice to 8x a year	
Desharnais, 1996[44]		• Oral treatment only: 16.1%<br>• IV treatment: 16.2%<br>• Hospital stay: 5.4 days
Fisman, 2005[19]		• 33.3% used antiviral drugs for HSV<br>• 15.8% had pregnancy complicated by HSV
Gilbert, 2010[20]	• 1.32% of young adults ever tested for genital herpes	
Patrick, 2004[21]	• 49% had viral culture performed<br>• 9% had antibody test<br>• 34% had physical examination	• 65% received oral antiviral therapy<br>• 18% received topical antiviral therapy<br>• 17% obtained alternative therapy
Tao, 2000[22]	• Estimated annual genital herpes visit 499,655 yearly<br>• 2% were inpatient visit<br>• 9% outpatient & ED visit<br>• 20% public STD clinic<br>• 69% private office based visit	
Xia, 2018[40]	From 2006-2013<br>• 245,484 ED visits with primary diagnosis of genital herpes or 37.3% of total ED visits for HSV<br>• Total charges: $278,335,295<br><br>ED visits trend from 2006 – 2013<br>• 24,747 (33.8%); 26,440 (34.1%); 27,484 (36.1%), 28,440 (36.5%); 33,258 (37.8%); 33,095 (38.3%); 35,501 (40.0%); 36,518 (40.3%)	
**Neonatal herpes prevention among pregnant mothers**		
Baker, 2004[42]	Estimates used in model<br>• 75% of partners will be willing to undergo HSV screening	Estimates used in model<br>• 1.32% women HSV-2 negative acquiring HSV during last 8 weeks of pregnancy<br>• 57% women or partner offered and accept antiviral therapy with testing

For peer review only

		• 82% women taking antivirals from week 36 compliant
Binkin, 1989[43]	Estimates used in model<br>• Average number of cultures per patient: 8	
Brocklehurst, 1995[23]	• 60% of obstetricians advocated some form of antenatal screening<br>Among those performing screening<br>• 64% perform regular viral cultures<br>• 54% recommend screening ≤34 weeks of gestation	• 92% of providers: visible active lesions at labor are cause for caesarean delivery
Brown, 2003[27]		• All women with HSV genital lesions noted at delivery had caesarean delivery (n=60) unless lesions not noted until too late to proceed with caesarean or lesions noted after delivery (n=14)
Heggarty, 2020[28]	For suspected primary genital HSV:<br>▪ 43.3% would conduct PCR of lesions plus HSV serology<br>▪ 39.9% would conduct PCR of lesions alone<br>▪ 0.4% would conduct HSV serology only	• If primary HSV GUD during pregnancy, 68.4% "always" and 11.6% "often" prescribe antiviral prophylaxis in 3rd trimester<br>• If recurrent HSV GUD during pregnancy, 55.1% "always" and 12.9% "often" prescribe antiviral prophylaxis in 3rd trimester<br>• 83% recommend caesarean delivery if genital HSV lesions suspected during labour
Kenny, 2013[26]	• 30% physicians will perform type-specific serology "most of the time" for patients with no history of herpes but partner with known HSV	• Antiviral suppressive therapy prescribed in third trimester by 90% of doctors (97% of obstetricians and 84% family physicians)<br>▪ 62% prescribed for any past history of GUD including pre-pregnancy<br>▪ 28% only after outbreak during pregnancy<br>▪ More commonly prescribed acyclovir (63%) than valacyclovir (38%)<br>• 65% offer elective caesarean if primary HSV in third trimester<br>• 95% of obstetricians and 84% of family physicians recommend caesarean delivery if HSV lesions during labour
Little, 2005[46]		Estimates used in model<br>• 24% of women will undergo caesarean delivery if no lesion was present

For peer review only

Lynn, 2017[24]	• 89% of patients had type-specific serology sent	• 63% received antiviral prophylaxis<br>▪ 98.5% received valacyclovir<br>▪ 1.5% received acyclovir<br>▪ Mean for initiating: week 36<br>• 29% of patients underwent caesarean delivery, none for HSV
Rouse, 2000[48]	Estimates used in model<br>• 75% of partners will be willing to undergo HSV screening	
Stankiewicz Karita, 2017[25]		• Antiviral suppressive therapy:<br>▪ 55% HSV-2 antibody-positive only<br>▪ 65% history of symptomatic GUD<br>• Similar caesarean section rates for women with/without history of HSV/genital herpes:<br>▪ 25% without history of HSV-2/GH<br>▪ 30% on suppressive treatment<br>• 28% without suppressive treatment
**Neonatal herpes management**		
Ahmad, 2015[15]	• CSF PCR performed in 92.3%<br>• Blood PCR performed in 48.7%	• 9.4 – 9.8% require ICU stay<br>• Hospital stay: 83.1-84.6hr<br>• 71.8% received acyclovir
Ambroggio, 2009[41]		• Median length of stay: 13 days (IQR 4-21)
Bernard, 2013[16]		• Mean hospital admission: 28 -34 days
Donda, 2019[45]		• Median length of stay: 20
Flagg, 2011[51]		• Mean length of stay: 22 days<br>• Median length of stay: 2- days
Mahant, 2019[50]		• Median hospital stay: 18 days<br>• Post discharge,<br>○ 45.7% required ED visit<br>○ 16.2% required rehospitalisation
Owusu-Edusei, 2015[17]		• Mean hospital stay: 10.8 (11.5)<br>• Mean hospital stay among those with admission >7 days: 18.5 (12.5)

For peer review only

Table 4: Detailed cost incurred in genito-ulcer diseases due to HSV

Author,year	Outcomes	Unit cost ($) in original year	Unit cost in 2018 ($)
**Medication costs**			
Vickerman, 2008	One dose of IV benzathine penicillin 2.4MU	0.15 - 0.48	0.19-0.59
Vickerman, 2008	One tab of 500mg ciprofloxacin	0.10 - 0.21	0.12 - 0.26
Vickerman, 2008	One cap of 200mg acyclovir	0.53- 5.24	0.66 – 6.48
Fisman, 2003	Acyclovir therapy for relapse patients	16.00	22.72
Fisman, 2003	Acyclovir cost for suppressive monthly therapy	40.00	56.80
Almonte-Vega, 2020	Acyclovir therapy	86.33	86.33
Fisman, 2003	Condom cost	0.10	0.14
Szucs, 2001	Pharmacological treatment 1st episode (NS)	64.00	94.86
Szucs, 2001	Pharmacological treatment recurrent episode (NS)	131.00	194.18
Vickerman, 2008	Needle and syringe cost	0.15	0.19
Tao, 2000	Pharmacy claim	52.00	73.84
**Laboratory test**			
Szucs, 2001	Antibiotic testing based on expert opinion	76.50	113.39
Szucs, 2001	Antibiotic testing in first episode based on claims	12.80	18.97
Szucs, 2001	Antibiotic testing in subsequent episode based on claims	6.50	9.63
Szucs, 2001	Complete blood count based on expert opinion	21.29	31.56
Szucs, 2001	Complete blood count in first episode based on claims	4.60	6.82
Szucs, 2001	Complete blood count in subsequent episode based on claims	1.50	2.22
Szucs, 2001	Microbiological test for first GUD episode	17.60	26.09
Szucs, 2001	Microbiological test for subsequent GUD episode	6.70	9.93
Szucs, 2001	Microbiological test based on expert opinion	38.39	56.90
Almonte-Vega, 2020	Microbiological lab test	80.17	80.17

For peer review only

Fisman, 2003	Western blot	60.00	85.20
Szucs, 2001	Urine analysis based on expert opinion	12.59	18.66
Szucs, 2001	Urine analysis in first episode based on claims	4.60	6.82
Szucs, 2001	Urine analysis in subsequent episode based on claims	3.20	4.74
**Hospitalisation cost**			
Fisman, 2003	Excess obstetrical cost associated with history of symptomatic HSV2 infection	300.00	425.98
Fisman, 2003	Excess obstetrical cost due to symptomatic HSV2 infection	310.00	440.18
Tao, 2000	Inpatient cost	2,530.00	3592.46
Szucs, 2001	Hospital day	669.00	991.63
**Clinic visit**			
Fisman, 2003	Clinic visit related to GUD (for physician time, test, lost wages due to 2hr patient time)	120.00	170.39
Szucs, 2001	Clinical examination based on expert opinion	40.33	59.78
Szucs, 2001	Clinical examination first episode based on claims	39.80	58.99
Szucs, 2001	Clinical examination on subsequent episode based on claims	36.20	53.66
Szucs, 2001	Physician consultation based on expert opinion	73.00	108.21
Szucs, 2001	Physician consultation in first episode based on claims	62.60	92.79
Szucs, 2001	Physician consultation in subsequent episode based on claims	59.60	88.34
Tao, 2000	Outpatient and ED	59.00	83.78
Fisman, 2003	Outpatient visit	120.00	170.39
Tao, 2000	Office based physician and public clinic	67.00	95.14
Almonte-Vega, 2020	Consultation, clinical examination and diagnostic	161.85	161.85
Vickerman, 2008	Counselling cost	0.28	0.35
**Other costs**			
Szucs, 2001	Others miscellaneous cost related to first GUD episode(not reported)	33.00	48.91

For peer review only

Szucs, 2001	Others miscellaneous cost related to recurrent GUD episode(not reported)	12.30	18.23
Szucs, 2001	Production losses	60.00	88.94
Szucs, 2001	Total cost of active GUD	355.00	526.20
Szucs, 2001	Total cost of incident GUD	235.00	348.33
Szucs, 2001	Total cost of prevalent GUD	166.00	246.06
Szucs, 2001	Total cost of recurrent GUD	499.00	739.65
Fisman, 2003	Treatment cost for men assuming 2 clinic visit, 7 day course of acyclovir (400mg tds) and 2 days off work	450.00	638.97
Fisman, 2003	Treatment cost for women assuming 2 clinic visit, 7 day course of acyclovir (400mg tds) and 2 days off work	800.00	1135.95

For peer review only

Table 5: Detailed cost associated with genitoulcer disease prevention in people living with HIV

Author, year	Outcomes	Unit cost ($) in original year	Unit cost in 2018 ($)
Vickerman, 2011	Acyclovir 400mg	0.07	0.07
Vickerman, 2011	Staff cost- for default tracer over 3 months	24.00	22.32
Vickerman, 2011	Staff cost for training for STI diagnosis and default tracer	0.46	0.43
Vickerman, 2011	Labour cost for senior nurse	2.52	2.34
Vickerman, 2011	Counselling cost (10 mins)	0.88	0.82
Vickerman, 2011	CD-4 count test	7.90	7.35

NB- Cost reported after adjustment in 2017 were lower than those in the original study due to exchange rates at the time of study.

For peer review only

Table 6: Detailed cost associated with neonatal herpes prevention/management

Author,year	Outcomes	Unit cost (USD$)	Unit cost in 2017 ($)
**Medication costs**			
Randolph, 1996	One cap of acyclovir 400mg	1.14	1.72
Baker, 2004	Pharmaceutical cost for pregnant women	6.18	8.10
Baker, 2004	Pharmaceutical cost for partner	3.93	5.15
Baker, 2004	Valacyclovir 500mg	3.95	5.18
Baker, 2004	Valacyclovir 1000mg	6.49	8.51
Baker, 2004	Acyclovir 400mg	1.96	2.57
Barnabas, 2002	Acyclovir treatment for a couple for one pregnancy	37.00	51.37
Scott, 1998	Acyclovir 400mg	1.71	2.58
**Laboratory test**			
Randolph, 1996	Screening using herpes culture	35.00	52.83
Thung, 2005	HSV1 or 2 screening cost	37.50	49.15
Thung, 2005	HSV 1 and 2 screening	75.00	98.31
Rouse, 2000	HSV-2 antibody assay	4.00	5.68
Rouse, 2000	HSV-2 labour and reagent cost, QC etc	9.00	12.78
Tuite, 2010	ELISA screening for HSV	7.00	7.96
Scott, 1998	HSV culture	80.00	120.75
Baker, 2004	Labor and supplies for HSV-2 specific test	15.58	20.42
Baker, 2004	HSV test for partner	40.53	53.12
Barnabas, 2002	Diagnostic kit cost	70.00	97.18
Binkin, 1989	Viral culture	30.00	52.97
**Hospitalisation cost**			
Scott, 1998	Vaginal delivery with metritis, includes labour, delivery, postpartum and professional	8439.00	12,737.15

For peer review only

Scott, 1998	Vaginal delivery without metritis, includes labour, delivery, postpartum and professional	5,321.00	8,031.09
Ambroggio, 2009	Hospital charges	62,050.90	70,544.69
Tuite, 2010	Vaginal delivery	5,680.00	6,457.50
Little, 2005	Vaginal delivery	4,939.00	6,104.17
Randolph, 1996	Caesarean delivery over vaginal	3,500.00	5,282.62
Tuite, 2010	Caesarean section	8,780.00	9,981.84
Tao, 1999	Caesarean attributable to genital herpes	1,922.00	2729.13
Little, 2005	Caesarean delivery	9,490.00	11,728.80
Little, 2005	Caesarean delivery with lesion	7,608.00	9,402.82
Scott, 1998	Caesarean delivery with metritis, includes labour, delivery, postpartum and professional	9,039.00	13,642.74
Scott, 1998	Caesarean delivery without metritis, includes labour, delivery, postpartum and professional	10,553.00	15,927.85
Thung, 2005	Elective caesarean	7,425.00	9,732.37
Thung, 2005	Labour caesarean	9,283.00	12,167.75
Little, 2005	Hospital care due to neonatal herpes infection	32,483.00	40,146.12
Rouse, 2000	Hospital care due to neonatal herpes infection	11,126.00	15,798.28
Baker, 2004	Caesarean delivery	5,021.00	6,581.31
Binkin, 1989	Hospital stay due to complication	698.00	1,232.38
Binkin, 1989	Hospital care due to neonatal herpes infection	25,000.00	44,139.53
Barnabas, 2002	Caesarean delivery with lesion	11,084.00	15,388.48
**Clinic visit**			
Scott, 1998	Clinic visit	39.50	59.62
Thung, 2005	Counselling cost	13.00	17.04
Rouse, 2000	Counselling cost (10 mins)	3.50	4.97
Rouse, 2000	Counselling cost for couple (30 mins)	10.50	14.91
Randolph, 1996	Follow-up call and office visit following screening	74.00	111.69
Barnabas, 2002	Pharmacy dispensing and education cost	3.00	4.17
Barnabas, 2002	Obstetrician counselling and testing salary for screening	19.00	26.38

For peer review only

Barnabas, 2002	Obstetrician counselling and testing salary for treatment	12.00	16.66
**Long-term care cost**			
Scott, 1998	Infant treated for HSV (include drug and culture)	1,470.00	2,218.70
Scott, 1998	Neonatal care if using caesarean delivery	821.00	1,239.15
Scott, 1998	Neonatal care if using vaginal delivery	480.00	724.47
Randolph, 1996	Neonatal herpes acute hospital care	10,160.00	15,334.69
Thung, 2005	Acute and long term care for normal/mild deficit	13,202.00	17,304.61
Randolph, 1996	Long term medical cost for moderate disability (Y1-Y65)	85,000.00	128,292.20
Thung, 2005	Acute and long term care for moderate deficit	134,202.00	175,906.12
Little, 2005	Lifetime cost and care for moderately disabled child	349,753.00	432,263.77
Rouse, 2000	Lifetime cost and care for moderately disabled child 1999	48,519.00	68,894.21
Baker, 2004	Lifetime medical and institutionalised cost for neonatal herpes	92,350.00	121,048.35
Binkin, 1989	Lifetime cost and care for moderately disabled child	125,000.00	220,697.66
Fisman, 2003	Lifetime cost of neonatal HSV with moderate neurological sequel	97,000.00	13,7734.46
Randolph, 1996	Long term medical cost for severe disability (Y1-Y65)	255,000.00	384,876.59
Thung, 2005	Acute and long term care for severe deficit	325,602.00	426,784.88
Little, 2005	Lifetime cost and care for severely disabled child	1,049,260.00	1,296,792.56
Rouse, 2000	Lifetime cost and care for severely disabled child	163,879.00	232,698.82
Binkin, 1989	Lifetime cost and care for severely disabled child	250,000.00	441,395.33
Fisman, 2003	Lifetime cost of neonatal HSV with severe neurological sequel	291,000.00	413,203.38
Tuite, 2010	Lifetime cost of neonatal HSV	164,870.00	187,438.10
Fisman, 2003	Lifetime cost of neonatal HSV	110,000.0	156,193.72
Baker, 2004	Counselling cost nurse (15 mins)	5.98	7.84
Baker, 2004	Counselling cost physician (5 mins)	6.67	8.74
Baker, 2004	Labour cost and supplies	15.58	20.42
Baker, 2004	Total cost without screening program	1,181.35	1,548.46
Baker, 2004	Total cost with screening for women	1,211.95	1,588.57
Baker, 2004	Total cost with screening for women and partner	1,267.24	1,661.04

For peer review only

Barnabas, 2002	Maternal mortality cost	443,858.00	616,230.57
Thung, 2005	Mortality cost	13,202.00	17,304.61
Barnabas, 2002	Neonatal care after caesarean	885.00	1228.69
Barnabas, 2002	Medical services for care of neonatal herpes	273,712.00	380,008.25
Barnabas, 2002	Long term care for neonatal herpes	140,766.00	195,432.58
Barnabas, 2002	Caregiver cost for neonates due to neonatal herpes	149,943.00	208,173.47

For peer review only

Figure 1: Methodological quality of included economic studies using CHEC Checklist

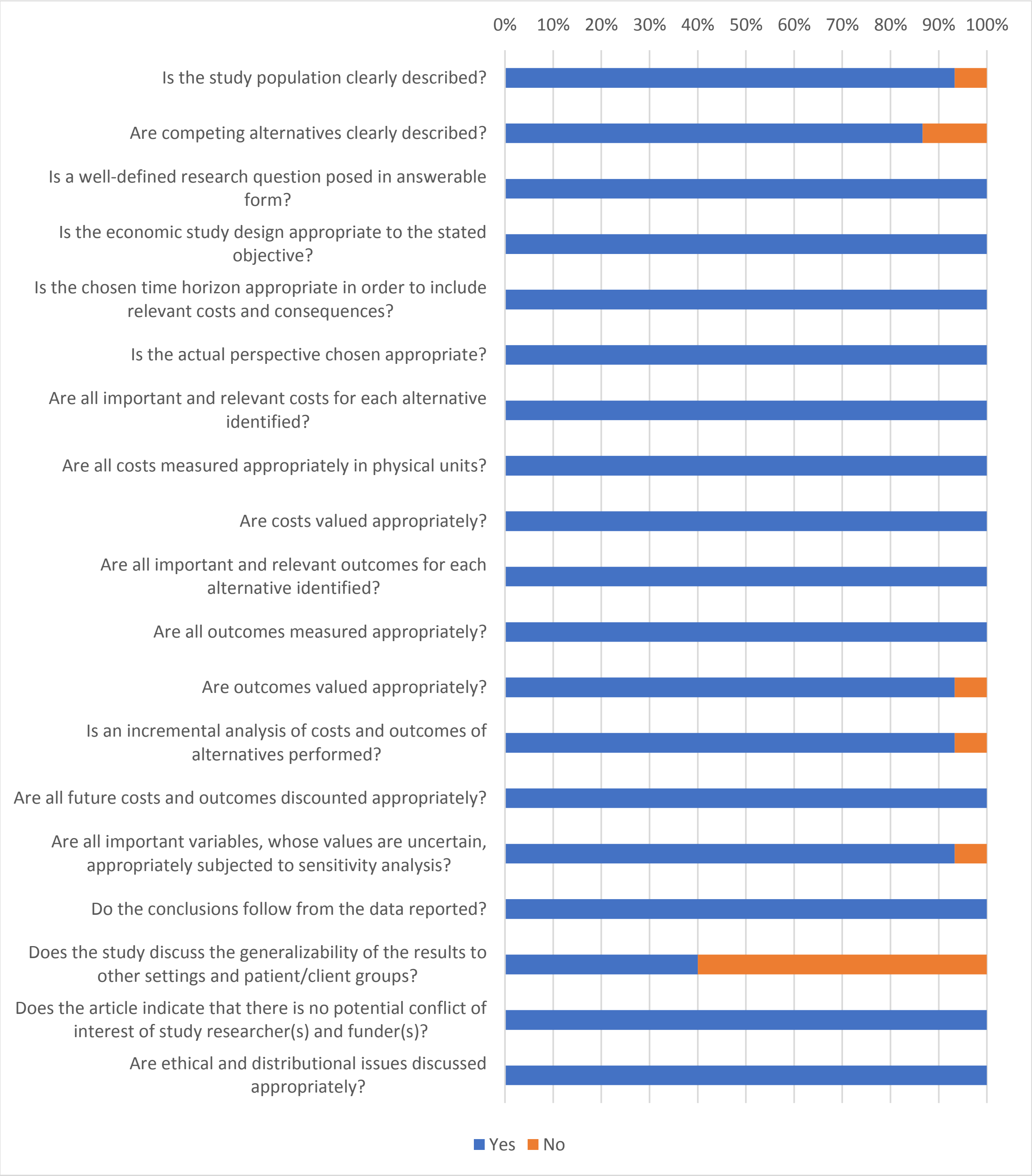

Figure 2: Methodological quality of included costing studies using Larg and Moss Checklist

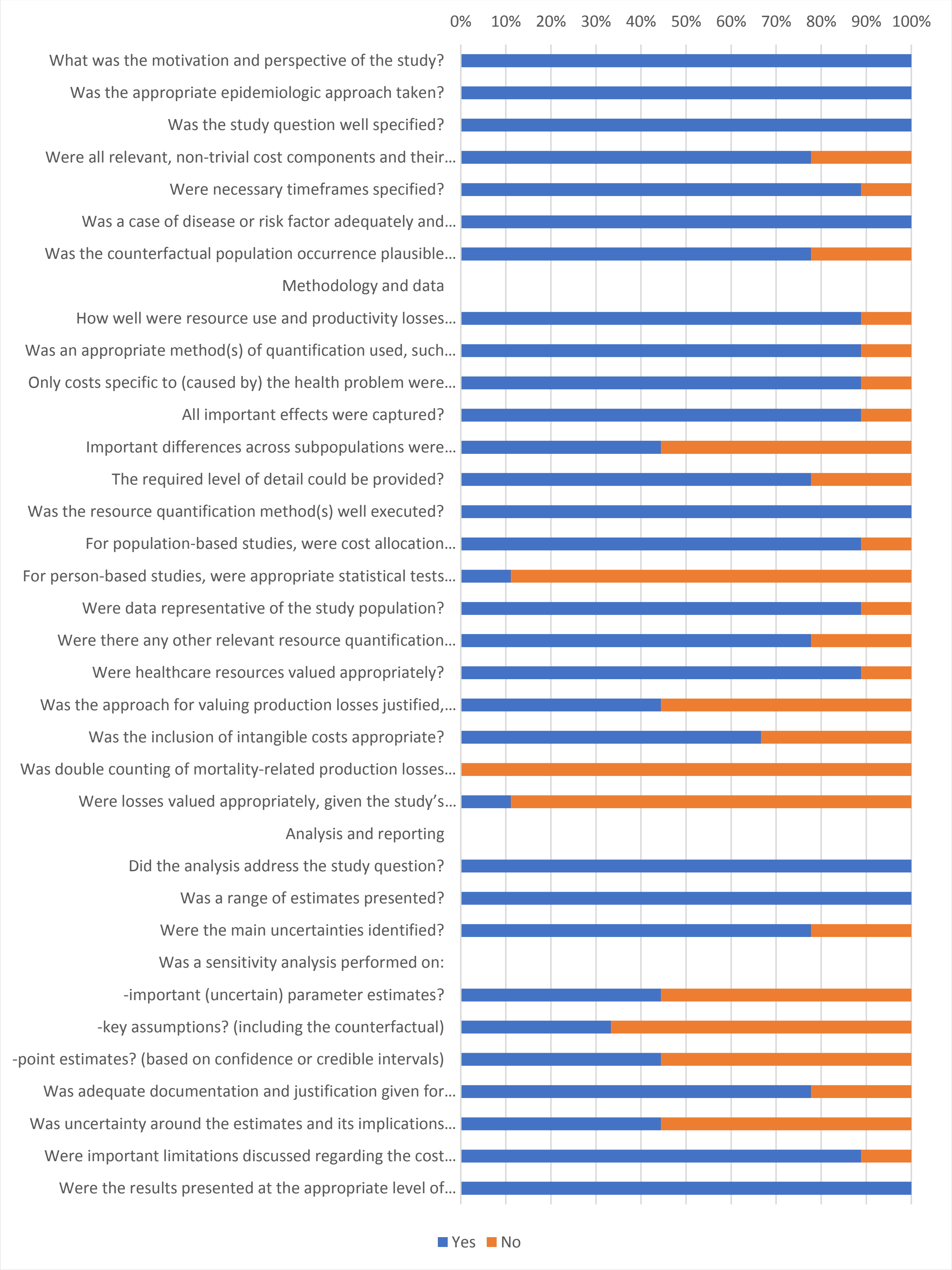

PRISMA 2020 Checklist

Section and Topic	Item #	Checklist item	Location where item is reported
**TITLE**			
Title	1	Identify the report as a systematic review.	1
**ABSTRACT**			
Abstract	2	See the PRISMA 2020 for Abstracts checklist.	Attachment
**INTRODUCTION**			
Rationale	3	Describe the rationale for the review in the context of existing knowledge.	5
Objectives	4	Provide an explicit statement of the objective(s) or question(s) the review addresses.	5-6
**METHODS**			
Eligibility criteria	5	Specify the inclusion and exclusion criteria for the review and how studies were grouped for the syntheses.	7
Information sources	6	Specify all databases, registers, websites, organisations, reference lists and other sources searched or consulted to identify studies. Specify the date when each source was last searched or consulted.	7
Search strategy	7	Present the full search strategies for all databases, registers and websites, including any filters and limits used.	7, Appendix
Selection process	8	Specify the methods used to decide whether a study met the inclusion criteria of the review, including how many reviewers screened each record and each report retrieved, whether they worked independently, and if applicable, details of automation tools used in the process.	7
Data collection process	9	Specify the methods used to collect data from reports, including how many reviewers collected data from each report, whether they worked independently, any processes for obtaining or confirming data from study investigators, and if applicable, details of automation tools used in the process.	7-8
Data items	10a	List and define all outcomes for which data were sought. Specify whether all results that were compatible with each outcome domain in each study were sought (e.g. for all measures, time points, analyses), and if not, the methods used to decide which results to collect.	8
	10b	List and define all other variables for which data were sought (e.g. participant and intervention characteristics, funding sources). Describe any assumptions made about any missing or unclear information.	8
Study risk of bias assessment	11	Specify the methods used to assess risk of bias in the included studies, including details of the tool(s) used, how many reviewers assessed each study and whether they worked independently, and if applicable, details of automation tools used in the process.	8
Effect measures	12	Specify for each outcome the effect measure(s) (e.g. risk ratio, mean difference) used in the synthesis or presentation of results.	8
Synthesis methods	13a	Describe the processes used to decide which studies were eligible for each synthesis (e.g. tabulating the study intervention characteristics and comparing against the planned groups for each synthesis (item #5)).	8
	13b	Describe any methods required to prepare the data for presentation or synthesis, such as handling of missing summary statistics, or data conversions.	8
	13c	Describe any methods used to tabulate or visually display results of individual studies and syntheses.	8
	13d	Describe any methods used to synthesize results and provide a rationale for the choice(s). If meta-analysis was performed, describe the model(s), method(s) to identify the presence and extent of statistical heterogeneity, and software package(s) used.	8
	13e	Describe any methods used to explore possible causes of heterogeneity among study results (e.g. subgroup analysis, meta-regression).	8
	13f	Describe any sensitivity analyses conducted to assess robustness of the synthesized results.	NA
Reporting bias assessment	14	Describe any methods used to assess risk of bias due to missing results in a synthesis (arising from reporting biases).	8
Certainty	15	Describe any methods used to assess certainty (or confidence) in the body of evidence for an outcome.	NA

For peer review only - http://bmjopen.bmj.com/site/about/guidelines.xhtml

For peer review only

PRISMA 2020 Checklist

Section and Topic	Item #	Checklist item	Location where item is reported
assessment			
**RESULTS**			
Study selection	16a	Describe the results of the search and selection process, from the number of records identified in the search to the number of studies included in the review, ideally using a flow diagram.	9
	16b	Cite studies that might appear to meet the inclusion criteria, but which were excluded, and explain why they were excluded.	9, Appendix
Study characteristics	17	Cite each included study and present its characteristics.	9
Risk of bias in studies	18	Present assessments of risk of bias for each included study.	9
Results of individual studies	19	For all outcomes, present, for each study: (a) summary statistics for each group (where appropriate) and (b) an effect estimate and its precision (e.g. confidence/credible interval), ideally using structured tables or plots.	10-14
Results of syntheses	20a	For each synthesis, briefly summarise the characteristics and risk of bias among contributing studies.	10-14
	20b	Present results of all statistical syntheses conducted. If meta-analysis was done, present for each the summary estimate and its precision (e.g. confidence/credible interval) and measures of statistical heterogeneity. If comparing groups, describe the direction of the effect.	10-14
	20c	Present results of all investigations of possible causes of heterogeneity among study results.	10-14
	20d	Present results of all sensitivity analyses conducted to assess the robustness of the synthesized results.	10-14
Reporting biases	21	Present assessments of risk of bias due to missing results (arising from reporting biases) for each synthesis assessed.	9-10
Certainty of evidence	22	Present assessments of certainty (or confidence) in the body of evidence for each outcome assessed.	NA
**DISCUSSION**			
Discussion	23a	Provide a general interpretation of the results in the context of other evidence.	15-17
	23b	Discuss any limitations of the evidence included in the review.	15-17
	23c	Discuss any limitations of the review processes used.	16
	23d	Discuss implications of the results for practice, policy, and future research.	16-17
**OTHER INFORMATION**			
Registration and protocol	24a	Provide registration information for the review, including register name and registration number, or state that the review was not registered.	NA
	24b	Indicate where the review protocol can be accessed, or state that a protocol was not prepared.	NA
	24c	Describe and explain any amendments to information provided at registration or in the protocol.	NA
Support	25	Describe sources of financial or non-financial support for the review, and the role of the funders or sponsors in the review.	4
Competing interests	26	Declare any competing interests of review authors.	19
Availability of data, code and other materials	27	Report which of the following are publicly available and where they can be found: template data collection forms; data extracted from included studies; data used for all analyses; analytic code; any other materials used in the review.	19

For peer review only - http://bmjopen.bmj.com/site/about/guidelines.xhtml

From: Page MJ, McKenzie JE, Bossuyt PM, Boutron I, Hoffmann TC, Mulrow CD, et al. The PRISMA 2020 statement: an updated guideline for reporting systematic reviews. BMJ 2021;372:n71. doi: 10.1136/bmj.n71

For peer review only

PRISMA 2020 Checklist

For more information, visit: http://www.prisma-statement.org/

For peer review only

PRISMA 2020 for Abstracts Checklist

YSection and Topic	Item #	Checklist item	Reported (Yes/No)
**TITLE**			
Title	1	Identify the report as a systematic review.	Yes
**BACKGROUND**			
Objectives	2	Provide an explicit statement of the main objective(s) or question(s) the review addresses.	Yes
**METHODS**			
Eligibility criteria	3	Specify the inclusion and exclusion criteria for the review.	Yes
Information sources	4	Specify the information sources (e.g. databases, registers) used to identify studies and the date when each was last searched.	Yes
Risk of bias	5	Specify the methods used to assess risk of bias in the included studies.	Yes
Synthesis of results	6	Specify the methods used to present and synthesise results.	Yes
**RESULTS**			
Included studies	7	Give the total number of included studies and participants and summarise relevant characteristics of studies.	Yes
Synthesis of results	8	Present results for main outcomes, preferably indicating the number of included studies and participants for each. If meta-analysis was done, report the summary estimate and confidence/credible interval. If comparing groups, indicate the direction of the effect (i.e. which group is favoured).	Yes
**DISCUSSION**			
Limitations of evidence	9	Provide a brief summary of the limitations of the evidence included in the review (e.g. study risk of bias, inconsistency and imprecision).	Yes
Interpretation	10	Provide a general interpretation of the results and important implications.	Yes
**OTHER**			
Funding	11	Specify the primary source of funding for the review.	Yes
Registration	12	Provide the register name and registration number.	No

From: Page MJ, McKenzie JE, Bossuyt PM, Boutron I, Hoffmann TC, Mulrow CD, et al. The PRISMA 2020 statement: an updated guideline for reporting systematic reviews. BMJ 2021;372:n71. doi: 10.1136/bmj.n71

For more information, visit: http://www.prisma-statement.org/

For peer review only
